# The genetic architecture of Parkinson's disease in Mexico: a systematic review

**DOI:** 10.3389/fnagi.2026.1709246

**Published:** 2026-02-19

**Authors:** Oscar Arias-Carrión, Elizabeth Romero-Gutiérrez, Francisco X. Castellanos-Juárez, Ada A. Sandoval-Carrillo, José M. Salas-Pacheco

**Affiliations:** 1 División de Neurociencias Clínica, Instituto Nacional de Rehabilitación Luis Guillermo Ibarra Ibarra, Mexico City, Mexico; 2 Tecnologico de Monterrey, Escuela de Medicina y Ciencias de la Salud, Mexico City, Mexico; 3 Instituto de Investigación Científica, Universidad Juárez del Estado de Durango, Durango, México

**Keywords:** ancestry-informed genomics, early-onset parkinsonism, genetic variants, *LRRK2*, Mexican population, Parkinson's disease

## Abstract

**Background:**

Despite substantial advances in Parkinson's disease genomics, Latin American populations remain underrepresented in global genetic studies, limiting the generalizability of risk estimates and biological inference. Mexico, characterized by complex admixture patterns, represents a critical setting for evaluating population-level genetic variation associated with Parkinson's disease.

**Methods:**

Following PRISMA 2020 guidelines, we systematically reviewed original studies published between 2004 and February 2025 that investigated genetic variants or gene-expression profiles in clinically diagnosed Parkinson's disease among individuals recruited in Mexico. Twenty-four studies (7,048 participants; 3,367 patients and 3,781 controls) met the inclusion criteria. Variant nomenclature was harmonized using HGNC and dbSNP identifiers. Study quality was appraised using the Q-Genie instrument, and effect estimates were standardized where feasible. Functional interpretation incorporated Gene Ontology, WikiPathways, and network-based analyses.

**Results:**

Across the included literature, 27 genes and 71 distinct genetic variants were examined. Eight loci—*PRKN, SNCA, GBA1, LRRK2, APOE, MTHFR, SYT11*, and *NR4A2*—emerged as recurrently associated with Parkinson's disease. Biallelic *PRKN* variants and exon rearrangements predominated in early-onset disease, frequently co-occurring with *PINK1* or *LRRK2* alterations. The *GBA1* p.L444P variant conferred increased risk, whereas the canonical *LRRK2* p.G2019S mutation was consistently absent. Multiple regulatory *SNCA* polymorphisms showed consistent associations across the independent Mexican cohorts examined. Additional risk-modifying variants included *APOE* ε4, *MTHFR* rs1801133, and *SYT11* variants rs34372695, rs729022, and rs822508. Protective associations were reported for *NR4A2* haplotypes—distinguishing H1 as protective and H2 as risk-increasing—and for *ALDH1A1* rs3764435. Functional integration highlighted convergence on mitochondrial quality control, lysosomal–autophagic processes, oxidative stress responses, synaptic vesicle cycling, and dopaminergic signaling.

**Conclusions:**

This systematic review provides the first quality-assessed synthesis of genetic studies of Parkinson's disease conducted in Mexico. The available evidence supports the involvement of established Parkinson's disease-related molecular pathways while underscoring substantial methodological heterogeneity and limited ancestry-aware analyses. Larger, well-powered genome-wide and multi-omic studies incorporating explicit ancestry modeling are required to refine genetic risk architecture and improve the interpretability of Parkinson's disease genomics in Mexican populations.

## 1 Introduction

Parkinson's disease (PD) is a progressive neurodegenerative disorder defined by cardinal motor features and supported by a growing body of evidence implicating both genetic and environmental determinants (Romero-Gutierrez et al., 2021; Santana-Roman et al., 2025a,b). Over the past two decades, advances in sequencing technologies and large-scale genome-wide association studies have reshaped the aetiological framework of PD from a model dominated by rare Mendelian mutations in genes such as *SNCA, PRKN*, and *LRRK2* to one increasingly characterized by polygenic susceptibility and regulatory variation acting across mitochondrial, lysosomal and synaptic pathways (Kim et al., 2024). However, despite these global gains, the field remains limited by pronounced ancestral imbalance. Individuals of European descent constitute the overwhelming majority of participants in PD genomics, constraining the discovery of ancestry-specific risk and protective alleles and reducing the transferability of genetic effect estimates to the world's predominantly non-European populations.

Latin American populations illustrate this disparity. Although initiatives such as LARGE-PD have provided important regional insights, Latin America remains markedly underrepresented in global PD genetics (Loesch et al., 2022; Leal et al., 2025). Mexico, in particular, occupies a pivotal position: it comprises one of the largest PD-affected populations in the region and is characterized by a distinctive tri-hybrid admixture of Indigenous, European and African ancestries. This demographic history has generated patterns of allele frequency, linkage disequilibrium and local ancestry structure that diverge substantially from those of European reference cohorts (Romero-Gutierrez et al., 2021; Lazaro-Figueroa et al., 2025). These features create conditions under which population-enriched variants, ancestry-modulated mechanisms of neurodegeneration and context-specific protective factors may be detectable, yet they also mean that findings from European-centric studies may not generalize reliably to Mexican populations.

Despite nearly two decades of genetic studies conducted in Mexico, no comprehensive, PRISMA-guided synthesis of this body of work currently exists. Prior regional reviews, including a recent Latin American overview of sporadic PD, which examined early-onset cases, identified monogenic pathogenic variants, and incorporated copy-number analyses, nonetheless did not include cohorts recruited in Mexico and therefore lacked country-specific resolution for this population (Lorenzo-Betancor et al., 2024). A recent systematic review of sporadic PD across Latin America (Duarte-Zambrano et al., 2025) similarly provides valuable regional context but does not integrate Mexico-specific data or employ network-based analytical approaches, underscoring the need for a focused synthesis.

Furthermore, earlier reviews did not employ pathway-level or systems-oriented methodologies capable of determining whether reported variants converge onto shared biological mechanisms. Notably, prior syntheses of PD genetics in Latin America, including region-wide summaries of sporadic PD risk variants, surveyed candidate-gene findings but did not undertake network-based integration or functional-pathway reconstruction (Loesch et al., 2022). As a result, these studies provided valuable descriptive catalogs yet lacked the analytical depth needed to contextualize genetic signals within mitochondrial, lysosomal, and synaptic regulatory frameworks.

The present systematic review addresses this gap by consolidating and critically appraising all published genetic and transcriptomic studies of PD in Mexican populations conducted since 2004. Using the PRISMA 2020 methodology, we harmonized variant nomenclature, standardized effect-size reporting, and assessed methodological quality using the Q-Genie instrument. To provide systems-level insight, we further integrated the curated variant set into functional interaction networks using Cytoscape and pathway resources including Gene Ontology, WikiPathways, and KEGG. This integrative strategy was designed to preserve study-level heterogeneity while enabling biologically coherent interpretation across diverse candidate-gene and observational designs.

This study pursues two central questions: which genetic variants—across monogenic, risk-modifying, protective, and structural categories—have been reported to be associated with PD among Mexican individuals, and what biological pathways and molecular networks emerge when these variants are examined within established functional-interaction frameworks. Accordingly, the objectives of this review are to compile all PD-associated genetic variation reported in Mexican cohorts systematically and to characterize the resulting gene set within multiscale molecular networks, without presupposing ancestry-specific effects in the absence of formal ancestry-aware analyses. By grounding the work in a clearly defined rationale, research questions and objectives, this review establishes a foundation for future ancestry-informed PD genomics and provides the first integrated map of the genetic architecture of PD in Mexico.

## 2 Methods

### 2.1 Study design and eligibility

This systematic review was conducted in accordance with PRISMA 2020 guidelines and was designed to synthesize all peer-reviewed genetic association and transcriptomic studies involving PD cases recruited in Mexico. Eligibility criteria were defined strictly on the basis of recruitment setting rather than ancestry descriptors, thereby avoiding the conceptual conflation of national origin with genetic ancestry. Studies were included if they: (i) examined genetic variation—such as single-nucleotide variants, structural variants, copy-number alterations, or differential gene-expression profiles—in individuals with clinically diagnosed PD residing in Mexico; (ii) employed recognized diagnostic criteria, including the UK Parkinson's Disease Society Brain Bank or Movement Disorder Society (MDS) standards; and (iii) reported extractable data, including genotype counts, allele frequencies, effect estimates, or transcriptomic statistics. Eligible study designs encompassed observational case–control studies, candidate-gene screens, sequencing-based analyses, and transcriptomic profiling.

**Figure 1:**
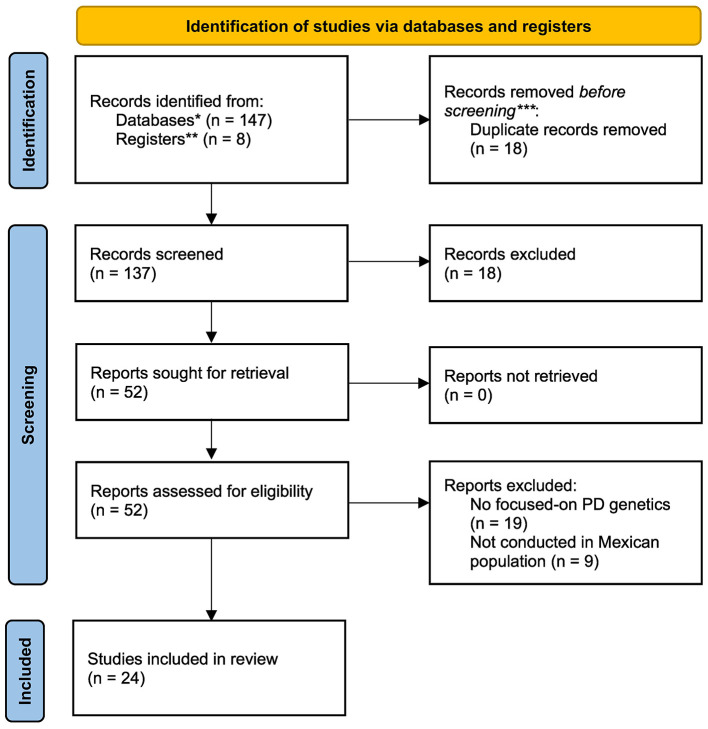
PRISMA 2020 flow diagram of study selection. This flow diagram summarizes the identification, screening, eligibility assessment and inclusion of studies in accordance with PRISMA 2020 guidelines. A total of 155 records were identified (147 from databases and 8 from additional sources). After removing 18 duplicates, 137 records underwent title and abstract screening. Fifty-two full-text articles were assessed for eligibility, of which 24 met the inclusion criteria. Full-text exclusions were based on a lack of focus on Parkinson's disease genetics (*n* = 19) or study populations not conducted in Mexican cohorts (*n* = 9). Included studies reported original genetic or gene-expression data from clinically diagnosed individuals with Parkinson's disease of Mexican ancestry. *Databases: s PubMed/MEDLINE, Scopus, Web of Science Core Collection, and Google Scholar. **Other sources: Reference list and expert consultations. ***Excluded by the human screening.

Exclusion criteria included studies conducted entirely outside Mexico, those enrolling non-Mexican or expatriate populations, absence of extractable genetic or expression data, lack of formal PD diagnostic criteria, and any form of non-primary literature (conference abstracts, reviews, theses, or gray literature). These determinations reflect the sequential filtering process summarized in Figure 1, in which 155 records were identified, 18 duplicates removed, 137 titles and abstracts screened, 52 full texts assessed for eligibility, and 24 studies ultimately included.

### 2.2 Search strategy and study identification

A comprehensive and exhaustive literature search strategy was implemented across PubMed/MEDLINE, Scopus, Web of Science Core Collection, and Google Scholar to identify all eligible genetic association and transcriptomic studies of PD conducted in Mexico and published between 1 January 2004 and 28 February 2025. This time frame corresponds to the period during which molecular genetics, SNP-based genotyping, copy-number analysis, and transcriptomic approaches became progressively established within Mexican biomedical research, and it captures the entirety of the modern PD genetics literature generated in this setting.

To ensure full methodological transparency and reproducibility, the complete database-specific search strategies—including Boolean logic, MeSH terminology, controlled vocabulary, field tags, wildcards, and free-text synonyms—are reported in *the section titled* “*Complete Database-Specific Search Strategies*,” in accordance with PRISMA 2020 Item 7. The search strategy was iteratively refined to maximize sensitivity for studies conducted in Mexico, including early candidate-gene investigations and publications in journals with regional or multilingual dissemination.

Because Google Scholar does not support advanced Boolean operators or field-restricted searches, a reproducible free-text query was employed, and the first 200 records ranked by relevance were systematically screened, following established methodological recommendations for its use in systematic reviews. No language restrictions or document-type filters were applied in any database to maximize the retrieval of original studies, including those published in Spanish or in regionally indexed journals.

In addition to database searching, systematic backwards and forward citation tracking was performed for all included studies and relevant reviews. This approach was particularly important given the relatively small number of research groups conducting PD genetics studies in Mexico over the past two decades and the dispersion of original contributions across journals with heterogeneous indexing practices. This combined strategy was designed to ensure that early foundational studies, low-frequency genetic findings, and structurally oriented investigations were not lost due to publication language, journal visibility, or indexing limitations.

All titles and abstracts were screened independently by two reviewers (O. A.-C. and E. R.-G.), followed by full-text assessment of potentially eligible articles. Discrepancies were resolved by consensus. As summarized in Figure 1, 137 unique records were screened, 52 full-text articles were assessed for eligibility, and 24 studies satisfied all inclusion criteria and were included in the final synthesis.

### 2.3 Data extraction and harmonization

Data extraction was performed independently by two reviewers using a structured, prespecified template designed to maximize reproducibility and ensure comparability across heterogeneous study designs. For each included article, we systematically extracted information on study design, recruitment setting, geographical region, inclusion criteria, sample size, demographic characteristics, diagnostic approach, genetic targets interrogated, genotyping or sequencing methodology, allele and genotype frequencies, statistical modeling strategies, and reported effect estimates.

All study-level characteristics are reported in Supplementary Table 1, where each study is represented by a single row detailing methodological attributes, outcome definitions, analytical parameters, and item-specific Q-Genie domain scores. This format reflects the structure of the Q-Genie quality assessment instrument, which was specifically developed for genetic association studies and emphasizes transparent reporting of study design, analytical validity, and inferential appropriateness rather than quantitative weighting across studies.

Variant nomenclature was harmonized using HGNC gene symbols and dbSNP identifiers. Because several studies reported only raw allele counts or genotype frequencies without adjusted effect estimates, unadjusted odds ratios and 95% confidence intervals were recalculated to facilitate descriptive comparison across studies. All recalculated estimates are explicitly identified in Supplementary Table 1 together with the corresponding Q-Genie appraisal, allowing readers to contextualize effect sizes in light of study quality and methodological limitations. Given the absence of covariate adjustment in these recalculated metrics and the substantial heterogeneity across studies—including differences in case–control matching, population sampling, and analytical design—these estimates were used exclusively for qualitative synthesis. No quantitative pooling or meta-analysis was undertaken.

To avoid overstating ancestry-related interpretations, cross-population contrasts were restricted to descriptive comparisons of allele frequencies and reported effect sizes from the included literature and global datasets. Signals were not designated as ancestry-specific unless formal ancestry analyses were conducted in the original study. This approach aligns with the Q-Genie framework and reflects the methodological diversity of the available evidence.

### 2.4 Data synthesis and functional integration

Given the heterogeneity in study design, statistical reporting, and analytical depth, we employed a narrative synthesis approach. Genes and variants were grouped by recurrence, biological plausibility, and clinical classification (monogenic, risk-modifying, protective, or non-associated). To contextualize isolated variant findings within higher-order biological systems, we performed pathway and network integration using Gene Ontology (GO: Biological Process), WikiPathways, KEGG, and Cytoscape v3.10. Functional relationships were visualized through bipartite and process-level network representations, enabling interpretation of convergence across mitochondrial quality control, autophagy–lysosomal function, proteostasis, and dopaminergic neurotransmission. These analyses were descriptive and do not imply causality.

### 2.5 Quality assessment

Risk of bias was assessed using the Q-Genie tool, applied independently by two reviewers (O. A.-C. and E. R.-G.), in accordance with PRISMA item 11 and best practices for genetic association studies. Each study received item-level and aggregate scores across Q-Genie's 11 domains. Discrepancies were resolved by consensus following structured Discussion. All domain-specific and total scores are reported in Supplementary Table 2, which informs interpretive weighting without serving as exclusion criteria.

## 3 Results

### 3.1 Study selection and characteristics

The search process yielded 155 records, of which 18 duplicates were removed prior to screening. After review of 137 titles and abstracts and 52 full-text articles, 24 studies met the inclusion criteria (Figure 1). Across the 24 eligible studies conducted between 2004 and 2021, a total of 7,048 participants; 3,367 PD patients and 3,781 controls subjects were recruited in Mexico (Martinez et al., 2004; Ramirez-Jirano et al., 2006; Lopez et al., 2007; Ramírez-Jirano et al., 2007; Gallegos-Arreola et al., 2009; Martinez et al., 2010; Yescas et al., 2010; Dávila-Ortiz de Motellano et al., 2012; Guerrero Camacho et al., 2012; Gonzalez-Del Rincon Mde et al., 2013; Garcia et al., 2014; Monroy-Jaramillo et al., 2014; Cervantes-Arriaga et al., 2015; Garcia et al., 2015; Davila-Ortiz de Montellano et al., 2016; Garcia et al., 2016; Sesar et al., 2016; Garcia et al., 2017; Ruiz-Sanchez et al., 2017; Garcia et al., 2019; Miranda-Morales et al., 2019; Salas-Leal et al., 2019; Romero-Gutierrez et al., 2021; Salas-Leal et al., 2021). The study-level characteristics and Q-Genie quality scores are summarized in Supplementary Table 1.

**Figure 2:**
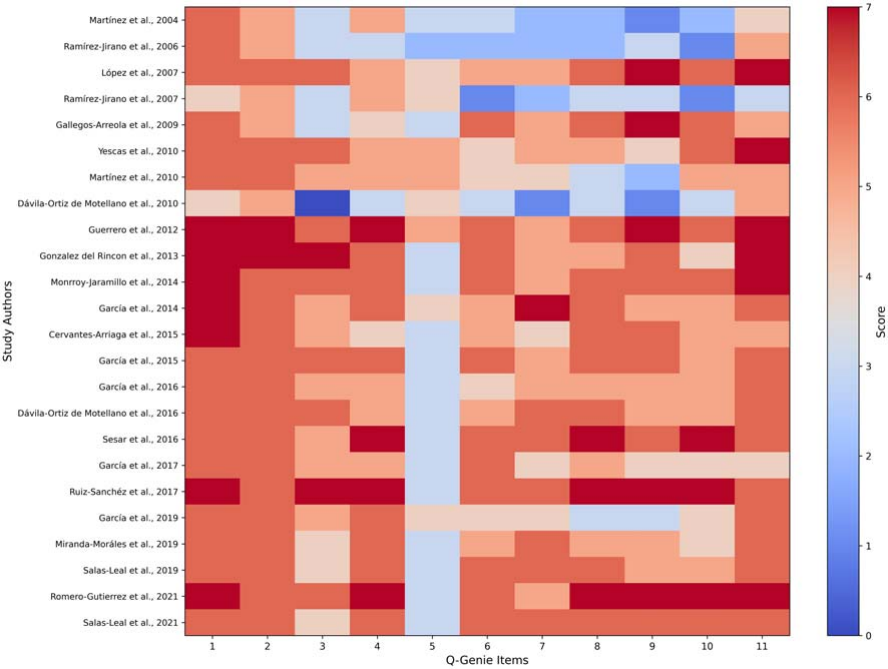
Quality assessment of genetic association studies using the Q-Genie instrument. Each cell represents the score assigned to an individual study (rows) across the 11 Q-Genie domains (columns). Colors denote relative quality for each methodological criterion, ranging from low (blue) to high (red). The Q-Genie tool evaluates critical components of study design, including scientific rationale, case and control selection, group comparability, genotyping reliability, statistical methods, and interpretation of findings. Higher domain scores indicate stronger methodological rigor and improved reproducibility, whereas lower scores highlight weaknesses in design, statistical power, or analytical consistency. Among the 24 studies included (2004–2021), 72% were classified as high quality, 24% as moderate quality, and 4% as low quality, based on established Q-Genie thresholds. This appraisal provided a structured basis for evaluating the robustness of the evidence synthesized in Tables 2–4.

Quality appraisal indicated substantial heterogeneity across methodological domains (Figure 2). Later studies tended to incorporate more rigorous statistical modeling, genotype quality control, and explicit reporting of allele frequencies, whereas earlier candidate-gene investigations frequently omitted details on power calculation, confounder adjustment, and blinding. Case definitions and diagnostic criteria were consistently strong across all studies.

### 3.2 Variant spectrum in Mexican PD cohorts

Across all included investigations, 27 genes and 71 variants were examined (Table 1). The most frequently interrogated loci were *PRKN, PINK1, SNCA, GBA1, LRRK2, APOE, MTHFR, SYT11*, and *NR4A2*, reflecting emphasis on mitochondrial maintenance, lysosomal–autophagic pathways, synaptic regulation, and dopaminergic differentiation.

**Table 1:** Characteristics of genetic studies of Parkinson's disease conducted in Mexico (2004–2021).

**Gene**	**Variant**	**Groups, *n***	**Allele and genotype frequency in%. PD vs. controls**	**p^allelic^/ p^genotype^**	**OR (95% CI), *p*-value**	**References**
*PRKN*	Exons 2, 4, 6, 9, 11 Polymorphisms	50 PD 60 Controls	0 vs. 0	NR	NR	Martinez et al., 2004
*PRKN*	Exon 3 Polymorphisms	50 PD 60 Controls	20 vs. 0	NR	NR	Martinez et al., 2004
*PRKN*	Exon 7 Polymorphisms	50 PD 60 Controls	2 vs. 0	NR	NR	Martinez et al., 2004
*SNCA*	−116C → G	51 iPD 121 Controls	G: 74.5 vs. 78.8 GG: 56.9 vs. 59.4 CG: 35.3 vs. 38.7 CC: 7.8 vs. 1.9	*>0.05*	NR	Ramirez-Jirano et al., 2006
*APOE*	ε2, ε3, ε4	229 PD 229 Controls	ε2: 4.4 vs. 3.7 ε3: 83.2 vs. 88.4 ε4: 12.4 vs. 7.9 ε2/ε3: 8.3 vs. 6.6 ε3/ε3: 68. vs. 78.2 ε4/ε2: 0.4 vs. 0.9 ε4/ε3: 21.8 vs. 14 ε4/ε4: 1.3 vs. 0.4	NR	ε4: 1.736, ***p 0.011*** ε4/ε3: 1.688, ***p 0.019***	Lopez et al., 2007
*SNCA*	IVS4+66A → G	51 PD 121 Controls	A: 70.6 vs. 67.4 G: 29.4 vs. 32.6 AA: 43.1 vs. 38.8 AG: 54.9 vs. 57.1 GG: 2.0 vs. 4.1	*0.55 0.47*	0.86 (0.5–1.46), *p* NR 0.46 (0.02–4.26) *p* NR	Ramírez-Jirano et al., 2007
*APOE*	ε2, ε3, ε4	105 PD 107 Controls	ε2: 6 vs. 2.3 ε3: 73 vs. 88.3 ε4: 21 vs. 9.4 ε2/ε2: 4 vs. 1 ε2/ε3: 3 vs. 0 ε3/ε3: 52 vs. 80 ε2/ε4: 2 vs. 3 ε3/ε4: 38 vs. 16 ε4/ε4: 1 vs. 0	NR NR	ε2: 2.76 (0.90–10.04), *p* 0.08 ε3: 0.36 (0.20–0.61), ***p 0.001*** ε4: 2.57 (1.42–4.79), ***p 0.001*** ε2/ε2: 2.55 (0.4–27.20), *p 0.44* ε3/ε3: 0.27 (0.14–0.52), ***p 0.001*** ε4/ε4: 2.06 (0.11–122.47), *p 0.61* ε2/ε3: 4.19 (0.40–208.03), *p 0.21* ε2/ε4: 0.67 (0.06–6.01), *p 1.00* ε3/ε4: 3.26 (1.63–6.66), ***p 0.001***	Gallegos-Arreola et al., 2009
*LRRK*	rs33939927 c.4321C → G p.Arg1441Gly	319 PD 200 Controls	CG: 0.3 vs. 0	NR	NR	Yescas et al., 2010
*LRRK2*	rs34995376 c.4322G → A p.Arg1441His	319 PD 200 Controls	GA: 0.3 vs. 0	NR	NR	Yescas et al., 2010
*LRRK2*	rs34637584 c.6055A → G p.Gly2019Ser	319 PD 200 Controls	AG 0.3 vs. 0	NR	NR	Yescas et al., 2010
*PRKN*	c.601G → A p.Ser167Asn	117 PD 122 Controls	G: 79.5 vs. 84 A: 20.5 vs. 16 GG: 65.8 vs. 74.6 GA: 27.4 vs. 18.8 AA: 6.8 vs. 6.6	*0.281* With frequency of 49% in group early-onset cases with family history.	NA	Martinez et al., 2010
*PRKN*	c.1138G → C p.Val380Leu	117 PD 122 Controls	G: 88.9 vs. 81.8 C: 11.1 vs. 18.2 GG: 82.0 vs. 71 GC: 13.7 vs. 21.5 CC: 4.3 vs. 7.4	*0.141* With frequency of 49% in group early-onset cases with family history.	NA	Martinez et al., 2010
*PRKN*	c.1197C → T p.Arg366Trp	117 PD 122 Controls	CC: 99.15 vs. 100 CT: 0.85 vs. 0 TT: 0 vs. 0	NA	NA	Martinez et al., 2010
*PRKN*	c.1281G → A p.Asp394Asn	117 PD 122 Controls	GG: 99.15 vs. 100 GA: 0.85 vs. 0 AA: 0 vs. 0	NA	NA	Martinez et al., 2010
*SNCA*	p.Ala53Thr	86 ADPD	ND	NA	NA	Dávila-Ortiz de Motellano et al., 2012
*SNCA*	p.Ala30Pro	86 ADPD	ND	NA	NA	Dávila-Ortiz de Motellano et al., 2012
*SNCA*	p.Glu46Lys	86 ADPD	ND	NA	NA	Dávila-Ortiz de Motellano et al., 2012
*PRKN*	Exonic CNVs and small indels	63 EOPD 120 Controls	CNVs and small indels 54 vs. 0 CNV's in Exons 2–3 17.5 vs. 0.83	NA ***p*** **=** **0.0006**	NR	Guerrero Camacho et al., 2012
*GBA1*	rs421016 c.1448T → C p.Leu444Pro	128 EOPD 252 Controls	TC: 5.5 vs. 0	**0.014**	NR	Gonzalez-Del Rincon Mde et al., 2013
*GBA1*	rs76763715 c.1226A → G p.Asn370Ser	128 EOPD 252 Controls	AA: 100 vs. 100	NA	NA	Gonzalez-Del Rincon Mde et al., 2013
*PRKN*	CNVs and point mutations	122 EOPD 120 Controls	20.5 vs. 0.83	Frequently in EOPD	NR	Monroy-Jaramillo et al., 2014
*PINK1*	CNVs and point mutations	122 EOPD 120 Controls	9 vs. 1.6	Frequently in EOPD	NR	Monroy-Jaramillo et al., 2014
*PRKN7*	CNVs and point mutations	122 EOPD 120 Controls	0.8 vs. 0	Frequently in EOPD	NR	Monroy-Jaramillo et al., 2014
*Digenic variants*	PRKN-LRRK2 Delx-rs34637584	122 EOPD 120 Controls	0.8 vs. 0	Frequently in EOPD	NR	Monroy-Jaramillo et al., 2014
*Digenic Variants*	PRKN-PINK1 Delx-Delx	122 EOPD 120 Controls	1.16 vs. 0	Frequently in EOPD	NR	Monroy-Jaramillo et al., 2014
*LRRK2*	rs34637584 c.6055G → A p.Gly2019Ser	173 sPD 208 Controls	1.15 vs. 0.48	NR	NR	Garcia et al., 2014
*LRRK2*	rs34778348 c.7154G → A p.Gly2385Arg	173 sPD 208 Controls	0 vs. 0	NR	NR	Garcia et al., 2014
*MTHFR*	rs1801133 c.677C → T p.Ala222Val	140 sPD 216 Controls	C/C: 27 vs. 17 T/C: 51 vs. 55 T/T: 22 vs. 28	**0.02**	REC: 2.06 (1.01–3.87) ***p*** **0.024**	Garcia et al., 2015
*ANKK1*	rs1800497 c.2137G → A A2/A1 allele	236 PD	A1/A1: 16.9 A1/A2: 69.5 A2/A2: 13.6	>0.05	NR	Cervantes-Arriaga et al., 2015
*SNCA*	rs3857059 A → G Intronic SNP	106 PD 135 Controls Female Subgroup 31 PD 46 Controls	A/A: 23.6 vs. 35.6 A/G: 52.8 vs. 49.6 G/G: 23.6 vs. 14.8 Female Subgroup A/A: 9.7 vs. 39.1 A/G: 50 vs. 50 G/G: 32.3 vs. 10.9	NR **0.004**	REC: 2.40 (1.12–5.14), ***p*** **0.02** Female Subgroup REC: 1.31 (1.01–1.7), ***p*** **0.037**	Garcia et al., 2016
*SNCA*	rs356220 C → T Intronic SNP	171 PD 171 Controls	MAF: 48.5 vs. 31	NR	2.10 (1.35–3.27) ***p*** **0.0024**	Davila-Ortiz de Montellano et al., 2016
*SNCA*	rs356203 G → A Intronic SNP	171 PD 171 Controls	MAF: 45 vs. 33.9	NR	1.60 (1.03–2.47) ***p*** **0.035**	Davila-Ortiz de Montellano et al., 2016
*SNCA*	rs7684318 T → C Intronic SNP	171 sPD 171 Controls	MAF: 69.6 vs. 18.7	NR	9.94 (5.99–16.5), ***p*** **<** **0.001**	Davila-Ortiz de Montellano et al., 2016
*SNCA*	rs2736990 C → T Intronic SNP	171 sPD 171 Controls	MAF: 52 vs. 31	NR	2.42 (1.55–3.76), ***p*** **<** **0.001**	Davila-Ortiz de Montellano et al., 2016
*SNCA*	rs2619364 A → G Intronic SNP	171 sPD 171 Controls	MAF: 71.4 vs. 77.8	NR	0.71 (0.44–1.16), *p* 0.17	Davila-Ortiz de Montellano et al., 2016
*SNCA*	Haplotype: rs356220, rs356203, rs7684318, rs2736990	171 sPD 171 Controls	CGTC: 0.1978 CATC: 0.0694 CGCC: 0.0258 TGTC: 0.0251 TACC: 0.0192 CACT: 0.0178	NR	1.92 (1.18–3.13), ***p*** **0.009** 2.54 (1.37–4.72), ***p*** **0.003** 14.51 (1.68–125.15), ***p*** **0.016** 8.97 (2.05–39.19), ***p*** **0.003** 4.71 (1.16–19.5), ***p*** **0.031** 16.18 (1.88–139.18), ***p*** **0.012**	Davila-Ortiz de Montellano et al., 2016
*SYT11*	rs822508 T → C Intronic SNP	271 PD 260 Controls	MAF(C): 46.4	NR	1.33 (1.05–1.68), ***p*** **0.017**	Sesar et al., 2016
*SYT11*	rs729022 A → G 3′UTR SNP	271 PD 260 Controls	MAF(A): 46.0	NR	1.29 (1.02–1.64), ***p*** **0.032**	Sesar et al., 2016
*SYT11*	rs12563627 T → C SNP	271 PD 260 Controls	MAF(C): 44.9	NR	1.32 (1.04–1.68), *p* 0.242	Sesar et al., 2016
*SYT11*	rs34372695 C → T SNP	271 PD 260 Controls	MAF: Imputed	NR	2.07 (1.24–3.87), ***p*** **0.00003**	Sesar et al., 2016
*MTHFR*	rs13306560 A → G Promoter SNP	113 sPD 124 Controls	A: 0.88 vs. 4.4 G: 99.12 vs. 95.56 A/A: 0.00 vs. 0.81 A/G: 1.77 vs. 7.26 G/G: 98.23 vs. 91.94	NR	DOM: 0.21 (0.042–1.05), *p* 0.058	Garcia et al., 2017
*NR4A2*	rs34884856 2C⇄3C DELINS Promotor regions	227 sPD 454 Controls	2C: 53.3 vs. 50.5 3C: 46.7 vs. 49.5 2C/2C: 31.3 vs. 26.4 2C/3C: 44.1 vs. 48.2 3C/3C: 24.7 vs. 25.3	0.258	DOM: 1.34 (0.94–1.90), *p* 0.10 REC: 0.92 (0.64–1.33), *p* 0.66 HeM: 0.84 (0.61–1.16), *p* 0.29	Ruiz-Sanchez et al., 2017
*NR4A2*	rs35479735 2G ⇄ 3G DELINS Intron 6 regions	227 sPD 454 Controls	2G: 45.4 vs. 53.6 3G: 54.6 vs. 46.4 2G/2G: 22.5 vs. 30.4 3G/2G: 45.8 vs. 46.5 3G/3G: 31.7 vs. 23.1	**0.021**	DOM: 0.67 (0.46–0.97), ***p*** **0.035** REC: 1.53 (1.08–2.19), ***p*** **0.018** HeM: 0.97 (0.70–1.33), *p* 0.846	Ruiz-Sanchez et al., 2017
*NR4A2*	Haplotype DELINS: rs34884856, rs35479735	227 sPD 454 Controls	3C-2G: 40.2 vs. 46.1 2C-3G: 48.1 vs. 41.9 2C-2G: 5.2 vs. 7.5 3C-3G: 6.5 vs. 4.5	NR	0.78 (80.62–0.98), ***p*** **0.037** 1.28 (1.02–1.60), ***p*** **0.030** 0.69 (0.44–1.11), *p* 0.106 1.53 (0.95–2.50), *p* 0.102	Ruiz-Sanchez et al., 2017
*MT-TQ*	rs41456348 m.4336 T → C Mitochondrial tRNA SNP	175 PD 194 Controls	C: 0.57 vs. 0	NR	3.402 (0.13–84.07), *p* 0.27	Garcia et al., 2019
*MT-ATP6*	rs2000975 m.8701A → G Mitochondrial SNP, p.Thr59Ala	154 PD 211 Controls	G: 77.92 vs. 71.56	NR	0.71 (0.43–1.15), *p* 0.84	Garcia et al., 2019
*MAPT*	Haplotype H1/H2 (238 bp indel, proxy marker)	108 PD 108 Controls	H1: 88 vs. 92 H2: 12 vs. 8 H1/H1: 78 vs. 85 H1/H2: 20 vs. 14 H2/H2: 2 vs. 1	0.148 0.363	H2: 1.60 (0.84–3.04), *p* 0.15 H1/H2: 1.60 (0.78–3.29) *p* 0.36 H2/H2: 2.26 (0.20–25.78)	Miranda-Morales et al., 2019
*ALDH1A1*	rs3764435 A → C Intronic SNP	120 PD 178 Controls Male Subgroup 50 PD 98 Controls	A: 0.57 vs. 0.48 C: 0.43 vs. 0.52 A/A: 0.28 vs. 0.23 A/C: 0.59 vs. 0.49 C/C: 0.13 vs. 0.28 A: 59 vs. 46 C: 41 vs. 54 A/A: 30 vs. 21 A/C: 59 vs. 49 C/C: 11 vs. 30	0.024 0.011 0.025 0.024	AA vs.AC: 1 (0.58–1.76), *p* 0.96 AA vs.CC: 0.4 (0.19–0.84), ***p*** **0.016** DOM: 0.79 (0.46–1.35), *p* 0.4 REC: 0.04 (0.21–0.75), ***p*** **0.005** Male Subgroup AAvs.AC: 0.86 (0.39–1.88), *p* 0.714 AAvs.CC: 0.40 (0.09–0.79), ***p*** **0.017** DOM: 0.64 (0.30–1.37), *p* 0.24 REC: 0.30 (0.12–0.75), ***p*** **0.011** Cog. imp. subgroup AA vs. AC: 0.34 (0.12–0.97), *p* 0.045 AA vs. CC: 0.18 (0.02–1.13), *p* 0.068 DOM: 0.30 (0.11–0.85), ***p*** **0.024** REC: 0.35 (0.66–1.94), ***p*** **0.235**	Salas-Leal et al., 2019
*LRRK2*	rs1491942 C → G Intronic SNP	118 PD 193 Controls	C: 34 vs. 47 G: 66 vs. 53 CC: 12 vs. 22 CG: 45 vs. 48 GG: 43 vs. 29	**0.03 0.01**	1.71 (1.22–2.40), ***p*** **0.002**	Romero-Gutierrez et al., 2021
*MTHFR*	rs1801133 c.677C → T p.Ala222Val	118 PD 193 Controls	C: 42 vs. 53 T: 58 vs. 47 CC: 0.19 vs. 0.29 CT: 0.45 vs. 0.48 TT: 0.36 vs. 0.23	**0.01 0.041**	1.54 (1.11–2.15), ***p*** **0.01**	Romero-Gutierrez et al., 2021
*USP24*	rs13312 C → G 3′UTR SNP	118 PD 193 Controls	G: 9 vs. 10 C: 91 vs. 90 CC: 83 vs. 82 CG: 16 vs. 16 GG: 1 vs. 2	0.99 0.99	0.91 (0.49–1.71), *p* 0.79	Romero-Gutierrez et al., 2021
*PRKN7*	rs3766606 G → T Intronic SNP	118 PD 193 Controls	T: 8 vs. 10 G: 92 vs. 90 GG: 84 vs. 81 GT: 15.1 vs. 18 TT: 0.9 vs. 1	*0.67 0.83*	0.87 (0.49–1.54), *p* = 0.64	Romero-Gutierrez et al., 2021
*NUCKS1*	rs823128 A → G Intronic SNP	118 PD 193 Controls	A: 87 vs. 88 G: 13 vs. 14 AA: 76 vs. 74 AG: 20 vs. 24 GG: 4 vs. 2	*0.90 0.56*	0.94 (0.58–1.50), *p 0.80*	Romero-Gutierrez et al., 2021
*SLC41A1*	rs823156 A → G Intronic SNP	118 PD 193 Controls	G: 22 vs, 26 A: 78 vs. 74 AA: 64 vs. 54 AG: 28 vs. 40 GG: 8 vs. 6	*0.90 0.56*	0.79 (0.54–1.17), *p 0.25*	Romero-Gutierrez et al., 2021
*GSK3B*	rs334558 A → G 5′UTR SNP	118 PD 193 Controls	A: 64 vs. 67 G: 36 vs. 33 AA: 43 vs, 45 AG: 41 vs. 47 GG: 15 vs. 10	*0.43 0.44*	1.14 (0.81–1.61), *p 0.42*	Romero-Gutierrez et al., 2021
*DRD3*	rs6280 c.25 C → T p.Ser9Gly	118 PD 193 Controls	C: 42 vs. 49 T: 58 vs. 41 TT: 36 vs. 26 TC: 44 vs. 50 CC: 20 vs. 24	*0.16 0.23*	0.75 (0.54–1.05), *p 0.10*	Romero-Gutierrez et al., 2021
*FAM47E/ SCARB2*	rs6812193 C → T Intronic SNP	118 PD 193 Controls	C: 79 vs. 82 T: 21 vs. 18 CC: 66 vs. 68 CT: 26 vs. 28 TT: 8 vs. 4	*0.04 0.31*	1.2 (0.80–1.82), *p 0.37*	Romero-Gutierrez et al., 2021
*SNCA*	rs356219 A → G Intronic SNP	118 PD 193 Controls	G: 62 vs. 56 A: 38 vs. 44 GG: 40 vs. 31 AG: 44 vs. 51 AA: 16 vs. 18	*0.2 0.29*	0.79 (0.57–1.11), *p 0.18*	Romero-Gutierrez et al., 2021
*PRKN*	rs1801474 c.500C → T^a^ p.Ser167Asn	118 PD 193 Controls	T: 15 vs. 13 C: 85 vs. 87 CC: 72 vs. 77 CT: 25 vs. 20 TT: 3 vs. 3	*0.48 0.58*	1.18 (0.78–1.87), *p 0.48*	Romero-Gutierrez et al., 2021
*PRKN*	rs1801582 c.1138G → C p.Val380Leu	118 PD 193 Controls	G: 11 vs. 16 C: 89 vs. 84 CC: 79 vs. 80 CG: 19 vs. 18 GG: 2 vs. 2	*0.73 0.94*	1.09 (0.65–1.83), *p 0.77*	Romero-Gutierrez et al., 2021
*ANKK1*	rs1800497 c.2137C → T^a^ p.Glu713Lys	118 PD 193 Controls	C: 44 vs. 46 T: 56 vs. 54 CC: 35 vs. 30 CT: 43 vs. 48 TT: 22 vs. 22	*0.62 0.63*	0.91 (0.65–1.26), *p 0.58*	Romero-Gutierrez et al., 2021
*SLC2A13*	rs1994090^b^ G → T Intronic SNP	118 PD 193 Controls	G: 14 vs. 12 T: 86 vs. 88 TT: 73 vs. 78 TG: 25.8 vs. 21 GG: 1 vs. 1	0.62 0.58	1.16 (0.71–1.88), *p 0.55*	Romero-Gutierrez et al., 2021
*MAPT*	rs242562 G → A Intronic SNP	118 PD 193 Controls	G: 32 vs. 39 A: 68 vs. 67 AA: 43 vs. 35 AG: 48 vs. 52 GG: 9 vs. 13	0.12 0.29	0.75 (0.53–1.06), *p 0.11*	Romero-Gutierrez et al., 2021
*RAIL/ SREBF1*	rs11868035 G → A Intronic SNP	118 PD 193 Controls	G: 47 vs. 41 A: 53 vs. 59 AA: 31 vs. 37 AG: 44 vs. 43 GG: 25 vs. 20	0.16 0.39	1.29 (0.92–1.81), *p 0.13*	Romero-Gutierrez et al., 2021
*SNCA*	rs356219 A → G Intronic SNP	88 PD 88 Controls	A: 36 vs. 49 G: 64 vs. 51 AA: 13.7 vs. 20.5 AG: 44.3 vs. 56.8 GG: 42 vs. 22.7	0.006 0.023	1.80 (1.14–2.83) ***p** **0.011*** AA vs. AG: 1.21 (0.51–2.89), *p* 0.66 AA vs.GG: 2.87 (1.14–7.25), *p* 0.025 DOM: 1.68 (0.78–3.78), *p* 0.210 REC: 2.49 (1.29–4.80), ***p 0.006***	Salas-Leal et al., 2021

OR, Odds ratio; 95% IC, 95% confidence interval; Ref, Reference; IPD, Idiopathic Parkinson's disease; ADPD, Autosomic Dominant Parkinson's disease; EOPD, Early-Onset Parkinson's disease; CNVs, Copy number variations; DOM, Dominant model; REC, Recessive model, HeM, Heterozygote model; DELINS, Deletion-Insertion; INDELS, Insertion–deletion polymorphism; Delx, Exonic Deletions; Cong Imp, cognitive impairment; Mf, Motor Fluctuations; MAF, minor allele frequency; sPD, sporadic Parkinson's disease; NR, No reported; ND, No detected; NA, Not applicated.

^a^Complementary strand. ^b^Indirect marker of the risk variant in LRRK2 p.Gly2385Arg.

Chronological ordering is retained intentionally to reflect the development of genetic evidence and analytical methods over time, consistent with PRISMA guidance for historical tracking of study progression.

Statistically significant values are shown in bold. Italicized *p*-values represent exact values (*p* =), while < and >; indicate values below or above the specified threshold, respectively.

Early-onset PD studies revealed particularly strong contributions from *PRKN*. Biallelic pathogenic variants and exon rearrangements involving exons 2–3, 4, 6, 9, and 11 appeared in 17–54% of early-onset PD cases and were largely absent in controls (Martinez et al., 2004; Guerrero Camacho et al., 2012). Digenic *PRKN–PINK1* and *PRKN–LRRK2* combinations were reported in a minority of patients (Monroy-Jaramillo et al., 2014), underscoring the relevance of mitochondrial quality-control mechanisms.

Within *GBA1*, the p.L444P (rs421016) variant showed a consistently increased risk (Gonzalez-Del Rincon Mde et al., 2013), whereas p.N370S was absent, consistent with its population-specific frequencies globally. Several non-coding *SNCA* polymorphisms—rs3857059, rs356220, rs356203, rs2736990, rs7684318, and rs356219—were associated with PD across independent Mexican cohorts (Dávila-Ortiz de Motellano et al., 2012; Garcia et al., 2016; Salas-Leal et al., 2021). Missense variants p.A53T, p.A30P, and p.E46K were uniformly absent, consistent with their rarity outside defined European and familial clusters.

Across the included literature, the canonical *LRRK2* p.G2019S mutation was consistently absent or extremely rare in Mexican cohorts, whereas alternative non-coding *LRRK2* variants, including rs1491942, showed moderate associations with PD in studies incorporating ancestry-informed analyses (Romero-Gutierrez et al., 2021). Variants in the one-carbon metabolism pathway, particularly *MTHFR* c.677C>T (rs1801133), were associated with PD risk in Mexican populations, with evidence suggesting that effect estimates may vary according to underlying ancestry proportions (Garcia et al., 2015; Romero-Gutierrez et al., 2021).

Variation in *APOE* was associated with PD risk in Mexican cohorts, with risk-modifying effects reported for the ε4 allele and protective associations observed for the ε3 allele (Lopez et al., 2007; Gallegos-Arreola et al., 2009). Associations involving *SYT11* variants—including rs34372695, rs729022, and rs822508—were reported in relation to synaptic vesicle–related pathways implicated in PD (Sesar et al., 2016). Protective signals were also described for *NR4A2* haplotypes, reflecting differential effects across haplotype configurations, as well as for the *ALDH1A1* rs3764435 variant, consistent with roles in dopaminergic regulation and oxidative stress metabolism (Ruiz-Sanchez et al., 2017; Salas-Leal et al., 2019).

### 3.3 Genetic risk classification

Genetic variants were classified into four mutually exclusive categories—pathogenic or likely pathogenic, risk-modifying, protective, and not associated—based on the strength and consistency of evidence reported across the included literature (Table 2). This classification framework was applied uniformly across the main manuscript and Supplementary material to facilitate comparison across heterogeneous study designs and analytical approaches.

**Table 2:** Classification of Parkinson's disease–associated genetic variants identified in Mexican cohorts.

**Category**	**Gene**	**Variant/alteration**	**Evidence basis**	**Primary reference(s)**
Pathogenic/likely pathogenic	*PRKN*	Biallelic pathogenic variants	Early-onset PD; loss-of-function	Martinez et al., 2004; Guerrero Camacho et al., 2012
	*PRKN*	Exon rearrangements (CNVs)	Structural disruption; EOPD	Guerrero Camacho et al., 2012
	*PINK1*	Biallelic pathogenic variants	Recessive EOPD	Monroy-Jaramillo et al., 2014
Risk-modifying	*APOE*	ε4	Increased PD risk	Lopez et al., 2007; Gallegos-Arreola et al., 2009
	*GBA1*	p.L444P (rs421016)	Lysosomal pathway	Gonzalez-Del Rincon Mde et al., 2013
	*LRRK2*	rs1491942	Regulatory; ancestry-aware signal	Romero-Gutierrez et al., 2021
	*MTHFR*	rs1801133 (c.677C>T)	One-carbon metabolism	Garcia et al., 2015; Romero-Gutierrez et al., 2021
	*SNCA*	rs3857059, rs356203, rs356220, rs356219, rs2736990, rs7684318	Regulatory variation	Dávila-Ortiz de Motellano et al., 2012; Garcia et al., 2016; Salas-Leal et al., 2021
	*SYT11*	rs34372695, rs729022, rs822508	Synaptic vesicle pathway	Sesar et al., 2016
Protective	*ALDH1A1*	rs3764435	Oxidative stress metabolism	Salas-Leal et al., 2019
	*APOE*	ε3	Reduced frequency in PD vs. controls	Lopez et al., 2007; Gallegos-Arreola et al., 2009
	*NR4A2*	H1 haplotype	Dopaminergic differentiation	Ruiz-Sanchez et al., 2017
Not associated^*^	*MAPT*	Common haplotypes	No PD association	Multiple studies
	*DRD3*	rs6280	No PD association	Cervantes-Arriaga et al., 2015
	*PRKN*	Isolated heterozygous missense variants	Inconsistent/null	Multiple studies

Variants classified as “Not associated” showed no statistically supported association with Parkinson's disease in Mexican cohorts and are reported here for completeness. Detailed variant-level results, including negative findings, are provided in Supplementary Table 1.

Variants with deterministic effects were confined to biallelic pathogenic alterations in *PRKN* or *PINK1*, recurrent *PRKN* exon rearrangements, and a limited number of reported digenic combinations (Martinez et al., 2004; Guerrero Camacho et al., 2012; Monroy-Jaramillo et al., 2014). None of the included studies incorporated longitudinal follow-up or formal familial segregation analyses; accordingly, penetrance estimates were not derived directly from the reviewed data. Interpretive context for deterministic classification, therefore, relied on established external resources, including ClinVar, OMIM, and consensus frameworks for monogenic PD.

Risk-modifying variants encompassed *GBA1* p.L444P (Gonzalez-Del Rincon Mde et al., 2013), *APOE* ε4 (Lopez et al., 2007; Gallegos-Arreola et al., 2009), *MTHFR* rs1801133 (Garcia et al., 2015; Romero-Gutierrez et al., 2021), regulatory *SNCA* polymorphisms (Dávila-Ortiz de Motellano et al., 2012; Garcia et al., 2016; Salas-Leal et al., 2021), *LRRK2* rs1491942 (Romero-Gutierrez et al., 2021), and *SYT11* variants implicated in synaptic vesicle biology (Sesar et al., 2016). Protective associations were reported primarily for *ALDH1A1* rs3764435 (Salas-Leal et al., 2019), *NR4A2* haplotypes (Ruiz-Sanchez et al., 2017) and the *APOE* ε3 allele (Lopez et al., 2007; Gallegos-Arreola et al., 2009), consistent with their putative roles in dopaminergic maintenance, oxidative stress regulation, and lipid-mediated neuroprotection. Variants evaluated in genes such as *MAPT* and *GSK3B*, as well as isolated *PRKN* missense substitutions, did not show evidence of association with PD in Mexican cohorts and are reported separately in the Supplementary dataset.

### 3.4 Functional and pathway-level integration

**Figure 3:**
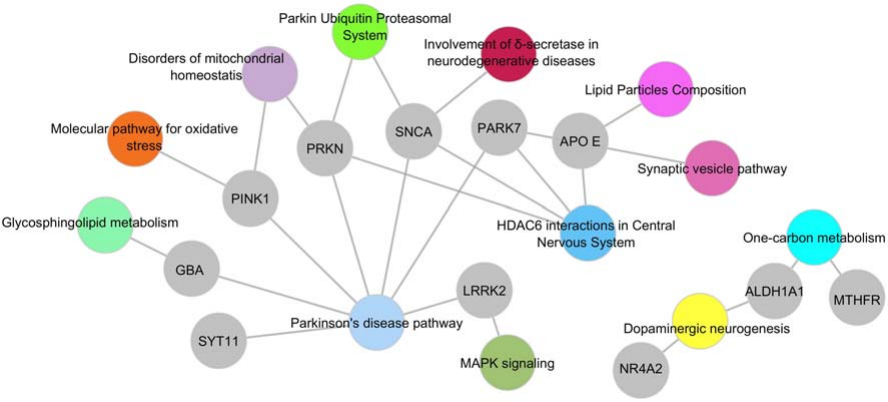
Gene–pathway interaction network of Parkinson's disease–associated genes identified in Mexican cohorts. This network depicts functional relationships between genes reported in 25 Parkinson's disease (PD) studies conducted in 24 PD studies conducted in Mexico and the molecular pathways to which they are assigned. Gray nodes represent genes with reported PD-associated variants, while colored nodes correspond to curated biological processes derived from WikiPathways release 2025.1 using HGNC-standard nomenclature. Pathway categories include oxidative stress responses (orange), mitochondrial homeostasis (purple), glycosphingolipid metabolism (light green), proteasomal and ubiquitin-mediated degradation (green), dopaminergic neurogenesis (yellow), one-carbon metabolism (cyan), lipid particle composition and synaptic vesicle cycling (pink), MAPK signaling (dark green), δ-secretase–related neurodegenerative pathways (red), and HDAC6-mediated interactions in the central nervous system (blue). Edges denote established gene–pathway annotations based on curated functional databases and prior experimental studies; they do not imply direct causal relationships within the Mexican cohorts. The network was generated in Cytoscape v3.10.0 using a force-directed layout optimized for topological clarity. This systems-level representation illustrates convergence across mitochondrial quality control, lysosomal-autophagic recycling, synaptic regulation, and dopaminergic signaling—key functional axes implicated across the genes identified in Mexican PD genetics.

**Figure 4:**
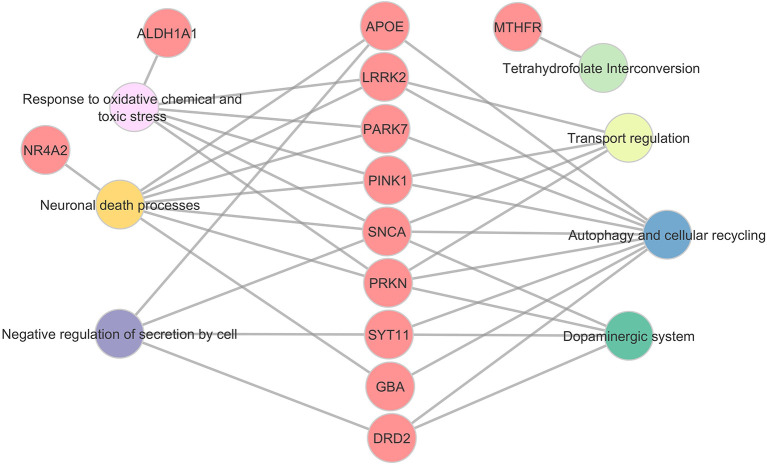
Gene–biological process interaction map of Parkinson's disease–associated loci in Mexican populations. This bipartite network integrates gene-level associations (red nodes) with higher-order biological processes (colored nodes) curated from Gene Ontology: Biological Process (GO:BP, February 2025 release). Genes were selected based on reproducible associations with PD variants, haplotypes, or structural rearrangements (Tables 2–4). Process categories were consolidated at GO levels 3–4 to maintain mechanistic breadth without redundancy, including oxidative stress response (pink), neuronal death pathways (yellow), dopaminergic system signaling (teal), autophagy and cellular recycling (blue), secretion regulation (purple), transport regulation (light green), and folate metabolism (light green; tetrahydrofolate interconversion). Edges represent curated gene-to-process relationships supported by peer-reviewed experimental or association data. The network was generated in Cytoscape v3.10.0 and manually refined to enhance clarity of convergent and divergent mechanisms. Genes are centrally positioned to emphasize multi-process connectivity, whereas peripheral processes delineate the biological domains underlying PD susceptibility in admixed Mexican populations.

To place the observed genetic associations within a broader biological context, implicated variants were mapped onto curated functional frameworks derived from Gene Ontology (Biological Process), KEGG, and WikiPathways, with network reconstruction performed using Cytoscape (Table 3; Figures 3, 4). Pathway assignment was informed by a composite evaluation incorporating three dimensions: the pattern and consistency of genetic evidence reported across studies; functional or mechanistic support from curated pathway annotations and experimental literature; and methodological quality, as assessed by Q-Genie domain scores.

**Table 3:** Biological processes and supporting evidence for Parkinson's disease–associated genes in Mexican populations.

**Biological process/ functional domain**	**Principal gene(s)**	Evidence strength^*^	**Supporting evidence in Mexican cohorts**
Autophagy–lysosomal system and proteostasis	*PRKN, PINK1, LRRK2, GBA1, SNCA, SYT11, APOE*	Strong (*PRKN, GBA1, SNCA*); Moderate–Strong (*PINK1, LRRK2*); Moderate (*SYT11, APOE*)	*PRKN*/*PINK1* mitophagy axis implicated in early-onset PD; *GBA1* p.L444P associated with PD risk; regulatory *SNCA* variants and *LRRK2* rs1491942 linked to endolysosomal pathways
Dopaminergic neurotransmission and synaptic regulation	*SNCA, SYT11, NR4A2, DRD2*	Strong (*SNCA*); Moderate–Strong (*SYT11*); Moderate (*NR4A2, DRD2*)	Multiple intronic *SNCA* polymorphisms associated with PD; significant *SYT11* variants involved in synaptic vesicle cycling; *NR4A2* haplotypes show protective effects consistent with dopaminergic regulation
Neuronal survival and mitochondrial integrity	*PRKN, PINK1, LRRK2, GBA1, SNCA, NR4A2, APOE*	Strong (*PRKN, GBA1, SNCA*); Moderate–Strong (*PINK1, LRRK2*); Moderate (*NR4A2, APOE*)	Risk-modifying and pathogenic variants converge on mitochondrial maintenance, neuronal survival, and stress-response pathways
One-carbon metabolism and methylation pathways	*MTHFR*	Moderate–Strong	*MTHFR* c.677C>T (rs1801133) associated with PD risk; effect magnitude varies across Native American ancestry strata
Oxidative stress response and aldehyde detoxification	*PRKN, PINK1, PARK7, ALDH1A1, LRRK2, SNCA*	Strong (*PRKN, SNCA*); Moderate–Strong (*PINK1, PARK7*); Moderate (*ALDH1A1, LRRK2*)	Protective association for *ALDH1A1* rs3764435; enrichment of *PRKN* CNVs in early-onset PD; pathways consistent with dopaminergic oxidative stress regulation
Regulation of secretion and synaptic homeostasis	*SNCA, SYT11, DRD2, APOE*	Strong (*SNCA*); Moderate–Strong (*SYT11*); Moderate (*DRD2, APOE*)	Enrichment of *SNCA* regulatory variants; synaptic and secretory pathway involvement across cohorts
Vesicular trafficking and membrane dynamics	*SNCA, SYT11, LRRK2, DRD2*	Strong (*SNCA*); Moderate–Strong (*SYT11*); Moderate (*LRRK2, DRD2*)	Regulatory *SNCA* variants and *SYT11* associations implicate vesicle recycling and membrane dynamics

GO:BP, Gene Ontology Biological Process; CNV, copy-number variant; SNV, single-nucleotide variant; PD, Parkinson's disease.

This table summarizes biological processes represented by genes that show statistically significant associations with Parkinson's disease in Mexican cohorts. Functional annotations were derived from Gene Ontology: Biological Process, KEGG, and WikiPathways, and integrated using Cytoscape v3.10. Evidence strength reflects convergence across three independent domains: (i) genetic evidence, including consistency of reported associations and the presence of monogenic pathogenic variants; (ii) functional and mechanistic support from curated pathway databases and experimental literature; and (iii) study quality, assessed using Q-Genie domain scores (Supplementary Table 2). Evidence-strength categories are defined as follows: Strong, robust support across all three domains, including ≥2 independent high-quality studies and/or monogenic pathogenicity; Moderate–Strong, strong support in at least two domains with partial evidence in the third; Moderate, support in a single domain with plausible but incomplete corroboration from additional domains. No genes in this table met the criteria for a weak-evidence classification. This table reflects biological convergence rather than causal inference; variant-level data are reported in Table 1, and study-level characteristics are provided in Supplementary Table 2.

Within this integrative framework, *PRKN, PINK1, SNCA*, and *GBA1* emerged as central nodes, reflecting convergence across multiple lines of evidence. Network visualization delineated three major functional modules: mitochondrial quality control centered on the *PRKN–PINK1* axis; lysosomal and autophagy-related processes involving *GBA1, SYT11*, and associated effectors; and synaptic and dopaminergic pathways encompassing *SNCA, NR4A2*, and *DRD2*. Process-level mapping further highlighted enrichment in pathways related to oxidative and toxic stress responses, dopaminergic system regulation, autophagy and cellular recycling, modulation of neuronal survival, and negative regulation of secretion (Figure 4).

**Table 4:** Cross-population comparison of Parkinson's disease–associated genetic variants.

**Gene/variant**	**Population(s)**	**Allele frequency (MAF%)**	**Effect estimate (OR [95% CI])**	**Direction of effect**	**Functional interpretation**	**Reference(s)**
*ALDH1A1* rs3764435	Mexico/Latin Am./Europe/Asia	57/54/49/52	0.40 [0.19–0.84]/0.73 [0.52–1.03]/—/—	↓ Protective	Aldehyde detoxification and oxidative stress regulation	Salas-Leal et al., 2019
*APOE* ε4	Mexico/Latin Am./Europe/Asia	13/14/12/9	1.7 [1.1–2.6]/1.6 [1.2–2.1]/1.5 [1.2–1.9]/1.3 [1.0–1.6]	↑ Risk	Lipid transport and proteostasis pathways implicated in neurodegeneration	Lopez et al., 2007; Gallegos-Arreola et al., 2009
*GBA1* rs421016 (p.L444P)	Mexico/Latin Am./Europe/Asia	2.8/3.1/1.4/0.6	3.9 [1.7–8.8]/3.4 [1.8–6.6]/2.2 [1.4–3.3]/1.9 [1.2–3.0]	↑ Risk	Lysosomal glucocerebrosidase deficiency linked to α-synuclein accumulation	Gonzalez-Del Rincon Mde et al., 2013; Zhang et al., 2018
*LRRK2* p.G2019S	Mexico/Latin Am./Europe/North Africa/Asia	—/0.5/2.6/4.2/1.3	—/—/9.1 [6.7–12.3]/11.0 [7.9–15.5]/5.6 [3.4–9.3]	↑ Risk	Marked population differences in allele frequency	Yescas et al., 2010; Nalls et al., 2019
*LRRK2* rs1491942 (intronic)	Mexico/Europe/Asia	59/52/46	1.7 [1.2–2.4]/1.3 [1.1–1.6]/1.2 [1.0–1.5]	↑ Risk	Regulatory variant reported in admixed and non-European cohorts	Romero-Gutierrez et al., 2021; Park et al., 2023
*MTHFR* rs1801133 (C677T)	Mexico/Latin Am./Europe/Asia	58/55/45/43	1.5 [1.1–2.2]/1.4 [1.1–1.8]/1.3 [1.0–1.6]/1.2 [0.9–1.4]	↑ Risk	One-carbon metabolism and oxidative stress pathways	Garcia et al., 2015; Romero-Gutierrez et al., 2021
*NR4A2* haplotypes (rs34884856–rs35479735)	Mexico/Latin Am./Europe/Asia	54/48/46/44	0.67 [0.46–0.97]/0.81 [0.61–1.07]/—/—	↓ Protective	Transcriptional regulation of dopaminergic genes	Ruiz-Sanchez et al., 2017
*PINK1* (point mutations)	Mexico/Europe/Asia	0.7/0.5/0.8	≈5.2 [1.9–14.3]/4.8 [1.5–12.6]/6.1 [2.1–17.4]	↑ Risk	Recessive variants affecting mitochondrial stress pathways	Monroy-Jaramillo et al., 2014
*PRKN* (CNVs/exon 2–11 deletions)	Mexico (EOPD)/Europe/Asia	1.8/1.0/1.3	≈10.0 [3.1–32.5]/6.7 [2.4–18.3]/5.4 [1.9–15.0]	↑ Risk (monogenic)	Loss-of-function CNVs associated with impaired mitophagy	Martinez et al., 2004; Guerrero Camacho et al., 2012; Monroy-Jaramillo et al., 2014
*SNCA* rs356220	Mexico/Latin Am./Europe/Asia	48/42/36/40	2.1 [1.4–3.3]/1.8 [1.3–2.6]/1.5 [1.3–1.7]/1.4 [1.2–1.6]	↑ Risk	Regulatory enhancer variant replicated across populations	Davila-Ortiz de Montellano et al., 2016; Garcia et al., 2016
*SNCA* rs7684318	Mexico/Latin Am./Europe/Asia	69/62/48/51	9.9 [5.9–16.5]/3.1 [1.8–5.4]/1.6 [1.3–1.9]/1.8 [1.4–2.2]	↑ Risk	Regulatory variant associated with increased α-synuclein expression	Naushad et al., 2021; Salas-Leal et al., 2021
*SYT11* rs34372695	Mexico/Latin Am./Europe/Asia	45/42/37/40	2.1 [1.2–3.9]/1.8 [1.1–3.0]/1.3 [1.0–1.6]/1.2 [0.9–1.4]	↑ Risk	Synaptic vesicle cycling and dopaminergic release	Sesar et al., 2016

This table summarizes cross-population allele frequencies, effect sizes, and functional implications for major Parkinson's disease–associated variants reported in Mexican cohorts (2004–2025) and comparative global studies. Methodological integration: (a) Allelic data standardized to odds ratios (95 % CIs) using dominant/additive models. (b) Mexican data quality-scored with Q-Genie; non-replicable low-quality items excluded from meta-comparison. (c) References prioritized from replicated, peer-reviewed studies; allele frequencies harmonized using dbSNP build 155. (d) Direction of effect: ↑ = risk allele; ↓ = protective allele; — = not detected or not estimable. These findings reveal that Mexican and broader Latin American populations exhibit stronger effects for regulatory SNCA and APOE alleles, the absence of LRRK2 G2019S, and distinct protective NR4A2 and ALDH1A1 variants, underscoring ancestry-dependent mechanisms and the need for region-specific genetic panels.

### 3.5 Cross-population comparisons

Variants that were absent or detected at very low frequencies in Mexican cohorts—most notably *LRRK2* p.G2019S and *GBA1* p.N370S—were evaluated in the context of global allele-frequency distributions and study design characteristics rather than interpreted as evidence of population-specific depletion (Table 4). Such patterns are compatible with sampling variation, limited cohort sizes, and known differences in ancestral background relative to predominantly European reference populations.

Similarly, differences in reported effect sizes across populations, including those observed for regulatory *SNCA* variants such as rs7684318, were considered in light of study-level heterogeneity, including sample size, case–control matching, and analytical covariate adjustment. Among the included studies, only (Romero-Gutierrez et al. 2021) incorporated formal global and local ancestry modeling, reporting modulation of the association between *MTHFR* c.677C>T (rs1801133) and PD risk across Native American ancestry strata. These observations are presented descriptively, and their biological and epidemiological implications are addressed in the Discussion.

## 4 Discussion

### 4.1 Core genetic signals and mechanistic insights

Across the 24 studies included in this review, a coherent set of genetic signals emerges that aligns with international PD genetics while revealing features specific to Mexican cohorts. The most robust associations converge on biological systems central to PD pathogenesis, including mitochondrial quality control, lysosomal–autophagic function, proteostasis, oxidative stress responses, and dopaminergic maintenance. The high burden of *PRKN* exon rearrangements among individuals with early-onset PD (Martinez et al., 2004; Guerrero Camacho et al., 2012) underscores the primacy of the *PRKN–PINK1* axis in recessive PD. Likewise, associations involving regulatory *SNCA* variation replicate broader evidence that modulation of *SNCA* expression rather than coding mutations drives susceptibility in most populations.

The *GBA1* p.L444P signal observed in Mexican studies (Gonzalez-Del Rincon Mde et al., 2013) aligns with its established role in PD risk through lysosomal dysfunction. Reported protective associations involving *ALDH1A1* rs3764435 and *NR4A2* haplotypes (Ruiz-Sanchez et al., 2017; Salas-Leal et al., 2019) are biologically plausible given the known roles of these genes in dopaminergic homeostasis; nevertheless, these findings derive from single-site candidate-gene studies and require cautious interpretation. Importantly, none of the included studies provide ancestry-aware evidence supporting differential resilience or variant modulation. These observations, therefore, offer biological context but remain preliminary and await confirmation in larger, systematically powered, ancestry-informed cohorts.

Although the core findings across Mexican studies align with international PD biology, methodological constraints—including modest sample sizes, limited confounder adjustment, incomplete genotyping quality control, and heterogeneous reporting—necessitate conservative interpretation. Nevertheless, the functional network frameworks presented in Figures 3, 4 underscore how these molecular findings converge on well-established pathogenic processes, providing a coherent biological narrative across otherwise diverse candidate-gene studies (Table 3).

### 4.2 Integrating risk categories and interpretive constraints

To standardize interpretation across a heterogeneous body of literature, all variants were classified into four mutually exclusive categories: pathogenic or likely pathogenic, risk-modifying, protective, or not associated (Table 2). Deterministic variants were limited to biallelic *PRKN* and *PINK1* mutations, *PRKN* exon-level rearrangements, and documented digenic combinations. Crucially, we did not derive penetrance estimates from the included studies, as none incorporated the familial or longitudinal designs needed to support such inference; instead, penetrance categories rely strictly on authoritative external sources such as ClinVar, OMIM, and consensus PD genetics reviews.

Most variant associations fall within the risk-modifying class, including *GBA1* p.L444P, *APOE* ε4, *MTHFR* rs1801133, *SYT11* rs34372695, non-coding *SNCA* polymorphisms, and *LRRK2* rs1491942. Several estimated effect sizes—particularly for *SNCA* rs7684318—exceed those reported in large international cohorts. As now explicitly acknowledged, these apparent discrepancies may reflect small-sample inflation, incomplete adjustment for population structure, or stochastic variance rather than genuine population-level divergence. Protective associations involving *ALDH1A1* and *NR4A2* further reinforce the relevance of oxidative stress metabolism and dopaminergic transcriptional identity to PD risk modulation.

### 4.3 Population structure, cross-population contrasts, and the relevance of Indigenous genomic diversity

Interpretation of genetic associations across populations requires a balanced consideration of statistical limitations and biological context. Although this review highlights well-recognized constraints of candidate-gene studies—particularly modest sample sizes, heterogeneous analytical strategies, and incomplete adjustment for confounding—these limitations alone do not preclude the contribution of population-specific factors or functionally divergent effects. At present, the available evidence does not allow these possibilities to be disentangled conclusively. Consequently, apparent inflation of effect sizes or the absence of well-established variants should be interpreted as compatible with multiple, non-exclusive explanations, including sampling variance, methodological constraints, and underlying population structure.

Among the studies included, only Romero-Gutierrez et al. (2021) incorporated global ancestry proportions, demonstrating modulation of the association between *MTHFR* c.677C>T (rs1801133) and PD risk across Native American ancestry strata; however, the study did not perform formal local-ancestry inference. This finding remains the sole ancestry-informed association within the current Mexican PD genetics literature. Broader cross-population contrasts, therefore, warrant circumspect interpretation. For example, the low frequency or apparent absence of variants such as *LRRK2* p.G2019S or *GBA1* p.N370S in Mexican cohorts may reflect regional allele-frequency distributions, stochastic sampling effects, and limited statistical power. At the same time, given the distinct admixture profiles and fine-scale population structure characteristic of Mexico, these patterns cannot be attributed solely to methodological factors, and population-modulated biological effects cannot be ruled out.

Similarly, comparatively large effect estimates reported for regulatory variants in *SNCA* and other loci may be influenced by the winner's curse and the design constraints of candidate-gene analyses. However, differences in linkage disequilibrium structure, regulatory architecture, or genetic background across populations could plausibly contribute to variation in observed effect sizes. Evidence from polygenic risk score analyses further illustrates this complexity: models derived from European datasets show reduced portability in admixed Latino populations, often yielding attenuated risk estimates (Loesch et al., 2022). Nevertheless, these models retain measurable predictive value when recalibrated and validated in Latino cohorts, underscoring the importance of ancestry-aware modeling rather than categorical dismissal of cross-population relevance.

Genomic anthropological studies provide important context for interpreting these findings. Garcia et al. (2023) demonstrated that Indigenous populations from southern Mexico exhibit distinctive genomic architectures shaped by long-term demographic history and signatures of positive selection, particularly within immune and inflammatory pathways (Garcia et al., 2023). Although not focused on PD, this work illustrates the depth of population-specific structure present within Mexico and highlights factors that may influence allele-frequency distributions, linkage disequilibrium patterns, and genetic backgrounds relevant to association testing. In this context, differences observed between Mexican cohorts and external reference populations should not be interpreted in isolation from the complex demographic and evolutionary histories underlying Indigenous and admixed populations.

Taken together, these observations highlight a major limitation of the current literature: the scarcity of ancestry-aware analyses in PD genetics despite clear evidence of genomic heterogeneity. Addressing this gap will require larger, well-powered studies that incorporate harmonized phenotyping, systematic global and local ancestry inference, and the deliberate inclusion of Indigenous and other underrepresented populations. Such efforts are essential to refine effect-size estimates, improve reproducibility, and determine whether population-specific or functionally divergent mechanisms contribute meaningfully to PD risk in Mexico and across Latin America.

## 5 Conclusions

This systematic review synthesizes 20 years of genetic studies of PD in Mexico and reveals convergent biological themes involving mitochondrial maintenance, lysosomal–autophagic activity, dopaminergic regulation, and oxidative stress responses. The findings also reinforce PRKN's consistent contribution to early-onset PD and demonstrate risk-modifying roles for *GBA1* p.L444P, *APOE* ε4, *MTHFR* rs1801133, regulatory *SNCA* variation, and other loci.

However, the evidence base remains insufficient to support ancestry-informed conclusions regarding resilience, variable penetrance, or therapeutic responsiveness. Only one included study applied ancestry-aware modeling, using a restricted marker set to estimate global ancestry proportions, without local-ancestry deconvolution (Romero-Gutierrez et al., 2021). Moreover, recent genomic studies, such as Garcia et al. (2023), underscore the complex demographic and selective histories that characterize Indigenous populations in Mexico, further highlighting the limitations of interpreting PD genetic associations without comprehensive ancestry analysis.

Future progress will require large-scale, well-powered studies that integrate global and local ancestry deconvolution, genome-wide discovery approaches, harmonized phenotyping, and replication across diverse Latino populations. Such efforts are essential not only for accurately characterizing the genetic architecture of PD in Mexican and Indigenous populations but also for ensuring the equitable development of risk models and therapeutic strategies in the genomics era.
