# Supplementary material for: The genetic architecture of Parkinson's disease in Mexico: a systematic review
Source: Front Aging Neurosci. 2026 Feb 19;18:1709246. doi: 10.3389/fnagi.2026.1709246 (PMC12960540; doi:10.3389/fnagi.2026.1709246)
Supplement: Supplementary file 1 [file Table_1.docx]

***Supplementary Table 1.*** Q-Genie Quality Scores of Included Studies

| Studies | Items | | | | | | | | | | | Total score | Quality classification |
| --- | --- | --- | --- | --- | --- | --- | --- | --- | --- | --- | --- | --- | --- |
|  | **1** | **2** | **3** | **4** | **5** | **6** | **7** | **8** | **9** | **10** | **11** |  |  |
| Martínez et al., 2004 | 6 | 5 | 3 | 5 | 3 | 3 | 2 | 2 | 1 | 2 | 4 | 36 | Moderate Quality |
| Ramírez-Jirano et al., 2006 | 6 | 5 | 3 | 3 | 2 | 2 | 2 | 2 | 3 | 1 | 5 | 34 | Poor Quality |
| López et al., 2007 | 6 | 6 | 6 | 5 | 4 | 5 | 5 | 6 | 7 | 6 | 7 | 63 | Good Quality |
| Ramírez-Jirano et al., 2007 | 4 | 5 | 3 | 5 | 4 | 1 | 2 | 3 | 3 | 1 | 3 | 34 | Poor Quality |
| Gallegos-Arreola et al., 2009 | 6 | 5 | 3 | 4 | 3 | 6 | 5 | 6 | 7 | 6 | 5 | 56 | Good Quality |
| Yescas et al., 2010 | 6 | 6 | 6 | 5 | 5 | 4 | 5 | 5 | 4 | 6 | 7 | 59 | Good Quality |
| Martínez et al., 2010 | 6 | 6 | 5 | 5 | 5 | 4 | 4 | 3 | 2 | 5 | 5 | 50 | Good Quality |
| Dávila-Ortiz de Motellano et al., 2010 | 4 | 5 | 0 | 3 | 4 | 3 | 1 | 3 | 1 | 3 | 5 | 32 | Moderate Quality |
| Guerrero et al., 2012 | 7 | 7 | 6 | 7 | 5 | 6 | 5 | 6 | 7 | 6 | 7 | 69 | Good Quality |
| Gonzalez del Rincon et al., 2013 | 7 | 7 | 7 | 6 | 3 | 6 | 5 | 5 | 6 | 4 | 7 | 63 | Good Quality |
| Monrroy -Jaramillo et al., 2014 | 7 | 6 | 6 | 6 | 3 | 6 | 5 | 6 | 6 | 6 | 7 | 64 | Good Quality |
| García et al., 2014 | 7 | 6 | 5 | 6 | 4 | 5 | 7 | 6 | 5 | 5 | 6 | 62 | Good Quality |
| Cervantes-Arriaga et al., 2015 | 7 | 6 | 5 | 4 | 3 | 5 | 4 | 6 | 6 | 5 | 5 | 56 | Good Quality |
| García et al., 2015 | 6 | 6 | 6 | 6 | 3 | 6 | 5 | 6 | 6 | 5 | 6 | 61 | Good Quality |
| García et al., 2016 | 6 | 6 | 5 | 5 | 3 | 4 | 5 | 5 | 5 | 5 | 6 | 55 | Good Quality |
| Dávila-Ortiz de Motellano et al., 2016 | 6 | 6 | 6 | 5 | 3 | 5 | 6 | 6 | 5 | 5 | 6 | 59 | Good Quality |
| Sesar et al., 2016 | 6 | 6 | 5 | 7 | 3 | 6 | 6 | 7 | 6 | 7 | 6 | 65 | Good Quality |
| García et al., 2017 | 6 | 6 | 5 | 5 | 3 | 6 | 4 | 5 | 4 | 4 | 4 | 52 | Good Quality |
| Ruiz- Sanchéz et al., 2017 | 7 | 6 | 7 | 7 | 3 | 6 | 6 | 7 | 7 | 7 | 6 | 69 | Good Quality |
| García et al., 2019 | 6 | 6 | 5 | 6 | 4 | 4 | 4 | 3 | 3 | 4 | 6 | 51 | Good Quality |
| Miranda-Moráles et al., 2019 | 6 | 6 | 4 | 6 | 3 | 5 | 6 | 5 | 5 | 4 | 6 | 56 | Good Quality |
| Salas-Leal et al., 2019 | 6 | 6 | 4 | 6 | 3 | 6 | 6 | 6 | 5 | 5 | 5 | 58 | Good Quality |
| Romero-Gutierrez et al., 2021 | 7 | 6 | 6 | 7 | 3 | 6 | 5 | 7 | 7 | 7 | 7 | 68 | Good Quality |
| Salas-Leal et al., 2021 | 6 | 6 | 4 | 6 | 3 | 6 | 6 | 6 | 6 | 6 | 6 | 61 | Good Quality |

Each Q-Genie item is scored from 1 (poor) to 7 (excellent). The 11 items are: (1) Rationale for study; (2) Selection and definition of outcome; (3) Selection and comparability of comparison groups; (4) Technical classification of exposure; (5) Non-technical classification of exposure; (6) Other sources of bias; (7) Sample size and power; (8) A priori planning of analyses; (9) Statistical methods and control for confounding; (10) Testing of assumptions and inferences; (11) Appropriateness of inferences drawn. Classification thresholds follow Q-Genie guidelines. Studies with control groups: poor quality ≤35; moderate quality >35 and ≤45; good quality >45. Studies without control groups: poor quality ≤32; moderate quality >32 and ≤40; good quality >40.

| **Article title:** | | Detección de polimorfismos en el gen Parkin como biomarcadores predictivos de la enfermedad de Parkinson | | | |
| --- | --- | --- | --- | --- | --- |
| **First Author:** | | Martinez, RH | | | |
| **Year:** | | 2004 | | | |
| **Journal:** | | Revista Mexicana de Neurociencias | | | |
| **Study Design:** | | Case-Control Studies | | □ With control groups | |
|  | | | | | |
| **Item** | **Question** | | **Considerations** | **Score (1-7)** | **Notes/Comments** |
| **1** | **RATIONALE FOR STUDY** | | Was a scientific rationale for chosen genes presented to avoid selective reporting of positive results? If the GWAS design (hypothesis-free approach) was the rationale for selecting this design, was it presented? | **6** | Justification: Provides contextual information that supports investigating variants in the selected gene within the framework of the disorder (Parkinson's disease). Justifies conducting the study in a Mexican population. States the objective of the study. Limitations: Does not present a specific hypothesis. |
| **2** | **SELECTION AND DEFINITION OF OUTCOME** | | Were cases appropriately defined? Were participants appropriately sampled to avoid selection bias? Were case/outcome assessors blinded to genotype status?the If applicable, was follow-u,p lenwas gth appropriate and the attrition rate acceptable? Outcome definitions: independent adjudication/lab measures (strong) vs self-report (moderate) vs no description (poor) | **5** | Justification: Cases (n=50) with Parkinson's disease were evaluated using standardised diagnostic criteria, with additional assessments (psychometric tests, electrophysiological studies, and brain MRI). Participants were from northern Mexico. Limitations: Selection criteria (inclusion/exclusion) are not detailed. Genotype blinding is not mentioned—convenience sample. Specific information on ancestry is not available. |
| **3** | **SELECTION AND COMPARABILITY OF COMPARISON GROUPS** | | Were controls appropriately defined? Were controls sampled to minimize selection bias? Was detailed description of selection procedure outlined? Were assessors of control status blinded to genotype? In multi-ethnic studies, were sub-populations (ethnicity) reported? | **3** | Justification: Age-matched controls were included (n=60). Participants underwent neurological evaluation to rule out neurodegenerative diseases. They were from northern Mexico. Limitations: Selection criteria (inclusion/exclusion) and the percentage of women/men in the group are not specified. Genotype blinding is not mentioned—convenience sample. Specific information on ancestry is not available. |
| **4** | **TECHNICAL CLASSIFICATION OF EXPOSURE** | | Was DNA source and storage method appropriate? Were DNA ascertainment methods similar for comparison groups? Was genotyping platform and allele-calling algorithm appropriate? Were genotyping error and call rates appropriate (≥95%)? Were genotype call rates and SNP missingness similar between groups? Was Hardy-Weinberg equilibrium tested in controls? Did authors check for outlying heterozygosity? If genotypes imputed, were methods described? | **5** | Justification: The DNA extraction and genotyping techniques (PCR and SSCP) are described, demonstrating a methodology valid for the time. It is reported that all patients had the correct 12 exons (100% amplification). Limitations: HW equilibrium verification was not performed on the controls. Error rates are not mentioned. |
| **5** | **NON-TECHNICAL CLASSIFICATION OF EXPOSURE** | | Did blinded assessor conduct genotyping? Was genotyping conducted simultaneously or in batches (same methods)? If applicable, were samples randomized prior to genotyping (not all controls/cases on same plate)? | **3** | Justification: A standardized technique, corroborated twice to confirm positives, was described. Limitations: Blinding is not mentioned, nor is it specified whether genotyping was simultaneous or in batches. Sample randomization is not mentioned. |
| **6** | **OTHER SOURCES OF BIAS** | | Were all sources of bias disclosed and their effect on results discussed? (selection bias, classification bias, time-lag bias, attrition bias, etc.) | **3** | Justification: They acknowledge other unassessed risk factors, such as environmental ones, and partially discuss the study's limitations. Limitations: They do not discuss selection bias (convenience sample), information bias (lack of blinding), selective publication bias, or uncontrolled ancestry bias. |
| **7** | **SAMPLE SIZE AND POWER** | | Was sample size appropriate? Was an a priori power analysis conducted? | **2** | Justification: Sample comparable to other family studies (n=50 cases, 60 controls). Limitations: Convenience sample, no discussion of the statistical power achieved. No formal statistical analysis |
| **8** | **A PRIORI PLANNING OF ANALYSES** | | Was analysis plan appropriate and sufficiently described? Was selective/inappropriate reporting avoided (all test results reported)? Were tested subgroups, interactions, and sensitivity analyses described? Was statistical software identified? | **2** | Justification: No statistical analysis, only a descriptive plan. All analyzed exons are reported (2, 3, 4, 6, 7, 9, 11). |
| **9** | **STATISTICAL METHODS AND CONTROL FOR CONFOUNDING** | | Were important confounders appropriately controlled? Was missing data handled appropriately (<10% missing acceptable)? Were results adjusted for multiple testing? For multi-ethnic studies, did statistical methods (e.g. PCA) control for confounding? | **1** | Justification: Descriptive analysis, without statistical inference. |
| **10** | **TESTING OF ASSUMPTIONS AND INFERENCES** | | Were all assumptions tested? Specifically: i) If haplotypes inferred, was method reported? ii) Were distant relatives/consanguinity tested? iii) Were reported sex and ethnicity checked? | **2** | Justification: They acknowledge that sequencing is needed for confirmation. Limitations: They do not verify familial relationships or consanguinity. They do not verify ethnicity, although they do indicate that the samples are from northern Mexico. |
| **11** | **APPROPRIATENESS OF INFERENCES DRAWN** | | Were conclusions supported by results and appropriate methods? | **4** | Justification: Conclusions are partially adequate. They suggest an association (predisposing genetic factor). They acknowledge limitations and are cautious in their interpretation. Limitations: They cannot establish a causal relationship without statistical analysis. |
|  |  | | **TOTAL SCORE** | **36** | of 77 |
| **FINAL QUALITY RATING:** | | | | **□ Moderate Quality** | |

| **Article title:** | | **Polimorfismo-116C-G del gen a-sinucleína en pacientes con enfermedad de Parkinson** | | | |
| --- | --- | --- | --- | --- | --- |
| **First Author:** | | Ramirez-Jirano, LJ | | | |
| **Year:** | | 2006 | | | |
| **Journal:** | | Salud Publica de México | | | |
| **Study Design:** | | Case-Control Studies | | □ With control groups | |
|  | | | | | |
| **Item** | **Question** | | **Considerations** | **Score (1-7)** | **Notes/Comments** |
| **1** | **RATIONALE FOR STUDY** | | Was a scientific rationale for chosen genes presented to avoid selective reporting of positive results? If GWAS design (hypothesis-free approach), was rationale for selecting this design presented? | **6** | Justification: Provides contextual information that supports investigating variants in the selected gene within the framework of the disorder under investigation (Parkinson's disease). Justifies conducting the study in a Mexican population. States the objective of the study. Limitations: Descriptive objective instead of a hypothesis. Inconsistent previous references. |
| **2** | **SELECTION AND DEFINITION OF OUTCOME** | | Were cases appropriately defined? Were participants appropriately sampled to avoid selection bias? Were case/outcome assessors blinded to genotype status? If applicable, was follow-up length appropriate and attrition rate acceptable? Outcome definitions: independent adjudication/lab measures (strong) vs self-report (moderate) vs no description (poor) | **5** | Justification: Cases evaluated using standardized diagnostic criteria by specialists. Sampling in northwestern Mexico. Limitations: Selection criteria (inclusion/exclusion) are not detailed. Genotype blinding is not mentioned—convenience sample. Specific information on ancestry is not available. |
| **3** | **SELECTION AND COMPARABILITY OF COMPARISON GROUPS** | | Were controls appropriately defined? Were controls sampled to minimize selection bias? Was detailed description of selection procedure outlined? Were assessors of control status blinded to genotype? In multi-ethnic studies, were sub-populations (ethnicity) reported? | **3** | Justification: Controls (n=121) with no history of neurological disease or dementia, from northwestern Mexico. Limitations: Selection criteria (inclusion/exclusion) are not detailed. Genotype blinding is not mentioned. The sample was a convenience sample, unpaired, and lacked ethnic information. Age- and sex-matching information was also unavailable. |
| **4** | **TECHNICAL CLASSIFICATION OF EXPOSURE** | | Was DNA source and storage method appropriate? Were DNA ascertainment methods similar for comparison groups? Was genotyping platform and allele-calling algorithm appropriate? Were genotyping error and call rates appropriate (≥95%)? Were genotype call rates and SNP missingness similar between groups? Was Hardy-Weinberg equilibrium tested in controls? Did authors check for outlying heterozygosity? If genotypes imputed, were methods described? | **3** | Justification: Describes the use of standardized techniques for DNA collection and extraction, as well as genotyping by PCR (without specifying the methodology). They state that analyzing samples in duplicate confirms genotyping. Limitations: They do not mention verifying the hard water balance in the controls. Technical details are missing due to limitations in the letter format. |
| **5** | **NON-TECHNICAL CLASSIFICATION OF EXPOSURE** | | Did blinded assessor conduct genotyping? Was genotyping conducted simultaneously or in batches (same methods)? If applicable, were samples randomized prior to genotyping (not all controls/cases on same plate)? | **2** | Justification: They acknowledge some limitations. Limitations: They do not address selection bias (unmatched volunteer controls), information bias (lack of blinding), age bias, sex bias, or uncontrolled ancestry bias. |
| **6** | **OTHER SOURCES OF BIAS** | | Were all sources of bias disclosed and their effect on results discussed? (selection bias, classification bias, time-lag bias, attrition bias, etc.) | **2** | Justification: They acknowledge some limitations. Limitations: They do not discuss selection bias (unmatched voluntary controls), information bias (lack of blinding), age bias, sex bias, or ethnicity bias. |
| **7** | **SAMPLE SIZE AND POWER** | | Was sample size appropriate? Was an a priori power analysis conducted? | **2** | Justification: Using more controls is a valid strategy to maximize power; however, it does not mention matching by age or sex—limitations: No discussion of the statistical power achieved. |
| **8** | **A PRIORI PLANNING OF ANALYSES** | | Was analysis plan appropriate and sufficiently described? Was selective/inappropriate reporting avoided (all test results reported)? Were tested subgroups, interactions, and sensitivity analyses described? Was statistical software identified? | **2** | Justification: Basic analysis is appropriate but limited. They report frequency and χ² results. Limitations: They do not mention statistical software, Odds ratios, 95% confidence intervals, corrections, or multiple comparisons. |
| **9** | **STATISTICAL METHODS AND CONTROL FOR CONFOUNDING** | | Were important confounders appropriately controlled? Was missing data handled appropriately (<10% missing acceptable)? Were results adjusted for multiple testing? For multi-ethnic studies, did statistical methods (e.g. PCA) control for confounding? | **3** | Justification: Basic analysis. Limitations: Does not analyze important factors such as age, sex, or environmental factors, does not calculate Odds ratios with 95% CI, does not report HW equilibrium in controls |
| **10** | **TESTING OF ASSUMPTIONS AND INFERENCES** | | Were all assumptions tested? Specifically: i) If haplotypes inferred, was method reported? ii) Were distant relatives/consanguinity tested? iii) Were reported sex and ethnicity checked? | **1** | Justification: Basic analysis. Limitations: Does not analyse important factors such as age, sex, or environmental factors; does not calculate Odds ratios with 95% CIs; does not report HW equilibrium in controls. |
| **11** | **APPROPRIATENESS OF INFERENCES DRAWN** | | Were conclusions supported by results and appropriate methods? | **5** | Justification: Appropriate conclusions. They acknowledge the negative result (p>0.05) and are cautious in indicating that these data will be useful in describing these frequencies. They recognize limitations such as sample size and the need to study more genes. They suggest studying more patients, other polymorphisms, and haplotypes. They do not make inappropriate causal claims. Limitations: They lack ancestry data, therefore they cannot verify ethnic variability. |
|  |  | | **TOTAL SCORE** | **34** | of 77 |
| **FINAL QUALITY RATING:** | | | | **□ Poor Quality** | |

| **Article title:** | | Apolipoprotein E ε4 allele is associated with Parkinson disease risk in a Mexican Mestizo population | | | |
| --- | --- | --- | --- | --- | --- |
| **First Author:** | | Lopez, M | | | |
| **Year:** | | 2007 | | | |
| **Journal:** | | Movement Disorders | | | |
| **Study Design:** | | Case-Control Studies | | □ With control groups | |
|  | | | | | |
| **Item** | **Question** | | **Considerations** | **Score (1-7)** | **Notes/Comments** |
| **1** | **RATIONALE FOR STUDY** | | Was a scientific rationale for chosen genes presented to avoid selective reporting of positive results? If GWAS design (hypothesis-free approach), was rationale for selecting this design presented? | **6** | Justification: To provide robust information justifying the selection of the analyzed variants within the framework of the investigated disorder (Parkinson's disease). To justify conducting the study in a Mexican population. To state the research objective. Limitations: The hypothesis is not explicit, but the context implies it. |
| **2** | **SELECTION AND DEFINITION OF OUTCOME** | | Were cases appropriately defined? Were participants appropriately sampled to avoid selection bias? Were case/outcome assessors blinded to genotype status? If applicable, was follow-up length appropriate and attrition rate acceptable? Outcome definitions: independent adjudication/lab measures (strong) vs self-report (moderate) vs no description (poor) | **6** | Justification: Cases (n=229) evaluated using standardized diagnostic criteria. Systematic recruitment in specialized medical units, with family history. Participants with Mexican ancestry across three generations for ethnic homogeneity. Robust sample. Limitations: Genotype blinding is not mentioned—convenience sample. |
| **3** | **SELECTION AND COMPARABILITY OF COMPARISON GROUPS** | | Were controls appropriately defined? Were controls sampled to minimize selection bias? Was detailed description of selection procedure outlined? Were assessors of control status blinded to genotype? In multi-ethnic studies, were sub-populations (ethnicity) reported? | **6** | Justification: Controls were included (n=229) and matched by age and sex. Controls had no neurodegenerative disorder, and their source was described (spouses, companions of patients, or those with other illnesses). Inclusion criteria were described, including Mexican ancestry across three generations for ethnic homogeneity. Limitations: Controls were spouses/companions (not the general population). Genotype blinding is not mentioned—convenience sample. |
| **4** | **TECHNICAL CLASSIFICATION OF EXPOSURE** | | Was DNA source and storage method appropriate? Were DNA ascertainment methods similar for comparison groups? Was genotyping platform and allele-calling algorithm appropriate? Were genotyping error and call rates appropriate (≥95%)? Were genotype call rates and SNP missingness similar between groups? Was Hardy-Weinberg equilibrium tested in controls? Did authors check for outlying heterozygosity? If genotypes imputed, were methods described? | **5** | Justification: Referenced molecular analysis techniques are described. HW equilibrium verification is performed. Detailed frequencies (allelic and genotypic) are reported. Limitations: Genotyping quality controls are not described (error rates, genotyping callouts, SNP loss). |
| **5** | **NON-TECHNICAL CLASSIFICATION OF EXPOSURE** | | Did blinded assessor conduct genotyping? Was genotyping conducted simultaneously or in batches (same methods)? If applicable, were samples randomized prior to genotyping (not all controls/cases on same plate)? | **4** | Justificación: Método estandarizado usado es reproducible. Limitaciones: No menciona cegamiento ni aleatorización. No especifica si genotipado fue simultáneo o en lotes |
| **6** | **OTHER SOURCES OF BIAS** | | Were all sources of bias disclosed and their effect on results discussed? (selection bias, classification bias, time-lag bias, attrition bias, etc.) | **5** | Justificación: Incluye discusión de heterogeneidad poblacional, discuten factores ambientales, reconocen limitación de controles, discuten mezcla genética. Limitaciones: No aborda completamente sobre sesgos de selección hospitalaria y de información por no cegamiento |
| **7** | **SAMPLE SIZE AND POWER** | | Was sample size appropriate? Was an a priori power analysis conducted? | **5** | Justification: Robust, paired sample (total n = 458). Convenience sample. Includes O. Limitations: No a priori calculations. |
| **8** | **A PRIORI PLANNING OF ANALYSES** | | Was analysis plan appropriate and sufficiently described? Was selective/inappropriate reporting avoided (all test results reported)? Were tested subgroups, interactions, and sensitivity analyses described? Was statistical software identified? | **6** | Justification: Analysis plan (Logistic regression, T-test, Kaplan-Meier). Subgroup analysis, adjustment for gender. Multiple comparisons are considered. Limitations: Software not specified, Bonferroni correction not mentioned |
| **9** | **STATISTICAL METHODS AND CONTROL FOR CONFOUNDING** | | Were important confounders appropriately controlled? Was missing data handled appropriately (<10% missing acceptable)? Were results adjusted for multiple testing? For multi-ethnic studies, did statistical methods (e.g. PCA) control for confounding? | **7** | Justification: Complete and appropriate analysis; confounders are controlled; assumptions are verified. |
| **10** | **TESTING OF ASSUMPTIONS AND INFERENCES** | | Were all assumptions tested? Specifically: i) If haplotypes inferred, was method reported? ii) Were distant relatives/consanguinity tested? iii) Were reported sex and ethnicity checked? | **6** | Justification: They consider ethnic homogeneity, discuss genetic admixture, and distinguish between familial and sporadic cases. Limitations: They do not verify consanguinity or sex, nor do they evaluate ancestry markers. |
| **11** | **APPROPRIATENESS OF INFERENCES DRAWN** | | Were conclusions supported by results and appropriate methods? | **7** | Justification: Conclusions aligned with data, acknowledge limitations, and provide appropriate context. |
|  |  | | **TOTAL SCORE** | **63** | of 77 |
| **FINAL QUALITY RATING:** | | | | **□ Good Quality** | |

| **Article title:** | | Frequency of the IVS4+ 66A-G polymorphism in the alpha-synuclein gene in patients with Parkinson's disease in northwestern Mexico | | | |
| --- | --- | --- | --- | --- | --- |
| **First Author:** | | Ramírez-Jirano, LJ | | | |
| **Year:** | | 2007 | | | |
| **Journal:** | | Revista de Neurología | | | |
| **Study Design:** | | Case-Control Studies | | □ With control groups | |
|  | | | | | |
| **Item** | **Question** | | **Considerations** | **Score (1-7)** | **Notes/Comments** |
| **1** | **RATIONALE FOR STUDY** | | Was a scientific rationale for chosen genes presented to avoid selective reporting of positive results? If GWAS design (hypothesis-free approach), was rationale for selecting this design presented? | **4** | Justification: Provide contextual information that partially justifies the analysis of the variant within the framework of the investigated disorder (Parkinson's disease). Justify conducting the study in a Mexican population. State the objective of the study. Limitations: Limited justification for selecting the analyzed variant. Does not discuss previous inconsistencies in depth. |
| **2** | **SELECTION AND DEFINITION OF OUTCOME** | | Were cases appropriately defined? Were participants appropriately sampled to avoid selection bias? Were case/outcome assessors blinded to genotype status? If applicable, was follow-up length appropriate and attrition rate acceptable? Outcome definitions: independent adjudication/lab measures (strong) vs self-report (moderate) vs no description (poor) | **5** | Justification: Cases (n=51) were evaluated by specialists in specialised clinics using standardised diagnostic criteria. Participants from northwestern Mexico. Limitations: Detailed selection criteria (inclusion/exclusion) are not provided, and blinding is not mentioned. Convenience sample-specific information is provided about ancestry. |
| **3** | **SELECTION AND COMPARABILITY OF COMPARISON GROUPS** | | Were controls appropriately defined? Were controls sampled to minimize selection bias? Was detailed description of selection procedure outlined? Were assessors of control status blinded to genotype? In multi-ethnic studies, were sub-populations (ethnicity) reported? | **3** | Justification: Defined controls (121 individuals from the general population, blood bank donors from a hospital centre) with no history of neurological diseases or dementia. From northwestern Mexico. Limitations: Lacks detailed selection criteria (inclusion/exclusion) and blinding. Convenience sample, unmatched. No mention of age or sex matching. No specific information is given about ancestry. |
| **4** | **TECHNICAL CLASSIFICATION OF EXPOSURE** | | Was DNA source and storage method appropriate? Were DNA ascertainment methods similar for comparison groups? Was genotyping platform and allele-calling algorithm appropriate? Were genotyping error and call rates appropriate (≥95%)? Were genotype call rates and SNP missingness similar between groups? Was Hardy-Weinberg equilibrium tested in controls? Did authors check for outlying heterozygosity? If genotypes imputed, were methods described? | **5** | Justification: The molecular techniques for DNA extraction and genotyping of the referenced polymorphisms are clearly described. It states that genotyping is confirmed by analysing duplicate samples. Hardy-Weinberg equilibrium is verified. Limitations: Specific quality-control metrics (error rates, genotyping recall, SNP loss) are not mentioned. |
| **5** | **NON-TECHNICAL CLASSIFICATION OF EXPOSURE** | | Did blinded assessor conduct genotyping? Was genotyping conducted simultaneously or in batches (same methods)? If applicable, were samples randomized prior to genotyping (not all controls/cases on same plate)? | **4** | Justification: The standardized method used is reproducible. Limitations: Blinding is not mentioned, nor is it specified whether genotyping was simultaneous or in batches. Sample randomization before genotyping is not mentioned. |
| **6** | **OTHER SOURCES OF BIAS** | | Were all sources of bias disclosed and their effect on results discussed? (selection bias, classification bias, time-lag bias, attrition bias, etc.) | **1** | Justification: Justification: Few limitations are acknowledged. Limitations: Selection bias (unmatched volunteer controls), information bias (lack of blinding), age bias (younger controls could develop Parkinson's disease later), and uncontrolled sex bias are not discussed. |
| **7** | **SAMPLE SIZE AND POWER** | | Was sample size appropriate? Was an a priori power analysis conducted? | **2** | Justification: The sample size is not explicitly justified. Matching with more controls is a valid strategy to maximize power; however, age and sex matching are not mentioned. (Mean age of cases: 61.3 years; mean age of controls: 49.2 years). Limitations: No a priori calculations. |
| **8** | **A PRIORI PLANNING OF ANALYSES** | | Was analysis plan appropriate and sufficiently described? Was selective/inappropriate reporting avoided (all test results reported)? Were tested subgroups, interactions, and sensitivity analyses described? Was statistical software identified? | **3** | Statistical analysis plan: χ² and odds ratio with Mantel-Haenszel correction. Software specified. Limitations: Does not analyze subgroups, adjust for gender or age, or perform multiple comparisons. |
| **9** | **STATISTICAL METHODS AND CONTROL FOR CONFOUNDING** | | Were important confounders appropriately controlled? Was missing data handled appropriately (<10% missing acceptable)? Were results adjusted for multiple testing? For multi-ethnic studies, did statistical methods (e.g. PCA) control for confounding? | **3** | Justificación: Análisis básico, calcula frecuencias alélicas y genotípicas, corrección de Mantel-Haenszel y Odds ratios con IC95%, reporta HW equilibrium en controles. Limitaciones. No analiza confesores importantes como edad, sexo ni factores ambientales. |
| **10** | **TESTING OF ASSUMPTIONS AND INFERENCES** | | Were all assumptions tested? Specifically: i) If haplotypes inferred, was method reported? ii) Were distant relatives/consanguinity tested? iii) Were reported sex and ethnicity checked? | **1** | Justification: It does not verify family relationships between participants. It does not verify sex or ethnicity. |
| **11** | **APPROPRIATENESS OF INFERENCES DRAWN** | | Were conclusions supported by results and appropriate methods? | **3** | Justification: Moderately appropriate conclusions: They acknowledge negative results and are cautious in indicating that these data will be useful in describing these frequencies. They do not make inappropriate causal claims. Limitations: They acknowledge few limitations, but there is significant age bias (younger controls could develop PD later). They lack genetic ancestry data, so they cannot verify ethnic variability. |
|  |  | | **TOTAL SCORE** | **34** | of 77 |
| **FINAL QUALITY RATING:** | | | | **□ Poor Quality** | |

| **Article title:** | | Apolipoprotein E Genotypes in Mexican Patients with Parkinson′ s Disease | | | |
| --- | --- | --- | --- | --- | --- |
| **First Author:** | | Gallegos-Arreola, MP | | | |
| **Year:** | | 2009 | | | |
| **Journal:** | | Disease Markers | | | |
| **Study Design:** | | Case-Control Studies | | □ With control groups | |
|  | | | | | |
| **Item** | **Question** | | **Considerations** | **Score (1-7)** | **Notes/Comments** |
| **1** | **RATIONALE FOR STUDY** | | Was a scientific rationale for chosen genes presented to avoid selective reporting of positive results? If GWAS design (hypothesis-free approach), was rationale for selecting this design presented? | **6** | Justification: Provide information justifying the selection of the analyzed variants within the framework of the investigated disorder (Parkinson's disease). To justify conducting the study in a population from the state of Jalisco, Mexico. To state the research objective. |
| **2** | **SELECTION AND DEFINITION OF OUTCOME** | | Were cases appropriately defined? Were participants appropriately sampled to avoid selection bias? Were case/outcome assessors blinded to genotype status? If applicable, was follow-up length appropriate and attrition rate acceptable? Outcome definitions: independent adjudication/lab measures (strong) vs self-report (moderate) vs no description (poor) | **5** | Justification: Cases (n=105) evaluated by specialists using diagnostic criteria. Systematic recruitment and selection at specialized clinics. Participants were from Mexico City and surrounding areas with similar socioeconomic conditions. Detailed family analysis with pedigrees was conducted. Limitation: Blinding is not mentioned—convenience sample. No specific information is given about ancestry. |
| **3** | **SELECTION AND COMPARABILITY OF COMPARISON GROUPS** | | Were controls appropriately defined? Were controls sampled to minimize selection bias? Was detailed description of selection procedure outlined? Were assessors of control status blinded to genotype? In multi-ethnic studies, were sub-populations (ethnicity) reported? | **3** | Justification: They included healthy controls (n=107) without Parkinson's disease or neurodegenerative disorders, with information on habits and family history. Limitations: Blinding was not described. The participants' origins were not described (they were volunteers). They were not matched by age or sex, and significant differences were reported. No specific information is given about ancestry. |
| **4** | **TECHNICAL CLASSIFICATION OF EXPOSURE** | | Was DNA source and storage method appropriate? Were DNA ascertainment methods similar for comparison groups? Was genotyping platform and allele-calling algorithm appropriate? Were genotyping error and call rates appropriate (≥95%)? Were genotype call rates and SNP missingness similar between groups? Was Hardy-Weinberg equilibrium tested in controls? Did authors check for outlying heterozygosity? If genotypes imputed, were methods described? | **4** | Justification: Molecular analysis techniques and standardized protocols are described with references. Hardy-Weinberg equilibrium verification is performed. Frequencies (allelic and genotypic) are reported. Limitations: Genotyping verifications, error rates, genotyping callouts, and SNP loss are not performed. |
| **5** | **NON-TECHNICAL CLASSIFICATION OF EXPOSURE** | | Did blinded assessor conduct genotyping? Was genotyping conducted simultaneously or in batches (same methods)? If applicable, were samples randomized prior to genotyping (not all controls/cases on same plate)? | **3** | Justification: Standardized technical description. Limitations: Does not mention blinding, does not specify whether genotyping was simultaneous or in batches. Does not mention randomisation of the sample before genotyping, nor verification. |
| **6** | **OTHER SOURCES OF BIAS** | | Were all sources of bias disclosed and their effect on results discussed? (selection bias, classification bias, time-lag bias, attrition bias, etc.) | **6** | Justification: Includes an analysis of environmental factors, acknowledges limitations, and indicates that confirmation is required. Limitations: Could be more explicit about selection bias and information bias due to the lack of blinding. |
| **7** | **SAMPLE SIZE AND POWER** | | Was sample size appropriate? Was an a priori power analysis conducted? | **5** | Justification: Includes discussion of environmental factors, acknowledges limitations, and indicates that confirmation is required. Limitations: Does not extensively address selection sessions. Does not mention blinding. |
| **8** | **A PRIORI PLANNING OF ANALYSES** | | Was analysis plan appropriate and sufficiently described? Was selective/inappropriate reporting avoided (all test results reported)? Were tested subgroups, interactions, and sensitivity analyses described? Was statistical software identified? | **6** | Justification: Appropriate analysis. Logistic regression, HW equilibrium verified, control for multiple confounders: age, sex, tobacco, caffeine, pesticides, Odds ratios with reported 95% CI, and analysis of environmental factors. Limitations: The Software used is not declared |
| **9** | **STATISTICAL METHODS AND CONTROL FOR CONFOUNDING** | | Were important confounders appropriately controlled? Was missing data handled appropriately (<10% missing acceptable)? Were results adjusted for multiple testing? For multi-ethnic studies, did statistical methods (e.g. PCA) control for confounding? | **7** | Justification: Appropriate multivariate analysis. |
| **10** | **TESTING OF ASSUMPTIONS AND INFERENCES** | | Were all assumptions tested? Specifically: i) If haplotypes inferred, was method reported? ii) Were distant relatives/consanguinity tested? iii) Were reported sex and ethnicity checked? | **6** | Justification: HWE verified. Limitations: Does not verify sex or consanguinity. |
| **11** | **APPROPRIATENESS OF INFERENCES DRAWN** | | Were conclusions supported by results and appropriate methods? | **5** | Justification: Appropriate conclusions, acknowledge limitations, and are cautious in interpretations—limitations: Significant age bias (younger controls could develop PD later). |
|  |  | | **TOTAL SCORE** | **56** | of 77 |
| **FINAL QUALITY RATING:** | | | | **□ Good Quality** | |

| **Article title:** | | Low frequency of common LRRK2 mutations in Mexican patients with Parkinson's disease | | | |
| --- | --- | --- | --- | --- | --- |
| **First Author:** | | Yescas, P | | | |
| **Year:** | | 2010 | | | |
| **Journal:** | | Neuroscience Letters | | | |
| **Study Design:** | | Case-Control Studies | | □ With control groups | |
|  | | | | | |
| **Item** | **Question** | | **Considerations** | **Score (1-7)** | **Notes/Comments** |
| **1** | **RATIONALE FOR STUDY** | | Was a scientific rationale for chosen genes presented to avoid selective reporting of positive results? If GWAS design (hypothesis-free approach), was rationale for selecting this design presented? | **6** | Justification: Provides information justifying the selection of the analyzed variants within the framework of the investigated disorder (Parkinson's disease). Justifies conducting the study in the Mexican population. States the research objective. |
| **2** | **SELECTION AND DEFINITION OF OUTCOME** | | Were cases appropriately defined? Were participants appropriately sampled to avoid selection bias? Were case/outcome assessors blinded to genotype status? If applicable, was follow-up length appropriate and attrition rate acceptable? Outcome definitions: independent adjudication/lab measures (strong) vs self-report (moderate) vs no description (poor) | **6** | Justification: Cases (n=309) were evaluated using standardized diagnostic criteria. Systematic recruitment and selection were conducted at specialized medical units. Participants were from Mexico City and surrounding metropolitan areas, of Mexican descent across three generations, and from similar socioeconomic backgrounds. Detailed family analysis was performed using pedigrees. Limitation: The study does not specify whether the clinical evaluators were blinded to the genotype—convenience sample. Specific information is given about ancestry. |
| **3** | **SELECTION AND COMPARABILITY OF COMPARISON GROUPS** | | Were controls appropriately defined? Were controls sampled to minimize selection bias? Was detailed description of selection procedure outlined? Were assessors of control status blinded to genotype? In multi-ethnic studies, were sub-populations (ethnicity) reported? | **6** | Justification: The study included healthy controls (n=200) aged 55 and over, with family history information. Participants were from Mexico City and the surrounding areas. They were Mexican mestizos with at least three generations of similar socioeconomic status. A detailed family analysis, including pedigrees, was performed. Limitations: Blinding was not described. The participants' (volunteers') origins were not described. The studies were not matched. Convenience sample. No specific information is given about ancestry. |
| **4** | **TECHNICAL CLASSIFICATION OF EXPOSURE** | | Was DNA source and storage method appropriate? Were DNA ascertainment methods similar for comparison groups? Was genotyping platform and allele-calling algorithm appropriate? Were genotyping error and call rates appropriate (≥95%)? Were genotype call rates and SNP missingness similar between groups? Was Hardy-Weinberg equilibrium tested in controls? Did authors check for outlying heterozygosity? If genotypes imputed, were methods described? | **5** | Justification: Molecular analysis techniques and standardized protocols are described. Confirmation by sequencing of both strands. Limitation: Descriptive frequencies are reported—limitation of analysis of 3 exons (31, 35, 41). |
| **5** | **NON-TECHNICAL CLASSIFICATION OF EXPOSURE** | | Did blinded assessor conduct genotyping? Was genotyping conducted simultaneously or in batches (same methods)? If applicable, were samples randomized prior to genotyping (not all controls/cases on same plate)? | **5** | Justification: They describe a standardized technique and confirmation. Limitations: They do not mention blinding, nor do they specify whether genotyping was simultaneous or in batches. They do not mention randomisation of the sample before genotyping. |
| **6** | **OTHER SOURCES OF BIAS** | | Were all sources of bias disclosed and their effect on results discussed? (selection bias, classification bias, time-lag bias, attrition bias, etc.) | **4** | Justification: They acknowledge the study's limitations. Limitations: They do not discuss: selection bias (convenience sample), information bias (lack of blinding), selective publication bias |
| **7** | **SAMPLE SIZE AND POWER** | | Was sample size appropriate? Was an a priori power analysis conducted? | **5** | Justification: Large sample size (cases n=319, controls n=200). Limitations: No a priori calculations are presented. |
| **8** | **A PRIORI PLANNING OF ANALYSES** | | Was analysis plan appropriate and sufficiently described? Was selective/inappropriate reporting avoided (all test results reported)? Were tested subgroups, interactions, and sensitivity analyses described? Was statistical software identified? | **5** | Justification: Appropriate analysis. Limitations: No inferential statistical analysis (only descriptive frequencies). |
| **9** | **STATISTICAL METHODS AND CONTROL FOR CONFOUNDING** | | Were important confounders appropriately controlled? Was missing data handled appropriately (<10% missing acceptable)? Were results adjusted for multiple testing? For multi-ethnic studies, did statistical methods (e.g. PCA) control for confounding? | **4** | Justification: HW implicitly verified (no mutations in controls), detailed family analysis with pedigrees, and penetrance analysis (progeny tested). Ethnic control: Mexican mestizos >3 generations. Limitations: No inferential statistical analysis (only descriptive frequencies), sex and ethnicity were not verified. |
| **10** | **TESTING OF ASSUMPTIONS AND INFERENCES** | | Were all assumptions tested? Specifically: i) If haplotypes inferred, was method reported? ii) Were distant relatives/consanguinity tested? iii) Were reported sex and ethnicity checked? | **6** | Justification: Detailed family analysis with pedigrees, penetrance analysis (tested progeny) |
| **11** | **APPROPRIATENESS OF INFERENCES DRAWN** | | Were conclusions supported by results and appropriate methods? | **7** | Justification: Appropriate conclusions, they recognize limitations and are cautious in their interpretations. |
|  |  | | **TOTAL SCORE** | **59** | of 77 |
| **FINAL QUALITY RATING:** | | | | **□ Good Quality** | |

| **Article title:** | | PARKIN-coding polymorphisms are not associated with Parkinson's disease in a population from northeastern Mexico | | | |
| --- | --- | --- | --- | --- | --- |
| **First Author:** | | Martinez, HR | | | |
| **Year:** | | 2010 | | | |
| **Journal:** | | Neuroscience Letters | | | |
| **Study Design:** | | Case-Control Studies | | □ With control groups | |
|  | | | | | |
| **Item** | **Question** | | **Considerations** | **Score (1-7)** | **Notes/Comments** |
| **1** | **RATIONALE FOR STUDY** | | Was a scientific rationale for chosen genes presented to avoid selective reporting of positive results? If GWAS design (hypothesis-free approach), was rationale for selecting this design presented? | **6** | Justification: Provide information justifying the selection of the analyzed variants within the framework of the investigated disorder (Parkinson's disease and early-onset and late-onset subgroups). Justifies conducting the study in the Mexican population. States the research objective. |
| **2** | **SELECTION AND DEFINITION OF OUTCOME** | | Were cases appropriately defined? Were participants appropriately sampled to avoid selection bias? Were case/outcome assessors blinded to genotype status? If applicable, was follow-up length appropriate and attrition rate acceptable? Outcome definitions: independent adjudication/lab measures (strong) vs self-report (moderate) vs no description (poor) | **6** | Justification: Cases (n=117) were evaluated using standardized diagnostic criteria. Selection took place in medical units. Participants were born and resided in northeastern Mexico. Subgroups: EOPD (onset at age 50 or younger, n=23) and LOPD (onset after age 50, n=94). Limitations: It is not specified whether the clinical evaluators were unaware of the genotype—convenience sample. No specific information on ancestry was provided. |
| **3** | **SELECTION AND COMPARABILITY OF COMPARISON GROUPS** | | Were controls appropriately defined? Were controls sampled to minimize selection bias? Was detailed description of selection procedure outlined? Were assessors of control status blinded to genotype? In multi-ethnic studies, were sub-populations (ethnicity) reported? | **5** | Justification: Controls without neurodegenerative disorders and a partially described source (spouses or individuals without neurological or psychiatric illnesses). Participants were born and reside in northeastern Mexico—limitations: Non-matched convenience sample (117 PD: 122 controls). Controls are spouses/companions (not the independent general population), and blinding is not described. The selection procedure is not described in detail. Convenience sample.. No specific information is given about ancestry. |
| **4** | **TECHNICAL CLASSIFICATION OF EXPOSURE** | | Was DNA source and storage method appropriate? Were DNA ascertainment methods similar for comparison groups? Was genotyping platform and allele-calling algorithm appropriate? Were genotyping error and call rates appropriate (≥95%)? Were genotype call rates and SNP missingness similar between groups? Was Hardy-Weinberg equilibrium tested in controls? Did authors check for outlying heterozygosity? If genotypes imputed, were methods described? | **5** | Justification: The referenced molecular techniques for DNA extraction and polymorphism genotyping are clearly described. It mentions that genotyping was confirmed by analysing the samples in duplicate. HW equilibrium verification is included. Limitations: No specific quality control is mentioned, nor are error rates, genotyping callouts, or SNP loss. |
| **5** | **NON-TECHNICAL CLASSIFICATION OF EXPOSURE** | | Did blinded assessor conduct genotyping? Was genotyping conducted simultaneously or in batches (same methods)? If applicable, were samples randomized prior to genotyping (not all controls/cases on same plate)? | **5** | Justification: They describe a standardized technique with corroboration. Limitations: They do not mention blinding, nor do they specify whether genotyping was simultaneous or in batches. They do not mention randomisation of the sample before genotyping. |
| **6** | **OTHER SOURCES OF BIAS** | | Were all sources of bias disclosed and their effect on results discussed? (selection bias, classification bias, time-lag bias, attrition bias, etc.) | **4** | Justification: They acknowledge the study's limitations. Limitations: They do not discuss: Selection bias (convenience sample), Information bias (lack of blinding), and Selective publication bias. Age bias (Cases 59±12/ Controls 50±15 years) |
| **7** | **SAMPLE SIZE AND POWER** | | Was sample size appropriate? Was an a priori power analysis conducted? | **4** | Justification: Moderate convenience sample (117/122). No a priori calculation |
| **8** | **A PRIORI PLANNING OF ANALYSES** | | Was analysis plan appropriate and sufficiently described? Was selective/inappropriate reporting avoided (all test results reported)? Were tested subgroups, interactions, and sensitivity analyses described? Was statistical software identified? | **3** | Justification: Basic analysis. Results reported: Case and control frequencies and statistical test: χ². Subgroups. Statistical software is not mentioned. |
| **9** | **STATISTICAL METHODS AND CONTROL FOR CONFOUNDING** | | Were important confounders appropriately controlled? Was missing data handled appropriately (<10% missing acceptable)? Were results adjusted for multiple testing? For multi-ethnic studies, did statistical methods (e.g. PCA) control for confounding? | **2** | Justification: Limited statistical analysis |
| **10** | **TESTING OF ASSUMPTIONS AND INFERENCES** | | Were all assumptions tested? Specifically: i) If haplotypes inferred, was method reported? ii) Were distant relatives/consanguinity tested? iii) Were reported sex and ethnicity checked? | **5** | Justification: The referenced molecular techniques for DNA extraction and polymorphism genotyping are clearly described. It mentions that genotyping was confirmed by analyzing the samples in duplicate. HW equilibrium verification is included. Limitations: No specific quality control is mentioned, nor are error rates, genotyping callouts, or SNP loss. |
| **11** | **APPROPRIATENESS OF INFERENCES DRAWN** | | Were conclusions supported by results and appropriate methods? | **5** | Justification: Appropriate conclusions. No association found. They discuss the implications of the HWE imbalance. |
|  |  | | **TOTAL SCORE** | **50** | of 77 |
| **FINAL QUALITY RATING:** | | | | **□ Good Quality** | |

| **Article title:** | | Detección de mutaciones puntuales en el gen de alfa-sinucleína en pacientes mexicanos con enfermedad de Parkinson y herencia autosómica dominante | | | |
| --- | --- | --- | --- | --- | --- |
| **First Author:** | | Dávila-Ortiz de Motellano, DJ | | | |
| **Year:** | | 2012 | | | |
| **Journal:** | | Archivos de Neurociencias | | | |
| **Study Design:** | | Case Studies | | □ Without control groups | |
|  | | | | | |
| **Item** | **Question** | | **Considerations** | **Score (1-7)** | **Notes/Comments** |
| **1** | **RATIONALE FOR STUDY** | | Was a scientific rationale for chosen genes presented to avoid selective reporting of positive results? If GWAS design (hypothesis-free approach), was rationale for selecting this design presented? | **4** | Justification: Provide information justifying the investigation of three-point mutations in the context of Dominant Autosomic Parkinson disease (DAPD). State the research objective. Limitations: Does not justify conducting the study in the investigated population. Does not justify the design (case study). Evaluates DE, which is not representative of all PD cases. |
| **2** | **SELECTION AND DEFINITION OF OUTCOME** | | Were cases appropriately defined? Were participants appropriately sampled to avoid selection bias? Were case/outcome assessors blinded to genotype status? If applicable, was follow-up length appropriate and attrition rate acceptable? Outcome definitions: independent adjudication/lab measures (strong) vs self-report (moderate) vs no description (poor) | **5** | Justification: Eighty-six cases of autosomal dominant Parkinson's disease (ADPD) were evaluated using standardized diagnostic criteria for Parkinson's disease, applied by specialists. Sequential, non-randomized selection was conducted in specialized clinics by neurologists. Participant interviews, access to medical records, and access to demographic variables were obtained. Limitations: It is not specified whether the clinical evaluators were blinded to the genotype—convenience sample. No specific information is given about ancestry. |
| **3** | **SELECTION AND COMPARABILITY OF COMPARISON GROUPS** | | Were controls appropriately defined? Were controls sampled to minimize selection bias? Was detailed description of selection procedure outlined? Were assessors of control status blinded to genotype? In multi-ethnic studies, were sub-populations (ethnicity) reported? | **0** | Justification: Non-control group |
| **4** | **TECHNICAL CLASSIFICATION OF EXPOSURE** | | Was DNA source and storage method appropriate? Were DNA ascertainment methods similar for comparison groups? Was genotyping platform and allele-calling algorithm appropriate? Were genotyping error and call rates appropriate (≥95%)? Were genotype call rates and SNP missingness similar between groups? Was Hardy-Weinberg equilibrium tested in controls? Did authors check for outlying heterozygosity? If genotypes imputed, were methods described? | **3** | Justification: Describes the molecular techniques used for polymorphism detection. Indicates that the DNA was obtained from a DNA bank. Limitations: Does not mention quality controls. Case study, therefore, the HW equilibrium verification of controls does not apply. Limitations: Does not mention specific quality control measures or error rates |
| **5** | **NON-TECHNICAL CLASSIFICATION OF EXPOSURE** | | Did blinded assessor conduct genotyping? Was genotyping conducted simultaneously or in batches (same methods)? If applicable, were samples randomized prior to genotyping (not all controls/cases on same plate)? | **4** | Justification: They describe a standardized technique. The case study does not mention blinding or randomization. |
| **6** | **OTHER SOURCES OF BIAS** | | Were all sources of bias disclosed and their effect on results discussed? (selection bias, classification bias, time-lag bias, attrition bias, etc.) | **3** | Justification: They acknowledge the study's limitations. Limitations: They do not discuss: Selection bias (convenience sample). Selective publication bias. |
| **7** | **SAMPLE SIZE AND POWER** | | Was sample size appropriate? Was an a priori power analysis conducted? | **1** | Justification: This is a descriptive study. There is no statistical comparison, no statistical analysis (not applicable, no controls), and no power calculation. It does not justify the sample size. |
| **8** | **A PRIORI PLANNING OF ANALYSES** | | Was analysis plan appropriate and sufficiently described? Was selective/inappropriate reporting avoided (all test results reported)? Were tested subgroups, interactions, and sensitivity analyses described? Was statistical software identified? | **3** | Justification: Describes variables. Descriptive study. No statistical comparison. |
| **9** | **STATISTICAL METHODS AND CONTROL FOR CONFOUNDING** | | Were important confounders appropriately controlled? Was missing data handled appropriately (<10% missing acceptable)? Were results adjusted for multiple testing? For multi-ethnic studies, did statistical methods (e.g. PCA) control for confounding? | **1** | Justification: There is no statistical comparison. It describes sociodemographic variables; however, it is a descriptive study. |
| **10** | **TESTING OF ASSUMPTIONS AND INFERENCES** | | Were all assumptions tested? Specifically: i) If haplotypes inferred, was method reported? ii) Were distant relatives/consanguinity tested? iii) Were reported sex and ethnicity checked? | **3** | Justification: Adequate descriptive conclusion (No mutations found in the sample). |
| **11** | **APPROPRIATENESS OF INFERENCES DRAWN** | | Were conclusions supported by results and appropriate methods? | **5** | Justification: Appropriate conclusions (no mutations found) are cautious, noting that further studies are needed to rule out the direct involvement of the SNCA gene in the pathogenesis of DAPD. |
|  |  | | **TOTAL SCORE** | **32** | of 77 |
| **FINAL QUALITY RATING:** | | | | **□ Moderate Quality** | |

| **Article title:** | | High frequency of Parkin exon rearrangements in Mexican‐mestizo patients with early‐onset Parkinson's disease | | | |
| --- | --- | --- | --- | --- | --- |
| **First Author:** | | Guerrero Camacho, JL | | | |
| **Year:** | | 2012 | | | |
| **Journal:** | | Movement Disorders | | | |
| **Study Design:** | | Case-Control Studies | | □ With control groups | |
|  | | | | | |
| **Item** | **Question** | | **Considerations** | **Score (1-7)** | **Notes/Comments** |
| **1** | **RATIONALE FOR STUDY** | | Was a scientific rationale for chosen genes presented to avoid selective reporting of positive results? If GWAS design (hypothesis-free approach), was rationale for selecting this design presented? | **7** | Justification: Provide information justifying the research within the context of the investigated disorder (early-onset Parkinson's disease). State the research objective. Justify conducting the study in the Mexican population. |
| **2** | **SELECTION AND DEFINITION OF OUTCOME** | | Were cases appropriately defined? Were participants appropriately sampled to avoid selection bias? Were case/outcome assessors blinded to genotype status? If applicable, was follow-up length appropriate and attrition rate acceptable? Outcome definitions: independent adjudication/lab measures (strong) vs self-report (moderate) vs no description (poor) | **7** | Justification: Cases of early-onset Parkinson's disease (EOPD) were included (n=63, with a mean age of onset of 37.3 years). They were evaluated by specialists using standardised diagnostic criteria. Selection was conducted at specialized clinics. Systematic recruitment was used. Participants were of Mexican descent across three generations. Limitations: It is not specified whether the clinical evaluators were unaware of the genotype—convenience sample. |
| **3** | **SELECTION AND COMPARABILITY OF COMPARISON GROUPS** | | Were controls appropriately defined? Were controls sampled to minimize selection bias? Was detailed description of selection procedure outlined? Were assessors of control status blinded to genotype? In multi-ethnic studies, were sub-populations (ethnicity) reported? | **6** | Justification: Controls (n=120) were healthy volunteers >45 years old, with no family history of movement disorders, of Mexican ancestry across three generations. They had socioeconomic conditions similar to those of the cases. Participants were of Mexican ancestry across three generations. Limitations: The selection procedure was not described in detail—convenience sample. Blinding was not described. |
| **4** | **TECHNICAL CLASSIFICATION OF EXPOSURE** | | Was DNA source and storage method appropriate? Were DNA ascertainment methods similar for comparison groups? Was genotyping platform and allele-calling algorithm appropriate? Were genotyping error and call rates appropriate (≥95%)? Were genotype call rates and SNP missingness similar between groups? Was Hardy-Weinberg equilibrium tested in controls? Did authors check for outlying heterozygosity? If genotypes imputed, were methods described? | **7** | Justification: The molecular analysis techniques are clearly described, including triplicate testing of positive results and the use of positive and negative controls. Hardy-Weinberg equilibrium (HWE) was not explicitly verified (although it is difficult to apply to rare mutations). |
| **5** | **NON-TECHNICAL CLASSIFICATION OF EXPOSURE** | | Did blinded assessor conduct genotyping? Was genotyping conducted simultaneously or in batches (same methods)? If applicable, were samples randomized prior to genotyping (not all controls/cases on same plate)? | **5** | Justification: Standardised technique and confirmation are described. Limitations: Blinding is not mentioned, nor is it specified whether genotyping was simultaneous or in batches. Sample randomization prior to genotyping is not mentioned. |
| **6** | **OTHER SOURCES OF BIAS** | | Were all sources of bias disclosed and their effect on results discussed? (selection bias, classification bias, time-lag bias, attrition bias, etc.) | **6** | Justification: The authors acknowledge the study's limitations. |
| **7** | **SAMPLE SIZE AND POWER** | | Was sample size appropriate? Was an a priori power analysis conducted? | **5** | Justification: The unmatched design with 63/120 is methodologically valid and efficient—limitations: No a priori power calculation. |
| **8** | **A PRIORI PLANNING OF ANALYSES** | | Was analysis plan appropriate and sufficiently described? Was selective/inappropriate reporting avoided (all test results reported)? Were tested subgroups, interactions, and sensitivity analyses described? Was statistical software identified? | **6** | Justification: Appropriate statistical analysis |
| **9** | **STATISTICAL METHODS AND CONTROL FOR CONFOUNDING** | | Were important confounders appropriately controlled? Was missing data handled appropriately (<10% missing acceptable)? Were results adjusted for multiple testing? For multi-ethnic studies, did statistical methods (e.g. PCA) control for confounding? | **7** | Justification: Appropriate statistical analysis: t-test, χ², Fisher's test, detailed pedigree analysis with family segregation, Odds ratios with 95% CIs reported, genotype–phenotype correlation analysis (AAO vs. affected region). Statistical software is indicated. |
| **10** | **TESTING OF ASSUMPTIONS AND INFERENCES** | | Were all assumptions tested? Specifically: i) If haplotypes inferred, was method reported? ii) Were distant relatives/consanguinity tested? iii) Were reported sex and ethnicity checked? | **6** | Justification: Appropriate analysis: detailed pedigree analysis with family segregation, Odds ratios with 95% CIs, genotype–phenotype correlation analysis (AAO vs. affected region). |
| **11** | **APPROPRIATENESS OF INFERENCES DRAWN** | | Were conclusions supported by results and appropriate methods? | **7** | Justification: Conclusions are appropriate; new findings are reported. |
|  |  | | **TOTAL SCORE** | **69** | of 77 |
| **FINAL QUALITY RATING:** | | | | **□ Good Quality** | |

| **Article title:** | | The L444P GBA mutation is associated with early‐onset Parkinson's disease in Mexican Mestizos | | | |
| --- | --- | --- | --- | --- | --- |
| **First Author:** | | Gonzalez-del Rincon, M de L | | | |
| **Year:** | | 2013 | | | |
| **Journal:** | | Clinical Genetics | | | |
| **Study Design:** | | Case-Control Studies | | □ With control groups | |
|  | | | | | |
| **Item** | **Question** | | **Considerations** | **Score (1-7)** | **Notes/Comments** |
| **1** | **RATIONALE FOR STUDY** | | Was a scientific rationale for chosen genes presented to avoid selective reporting of positive results? If GWAS design (hypothesis-free approach), was rationale for selecting this design presented? | **7** | Justification: Biological rationale justifying the selection of the analyzed variants within the context of the investigated disorder (Parkinson's disease). State the research objective. Justify conducting the study in the Mexican population. Clinical relevance. Post-hoc power calculation. |
| **2** | **SELECTION AND DEFINITION OF OUTCOME** | | Were cases appropriately defined? Were participants appropriately sampled to avoid selection bias? Were case/outcome assessors blinded to genotype status? If applicable, was follow-up length appropriate and attrition rate acceptable? Outcome definitions: independent adjudication/lab measures (strong) vs self-report (moderate) vs no description (poor) | **7** | Justification: Cases of early-onset Parkinson's disease (EOPD) were included (n=128, onset <45 years). They were evaluated by specialists using standardised diagnostic criteria. They were assessed with psychometric tests (Beck Depression Inventory, Cognistat test and DSM-IV-TR). Participants were of Mexican descent across three generations. Limitations: It is not specified whether the clinical evaluators were unaware of the genotype |
| **3** | **SELECTION AND COMPARABILITY OF COMPARISON GROUPS** | | Were controls appropriately defined? Were controls sampled to minimize selection bias? Was detailed description of selection procedure outlined? Were assessors of control status blinded to genotype? In multi-ethnic studies, were sub-populations (ethnicity) reported? | **7** | Justification: Two control groups. Control 1 (n=128, matched for age and sex, with no family history of neurodegenerative disorders). Control 2 (n=124, age >60 years). This minimizes the risk of including presymptomatic individuals. Participants were of Mexican descent across three generations. Limitations: It is not specified whether the clinical evaluators were unaware of the genotype. |
| **4** | **TECHNICAL CLASSIFICATION OF EXPOSURE** | | Was DNA source and storage method appropriate? Were DNA ascertainment methods similar for comparison groups? Was genotyping platform and allele-calling algorithm appropriate? Were genotyping error and call rates appropriate (≥95%)? Were genotype call rates and SNP missingness similar between groups? Was Hardy-Weinberg equilibrium tested in controls? Did authors check for outlying heterozygosity? If genotypes imputed, were methods described? | **6** | Justification: The referenced molecular techniques are clearly described, including positive controls and verification of positive results by sequencing. The HW equilibrium was not explicitly verified (although it is not appropriate for rare mutations)—limitations: Specific call rate not reported. DNA storage is not described. |
| **5** | **NON-TECHNICAL CLASSIFICATION OF EXPOSURE** | | Did blinded assessor conduct genotyping? Was genotyping conducted simultaneously or in batches (same methods)? If applicable, were samples randomized prior to genotyping (not all controls/cases on same plate)? | **3** | Justification: No blinding, but rigorous data collection. |
| **6** | **OTHER SOURCES OF BIAS** | | Were all sources of bias disclosed and their effect on results discussed? (selection bias, classification bias, time-lag bias, attrition bias, etc.) | **6** | Justification: No blinding, but rigorous data collection. |
| **7** | **SAMPLE SIZE AND POWER** | | Was sample size appropriate? Was an a priori power analysis conducted? | **5** | Justification: Robust sample (128 EOPD + 252 controls) increases power. Post-hoc power calculation; limitation acknowledged: "Sample may still be small." Limitations: No formal power calculation presented; limitation recognized for complete GBA analysis |
| **8** | **A PRIORI PLANNING OF ANALYSES** | | Was analysis plan appropriate and sufficiently described? Was selective/inappropriate reporting avoided (all test results reported)? Were tested subgroups, interactions, and sensitivity analyses described? Was statistical software identified? | **5** | Justification: Appropriate and predefined analysis; examines psychiatric symptoms, cognitive decline, and family history post-hoc. Limitations: Statistical software not specified. Some analyses are exploratory (not a priori). |
| **9** | **STATISTICAL METHODS AND CONTROL FOR CONFOUNDING** | | Were important confounders appropriately controlled? Was missing data handled appropriately (<10% missing acceptable)? Were results adjusted for multiple testing? For multi-ethnic studies, did statistical methods (e.g. PCA) control for confounding? | **6** | Justification: Appropriate tests: Fisher's exact test, ORs with 95% CIs. Control for confounders such as age, sex, and ethnicity—limitations: No adjustment for multiple comparisons. |
| **10** | **TESTING OF ASSUMPTIONS AND INFERENCES** | | Were all assumptions tested? Specifically: i) If haplotypes inferred, was method reported? ii) Were distant relatives/consanguinity tested? iii) Were reported sex and ethnicity checked? | **4** | Justification: Verification of ethnic homogeneity and positive controls. Limitations: Hardy-Weinberg equilibrium not reported (although difficult to apply to rare mutations); hidden familial relationships not verified. |
| **11** | **APPROPRIATENESS OF INFERENCES DRAWN** | | Were conclusions supported by results and appropriate methods? | **7** | Justification: Conclusions are appropriate and well supported. Interpretation is adequate. Discussion of biological implications included. Hypothesis on penetrance proposed; need for further studies acknowledged. |
|  |  | | **TOTAL SCORE** | **63** | of 77 |
| **FINAL QUALITY RATING:** | | | | **□ Good Quality** | |

| **Article title:** | | Genetic mutations in early‐onset Parkinson's disease Mexican patients: molecular testing implications | | | |
| --- | --- | --- | --- | --- | --- |
| **First Author:** | | Monroy-Jaramillo, N | | | |
| **Year:** | | 2014 | | | |
| **Journal:** | | American Journal of Medical Genetics Part B: Neuropsychiatric Genetics | | | |
| **Study Design:** | | Case-Control Studies | | □ With control groups | |
|  | | | | | |
| **Item** | **Question** | | **Considerations** | **Score (1-7)** | **Notes/Comments** |
| **1** | **RATIONALE FOR STUDY** | | Was a scientific rationale for chosen genes presented to avoid selective reporting of positive results? If GWAS design (hypothesis-free approach), was rationale for selecting this design presented? | **7** | Justification: Provide information justifying comprehensive gene screening in the context of the disorder under investigation (early-onset Parkinson's disease). State the research objective. Justify conducting the study in the Mexican population. Clinical relevance. |
| **2** | **SELECTION AND DEFINITION OF OUTCOME** | | Were cases appropriately defined? Were participants appropriately sampled to avoid selection bias? Were case/outcome assessors blinded to genotype status? If applicable, was follow-up length appropriate and attrition rate acceptable? Outcome definitions: independent adjudication/lab measures (strong) vs self-report (moderate) vs no description (poor) | **6** | Justification: Cases of early-onset Parkinson's disease (EOPD) were included (n=127, mean age of onset 34.9 years, sex not specified). They were evaluated by specialists using standardised diagnostic criteria. Selection was conducted in specialised clinics through systematic recruitment. Participants were of Mexican descent across three generations. Limitations: It is not specified whether the clinical evaluators were unaware of the genotype—this is a convenience sample. |
| **3** | **SELECTION AND COMPARABILITY OF COMPARISON GROUPS** | | Were controls appropriately defined? Were controls sampled to minimize selection bias? Was detailed description of selection procedure outlined? Were assessors of control status blinded to genotype? In multi-ethnic studies, were sub-populations (ethnicity) reported? | **6** | Justification: 120 controls aged ≥45 years (60% women, 40% men) with no family history of movement disorders from Mexico City and its surrounding areas were included. They had similar socioeconomic conditions. The participants were of Mexican descent and belonged to three generations. Limitations: It is not specified whether the clinical evaluators were unaware of the genotype—this is a convenience sample. |
| **4** | **TECHNICAL CLASSIFICATION OF EXPOSURE** | | Was DNA source and storage method appropriate? Were DNA ascertainment methods similar for comparison groups? Was genotyping platform and allele-calling algorithm appropriate? Were genotyping error and call rates appropriate (≥95%)? Were genotype call rates and SNP missingness similar between groups? Was Hardy-Weinberg equilibrium tested in controls? Did authors check for outlying heterozygosity? If genotypes imputed, were methods described? | **6** | Justification: The referenced molecular techniques are clearly described and confirmed. Limitations: Does not describe DNA collection or storage techniques. |
| **5** | **NON-TECHNICAL CLASSIFICATION OF EXPOSURE** | | Did blinded assessor conduct genotyping? Was genotyping conducted simultaneously or in batches (same methods)? If applicable, were samples randomized prior to genotyping (not all controls/cases on same plate)? | **3** | Justification: Blinding and randomisation are not mentioned, but confirmation procedures are reported. |
| **6** | **OTHER SOURCES OF BIAS** | | Were all sources of bias disclosed and their effect on results discussed? (selection bias, classification bias, time-lag bias, attrition bias, etc.) | **6** | Justification: Limitations discussed: sample size. Limitations: Family members are not available to determine the CNV phase. |
| **7** | **SAMPLE SIZE AND POWER** | | Was sample size appropriate? Was an a priori power analysis conducted? | **5** | Justification: Small sample size acknowledged for some comparisons. |
| **8** | **A PRIORI PLANNING OF ANALYSES** | | Was analysis plan appropriate and sufficiently described? Was selective/inappropriate reporting avoided (all test results reported)? Were tested subgroups, interactions, and sensitivity analyses described? Was statistical software identified? | **6** | Justification: Analyses are specified. A step-by-step strategy is proposed. Limitation: Statistical software reported. |
| **9** | **STATISTICAL METHODS AND CONTROL FOR CONFOUNDING** | | Were important confounders appropriately controlled? Was missing data handled appropriately (<10% missing acceptable)? Were results adjusted for multiple testing? For multi-ethnic studies, did statistical methods (e.g. PCA) control for confounding? | **6** | Justification: Appropriate analyses (χ², Odds ratios). Statistical software reported—limitation: No adjustment for multiple comparisons. |
| **10** | **TESTING OF ASSUMPTIONS AND INFERENCES** | | Were all assumptions tested? Specifically: i) If haplotypes inferred, was method reported? ii) Were distant relatives/consanguinity tested? iii) Were reported sex and ethnicity checked? | **6** | Justification: In silico analysis performed (ExPASy Translate tool). Prediction of protein effects included. |
| **11** | **APPROPRIATENESS OF INFERENCES DRAWN** | | Were conclusions supported by results and appropriate methods? | **7** | Justification: Well-supported conclusions with clinical implications. A diagnostic algorithm is proposed for Mexican mestizo patients with EOPD. |
|  |  | | **TOTAL SCORE** | **64** | of 77 |
| **FINAL QUALITY RATING:** | | | | **□ Good Quality** | |

| **Article title:** | | Low prevalence of most frequent pathogenic variants of six PARK genes in sporadic Parkinson's disease | | | |
| --- | --- | --- | --- | --- | --- |
| **First Author:** | | Garcia, S. | | | |
| **Year:** | | 2014 | | | |
| **Journal:** | | Folia Neuropathologica | | | |
| **Study Design:** | | Case-Control Studies | | □ With control groups | |
|  | | | | | |
| **Item** | **Question** | | **Considerations** | **Score (1-7)** | **Notes/Comments** |
| **1** | **RATIONALE FOR STUDY** | | Was a scientific rationale for chosen genes presented to avoid selective reporting of positive results? If GWAS design (hypothesis-free approach), was rationale for selecting this design presented? | **7** | Justification: Provide information justifying the analysis of variants in the selected genes within the context of the investigated disorder (sporadic Parkinson's disease). State the objective of the research. Justify conducting the study in the Mexican population—sample size calculation. |
| **2** | **SELECTION AND DEFINITION OF OUTCOME** | | Were cases appropriately defined? Were participants appropriately sampled to avoid selection bias? Were case/outcome assessors blinded to genotype status? If applicable, was follow-up length appropriate and attrition rate acceptable? Outcome definitions: independent adjudication/lab measures (strong) vs self-report (moderate) vs no description (poor) | **6** | Justification:173 cases of PD were included. Subgroup of EOPD (defined as age of onset of PD 20 to 40 years, n=19 years). They were evaluated by specialists using standardised diagnostic criteria. Selection was conducted in specialised clinics through systematic recruitment. Participants were of Mexican descent across three generations. Limitations: It is not specified whether the clinical evaluators were unaware of the genotype—convenience sample. |
| **3** | **SELECTION AND COMPARABILITY OF COMPARISON GROUPS** | | Were controls appropriately defined? Were controls sampled to minimize selection bias? Was detailed description of selection procedure outlined? Were assessors of control status blinded to genotype? In multi-ethnic studies, were sub-populations (ethnicity) reported? | **5** | Justification: 208 controls aged ≥45 years (60% women, 40% men) with no family history of movement disorders from Mexico City and its surrounding areas were included. They had similar socioeconomic conditions. The participants were of Mexican descent and belonged to three generations. Limitations: It is not specified whether the clinical evaluators were unaware of the genotype—unpaired convenience sample. Details of the selection process (inclusion/exclusion) are not described. |
| **4** | **TECHNICAL CLASSIFICATION OF EXPOSURE** | | Was DNA source and storage method appropriate? Were DNA ascertainment methods similar for comparison groups? Was genotyping platform and allele-calling algorithm appropriate? Were genotyping error and call rates appropriate (≥95%)? Were genotype call rates and SNP missingness similar between groups? Was Hardy-Weinberg equilibrium tested in controls? Did authors check for outlying heterozygosity? If genotypes imputed, were methods described? | **6** | Justification: The molecular techniques for DNA extraction and genotyping are clearly described (Real-time PCR, confirmatory sequencing, MLPA for CNVs). HWE verified. |
| **5** | **NON-TECHNICAL CLASSIFICATION OF EXPOSURE** | | Did blinded assessor conduct genotyping? Was genotyping conducted simultaneously or in batches (same methods)? If applicable, were samples randomized prior to genotyping (not all controls/cases on same plate)? | **4** | Justification: Blinding and randomization are not explicitly mentioned. |
| **6** | **OTHER SOURCES OF BIAS** | | Were all sources of bias disclosed and their effect on results discussed? (selection bias, classification bias, time-lag bias, attrition bias, etc.) | **5** | Justification: Selection and ancestry biases are discussed. Limitations: Blinding and randomization are not explicitly mentioned. |
| **7** | **SAMPLE SIZE AND POWER** | | Was sample size appropriate? Was an a priori power analysis conducted? | **7** | N = 381 (173 cases, 208 controls). Sample size calculated using the Armitage trend test. |
| **8** | **A PRIORI PLANNING OF ANALYSES** | | Was analysis plan appropriate and sufficiently described? Was selective/inappropriate reporting avoided (all test results reported)? Were tested subgroups, interactions, and sensitivity analyses described? Was statistical software identified? | **6** | Justification: Appropriate statistical analysis; tests specified (t-test, χ², HWE); statistical software indicated (SPSS v18.0)—limitations: No adjustment for multiple comparisons mentioned. |
| **9** | **STATISTICAL METHODS AND CONTROL FOR CONFOUNDING** | | Were important confounders appropriately controlled? Was missing data handled appropriately (<10% missing acceptable)? Were results adjusted for multiple testing? For multi-ethnic studies, did statistical methods (e.g. PCA) control for confounding? | **5** | Justification: Appropriate statistical analysis; tests specified (t-test, χ², HWE); statistical software indicated (SPSS v18.0). Limitations: No adjustment for multiple comparisons mentioned.Limitation: No adjustment for multiple comparisons mentioned. |
| **10** | **TESTING OF ASSUMPTIONS AND INFERENCES** | | Were all assumptions tested? Specifically: i) If haplotypes inferred, was method reported? ii) Were distant relatives/consanguinity tested? iii) Were reported sex and ethnicity checked? | **5** | Justification: HWE analyzed—limitations: Sex, ethnicity, and consanguinity not verified. |
| **11** | **APPROPRIATENESS OF INFERENCES DRAWN** | | Were conclusions supported by results and appropriate methods? | **6** | Justification: Cautious conclusions; indicates the need to investigate population-specific pathogenic variants in PARK genes and other related genes. |
|  |  | | **TOTAL SCORE** | **62** | of 77 |
| **FINAL QUALITY RATING:** | | | | **□ Good Quality** | |

| **Article title:** | | Relación entre el polimorfismo DRD2/ANKK1 y el desarrollo de complicaciones motoras en enfermedad de Parkinson | | | |
| --- | --- | --- | --- | --- | --- |
| **First Author:** | | Cervantes-Arriaga, A | | | |
| **Year:** | | 2015 | | | |
| **Journal:** | | Neurología Argentina | | | |
| **Study Design:** | | Case Studies | | □ Without control groups | |
|  | | | | | |
| **Item** | **Question** | | **Considerations** | **Score (1-7)** | **Notes/Comments** |
| **1** | **RATIONALE FOR STUDY** | | Was a scientific rationale for chosen genes presented to avoid selective reporting of positive results? If GWAS design (hypothesis-free approach), was rationale for selecting this design presented? | **7** | Justification: Provides information justifying the analysis of variants in the selected genes within the context of the investigated disorder (motor complications arising from dopamine replacement therapy in sporadic Parkinson's disease). States the research objective. Justifies conducting the study in the Mexican population. |
| **2** | **SELECTION AND DEFINITION OF OUTCOME** | | Were cases appropriately defined? Were participants appropriately sampled to avoid selection bias? Were case/outcome assessors blinded to genotype status? If applicable, was follow-up length appropriate and attrition rate acceptable? Outcome definitions: independent adjudication/lab measures (strong) vs self-report (moderate) vs no description (poor) | **6** | Justification: Evaluation of motor complications by a specialist in movement disorders in specialised medical units. Information on age, gender, family history, age of onset of the disorder, severity of the disease, and motor score. Medication dosage, duration of levodopa use. Limitation: Blinding was not mentioned. Convenience sample. No specific information on ancestry was provided. |
| **3** | **SELECTION AND COMPARABILITY OF COMPARISON GROUPS** | | Were controls appropriately defined? Were controls sampled to minimize selection bias? Was detailed description of selection procedure outlined? Were assessors of control status blinded to genotype? In multi-ethnic studies, were sub-populations (ethnicity) reported? | **5** | Justification: Design appropriate for phenotype-genotype association. Study with PD cases (n=236) with subgroups: With complications / Without complications. With defined clinical variables. Limitations: Blinding was not mentioned. Convenience sample. |
| **4** | **TECHNICAL CLASSIFICATION OF EXPOSURE** | | Was DNA source and storage method appropriate? Were DNA ascertainment methods similar for comparison groups? Was genotyping platform and allele-calling algorithm appropriate? Were genotyping error and call rates appropriate (≥95%)? Were genotype call rates and SNP missingness similar between groups? Was Hardy-Weinberg equilibrium tested in controls? Did authors check for outlying heterozygosity? If genotypes imputed, were methods described? | **4** | Justification: The referenced molecular techniques are mentioned. Limitations: DNA extraction and storage techniques are not described. Confirmation of results not mentioned. HWE was not explicitly reported. |
| **5** | **NON-TECHNICAL CLASSIFICATION OF EXPOSURE** | | Did blinded assessor conduct genotyping? Was genotyping conducted simultaneously or in batches (same methods)? If applicable, were samples randomized prior to genotyping (not all controls/cases on same plate)? | **3** | Justification: Blinding was not mentioned; it is not specified whether genotyping was performed simultaneously or in batches. Randomization of samples prior to genotyping not mentioned. |
| **6** | **OTHER SOURCES OF BIAS** | | Were all sources of bias disclosed and their effect on results discussed? (selection bias, classification bias, time-lag bias, attrition bias, etc.) | **5** | Justification: Limitations discussed: sample size, single reference centre (not generalizable), categorical assessment of dyskinesias (lack of specific scale). |
| **7** | **SAMPLE SIZE AND POWER** | | Was sample size appropriate? Was an a priori power analysis conducted? | **4** | Justification: Sample size recognized as relatively small but comparable to similar PD studies and related to levodopa treatment complications. Limitation: No a priori power calculation reported. |
| **8** | **A PRIORI PLANNING OF ANALYSES** | | Was analysis plan appropriate and sufficiently described? Was selective/inappropriate reporting avoided (all test results reported)? Were tested subgroups, interactions, and sensitivity analyses described? Was statistical software identified? | **6** | Justification: Appropriate statistical analyses: χ² with Yates correction, Fisher’s exact test, logistic regression. Multivariate regression analysis performed. Statistical software reported (STATA 12). |
| **9** | **STATISTICAL METHODS AND CONTROL FOR CONFOUNDING** | | Were important confounders appropriately controlled? Was missing data handled appropriately (<10% missing acceptable)? Were results adjusted for multiple testing? For multi-ethnic studies, did statistical methods (e.g. PCA) control for confounding? | **6** | Justification: Appropriate control of treatment-related confounders. Multivariate regression including levodopa use, LEDD, and genotype. |
| **10** | **TESTING OF ASSUMPTIONS AND INFERENCES** | | Were all assumptions tested? Specifically: i) If haplotypes inferred, was method reported? ii) Were distant relatives/consanguinity tested? iii) Were reported sex and ethnicity checked? | **5** | Justification: Bivariate analysis performed prior to multivariate analysis. Limitation: Population structure not discussed. |
| **11** | **APPROPRIATENESS OF INFERENCES DRAWN** | | Were conclusions supported by results and appropriate methods? | **5** | Justification: Cautious and appropriate conclusions. The authors acknowledge the need for a larger sample and further validation. |
|  |  | | **TOTAL SCORE** | **56** | of 77 |
| **FINAL QUALITY RATING:** | | | | **□ Good Quality** | |

| **Article title:** | | Association of the rs1801133 variant in the MTHFR gene and sporadic Parkinson's disease | | | |
| --- | --- | --- | --- | --- | --- |
| **First Author:** | | Garcia, S. | | | |
| **Year:** | | 2015 | | | |
| **Journal:** | | Folia Neuropathologica | | | |
| **Study Design:** | | Case-Control Studies | | □ With control groups | |
|  | | | | | |
| **Item** | **Question** | | **Considerations** | **Score (1-7)** | **Notes/Comments** |
| **1** | **RATIONALE FOR STUDY** | | Was a scientific rationale for chosen genes presented to avoid selective reporting of positive results? If GWAS design (hypothesis-free approach), was rationale for selecting this design presented? | **6** | Justification: Provides information justifying the analysis of the variant within the context of the investigated disorder (sporadic Parkinson's disease). States the research objective. Justifies conducting the study in the Mexican population. Power calculated a posteriori. |
| **2** | **SELECTION AND DEFINITION OF OUTCOME** | | Were cases appropriately defined? Were participants appropriately sampled to avoid selection bias? Were case/outcome assessors blinded to genotype status? If applicable, was follow-up length appropriate and attrition rate acceptable? Outcome definitions: independent adjudication/lab measures (strong) vs self-report (moderate) vs no description (poor) | **6** | Justification: Cases of sporadic Parkinson's disease (n=140, 68% male, 32% female, mean age 65.46), evaluated using standardized diagnostic criteria. Selection at specialized clinics. Systematic recruitment. Cognitive assessment (Folstein Mini-Mental State Examination). Participants of Mexican descent across three generations. Limitations: It is not specified whether the clinical evaluators were unaware of the genotype—unpaired convenience sample. |
| **3** | **SELECTION AND COMPARABILITY OF COMPARISON GROUPS** | | Were controls appropriately defined? Were controls sampled to minimize selection bias? Was detailed description of selection procedure outlined? Were assessors of control status blinded to genotype? In multi-ethnic studies, were sub-populations (ethnicity) reported? | **6** | Justification: Healthy controls (n=216, 65% male, 35% female, mean age 63.6) were blood bank donors or spouses of patients. They had no family history of neurodegenerative diseases and were of Mexican descent for three generations. Limitations: It is not specified whether the clinical evaluators were unaware of the genotype. This was an unpaired convenience sample. |
| **4** | **TECHNICAL CLASSIFICATION OF EXPOSURE** | | Was DNA source and storage method appropriate? Were DNA ascertainment methods similar for comparison groups? Was genotyping platform and allele-calling algorithm appropriate? Were genotyping error and call rates appropriate (≥95%)? Were genotype call rates and SNP missingness similar between groups? Was Hardy-Weinberg equilibrium tested in controls? Did authors check for outlying heterozygosity? If genotypes imputed, were methods described? | **6** | Justification: The referenced molecular techniques for DNA extraction (DTAB-CTAB method) and genotyping (PCR using TaqMan probes) are described, with confirmation by melting curve analysis. HW equilibrium verified. |
| **5** | **NON-TECHNICAL CLASSIFICATION OF EXPOSURE** | | Did blinded assessor conduct genotyping? Was genotyping conducted simultaneously or in batches (same methods)? If applicable, were samples randomized prior to genotyping (not all controls/cases on same plate)? | **3** | Justification: Blinding and randomization are not mentioned. |
| **6** | **OTHER SOURCES OF BIAS** | | Were all sources of bias disclosed and their effect on results discussed? (selection bias, classification bias, time-lag bias, attrition bias, etc.) | **6** | Justification: Limitations acknowledged: homocysteine not measured, global methylation not assessed, hypertension not evaluated. Post-hoc power calculated (72%). |
| **7** | **SAMPLE SIZE AND POWER** | | Was sample size appropriate? Was an a priori power analysis conducted? | **5** | Justification: N = 356 (140 cases, 216 controls). Post-hoc power calculated (72%). |
| **8** | **A PRIORI PLANNING OF ANALYSES** | | Was analysis plan appropriate and sufficiently described? Was selective/inappropriate reporting avoided (all test results reported)? Were tested subgroups, interactions, and sensitivity analyses described? Was statistical software identified? | **6** | Justification: Appropriate analysis: χ², logistic regression adjusted for smoking and sex, ANOVA. Statistical software indicated (SPSS v18.0). |
| **9** | **STATISTICAL METHODS AND CONTROL FOR CONFOUNDING** | | Were important confounders appropriately controlled? Was missing data handled appropriately (<10% missing acceptable)? Were results adjusted for multiple testing? For multi-ethnic studies, did statistical methods (e.g. PCA) control for confounding? | **6** | Justification: Appropriate: logistic regression adjusted for smoking and sex. |
| **10** | **TESTING OF ASSUMPTIONS AND INFERENCES** | | Were all assumptions tested? Specifically: i) If haplotypes inferred, was method reported? ii) Were distant relatives/consanguinity tested? iii) Were reported sex and ethnicity checked? | **5** | Justification: HW equilibrium tested. Verification of sex, ethnicity, and consanguinity is not mentioned. |
| **11** | **APPROPRIATENESS OF INFERENCES DRAWN** | | Were conclusions supported by results and appropriate methods? | **6** | Justification: Conclusions are cautious and appropriate |
|  |  | | **TOTAL SCORE** | **61** | of 77 |
| **FINAL QUALITY RATING:** | | | | **□ Good Quality** | |

| **Article title:** | | A variante rs3857059 do gene SNCA é associada à doença de Parkinson em mestiços mexicanos | | | |
| --- | --- | --- | --- | --- | --- |
| **First Author:** | | Garcia, S. | | | |
| **Year:** | | 2016 | | | |
| **Journal:** | | Arquivos de Neuro-Psiquiatria | | | |
| **Study Design:** | | Case-Control Studies | | □ With control groups | |
|  | | | | | |
| **Item** | **Question** | | **Considerations** | **Score (1-7)** | **Notes/Comments** |
| **1** | **RATIONALE FOR STUDY** | | Was a scientific rationale for chosen genes presented to avoid selective reporting of positive results? If GWAS design (hypothesis-free approach), was rationale for selecting this design presented? | **6** | Justification: Provides information justifying the analysis of the variant within the context of the investigated disorder (Parkinson's disease). States the research objective. Justifies conducting the study in the Mexican population. Limitations: Does not mention the calculation of statistical power. |
| **2** | **SELECTION AND DEFINITION OF OUTCOME** | | Were cases appropriately defined? Were participants appropriately sampled to avoid selection bias? Were case/outcome assessors blinded to genotype status? If applicable, was follow-up length appropriate and attrition rate acceptable? Outcome definitions: independent adjudication/lab measures (strong) vs self-report (moderate) vs no description (poor) | **6** | Justification: Cases of Parkinson's disease (n=106, 66% male, 34% female, mean age 62.52), evaluated by specialists using standardized diagnostic criteria. Selection at specialized clinics. Systematic recruitment. Cognitive assessment (Folstein Mini-Mental State Examination). Limitations: It is not specified whether the clinical evaluators were unaware of the genotype. Convenience sample, unpaired. The criteria used to define participants as Mexican Mestizos are not mentioned. |
| **3** | **SELECTION AND COMPARABILITY OF COMPARISON GROUPS** | | Were controls appropriately defined? Were controls sampled to minimize selection bias? Was detailed description of selection procedure outlined? Were assessors of control status blinded to genotype? In multi-ethnic studies, were sub-populations (ethnicity) reported? | **5** | Justification: Healthy controls (n=216, 65% male, 35% female, mean age 63.6) were blood bank donors or spouses of patients. No family history of neurodegenerative diseases was included. Limitations: It is not specified whether the clinical evaluators were unaware of the genotype. The sample was a convenience sample, not matched. No criteria were mentioned for defining participants as Mexican Mestizos. There was a significant difference in age between the groups. The selection procedure is not described in detail. |
| **4** | **TECHNICAL CLASSIFICATION OF EXPOSURE** | | Was DNA source and storage method appropriate? Were DNA ascertainment methods similar for comparison groups? Was genotyping platform and allele-calling algorithm appropriate? Were genotyping error and call rates appropriate (≥95%)? Were genotype call rates and SNP missingness similar between groups? Was Hardy-Weinberg equilibrium tested in controls? Did authors check for outlying heterozygosity? If genotypes imputed, were methods described? | **5** | Justification: The referenced molecular techniques for DNA extraction and genotyping are described according to the manufacturer’s instructions. HW equilibrium verified in both groups. Limitations: Genotyping confirmation not mentioned. |
| **5** | **NON-TECHNICAL CLASSIFICATION OF EXPOSURE** | | Did blinded assessor conduct genotyping? Was genotyping conducted simultaneously or in batches (same methods)? If applicable, were samples randomized prior to genotyping (not all controls/cases on same plate)? | **3** | Justification: The genotyper blinding and sample randomisation are not mentioned. |
| **6** | **OTHER SOURCES OF BIAS** | | Were all sources of bias disclosed and their effect on results discussed? (selection bias, classification bias, time-lag bias, attrition bias, etc.) | **4** | Justification:Some limitations are discussed, including selection and ancestry biases. |
| **7** | **SAMPLE SIZE AND POWER** | | Was sample size appropriate? Was an a priori power analysis conducted? | **5** | Justification: N = 241 (106 cases, 135 controls), unmatched. No a priori power calculation reported. |
| **8** | **A PRIORI PLANNING OF ANALYSES** | | Was analysis plan appropriate and sufficiently described? Was selective/inappropriate reporting avoided (all test results reported)? Were tested subgroups, interactions, and sensitivity analyses described? Was statistical software identified? | **5** | Justification: Appropriate analysis: χ², Fisher’s exact test, ANOVA using SPSS v18.0. Limitation: No adjustment for multiple testing is mentioned. |
| **9** | **STATISTICAL METHODS AND CONTROL FOR CONFOUNDING** | | Were important confounders appropriately controlled? Was missing data handled appropriately (<10% missing acceptable)? Were results adjusted for multiple testing? For multi-ethnic studies, did statistical methods (e.g. PCA) control for confounding? | **5** | Justification: Confounder control is appropriate (regression correction not required)—limitation: No adjustment for multiple testing is mentioned. |
| **10** | **TESTING OF ASSUMPTIONS AND INFERENCES** | | Were all assumptions tested? Specifically: i) If haplotypes inferred, was method reported? ii) Were distant relatives/consanguinity tested? iii) Were reported sex and ethnicity checked? | **5** | Justification: HWE tested—verification of reported sex/ethnicity not mentioned. |
| **11** | **APPROPRIATENESS OF INFERENCES DRAWN** | | Were conclusions supported by results and appropriate methods? | **6** | Justification: Conclusions are appropriate and cautious; the need for further studies is acknowledged. |
|  |  | | **TOTAL SCORE** | **55** | of 77 |
| **FINAL QUALITY RATING:** | | | | **□ Good Quality** | |

| **Article title:** | | Frequency of single nucleotide polymorphisms and alpha-synuclein haplotypes associated with sporadic Parkinson's disease in the Mexican population | | | |
| --- | --- | --- | --- | --- | --- |
| **First Author:** | | Davila-Ortiz de Montellano, DJ | | | |
| **Year:** | | 2016 | | | |
| **Journal:** | | Revista de Neurología | | | |
| **Study Design:** | | Case-Control Studies | | □ Without control groups | |
|  | | | | | |
| **Item** | **Question** | | **Considerations** | **Score (1-7)** | **Notes/Comments** |
| **1** | **RATIONALE FOR STUDY** | | Was a scientific rationale for chosen genes presented to avoid selective reporting of positive results? If GWAS design (hypothesis-free approach), was rationale for selecting this design presented? | **6** | Justification: Provides information justifying the analysis of the variants and haplotypes within the context of the investigated disorder (sporadic Parkinson's disease). States the research objective. Justifies conducting the study in the Mexican population. Calculation of statistical power |
| **2** | **SELECTION AND DEFINITION OF OUTCOME** | | Were cases appropriately defined? Were participants appropriately sampled to avoid selection bias? Were case/outcome assessors blinded to genotype status? If applicable, was follow-up length appropriate and attrition rate acceptable? Outcome definitions: independent adjudication/lab measures (strong) vs self-report (moderate) vs no description (poor) | **6** | Justification: Cases of sporadic PD (n=171), evaluated by specialists using standardized diagnostic criteria. Selection at specialized clinics. From Mexico City and its metropolitan area. Participants with three generations of Mexican ancestry. Limitations: It is not specified whether the clinical evaluators were unaware of the genotype. The sample size of cases is slightly lower than the calculated 172 for the study. |
| **3** | **SELECTION AND COMPARABILITY OF COMPARISON GROUPS** | | Were controls appropriately defined? Were controls sampled to minimize selection bias? Was detailed description of selection procedure outlined? Were assessors of control status blinded to genotype? In multi-ethnic studies, were sub-populations (ethnicity) reported? | **6** | :Justification: Controls matched by sex and age (n=171). Selection procedure: no family history of PD and no data on neurological pathology from physical and neurological examination. From Mexico City and its metropolitan area. Participants had three generations of Mexican ancestry. Limitations: The sample size is slightly lower than the calculated 172 cases. |
| **4** | **TECHNICAL CLASSIFICATION OF EXPOSURE** | | Was DNA source and storage method appropriate? Were DNA ascertainment methods similar for comparison groups? Was genotyping platform and allele-calling algorithm appropriate? Were genotyping error and call rates appropriate (≥95%)? Were genotype call rates and SNP missingness similar between groups? Was Hardy-Weinberg equilibrium tested in controls? Did authors check for outlying heterozygosity? If genotypes imputed, were methods described? | **5** | Justification: The genotyping technique is described (Real-time PCR with TaqMan). HW equilibrium verified. Three SNPs were excluded due to HW equilibrium deviation (appropriate). Limitation: DNA extraction was reported as performed using conventional techniques, but no further details were provided—genotyping confirmation was not mentioned. |
| **5** | **NON-TECHNICAL CLASSIFICATION OF EXPOSURE** | | Did blinded assessor conduct genotyping? Was genotyping conducted simultaneously or in batches (same methods)? If applicable, were samples randomized prior to genotyping (not all controls/cases on same plate)? | **3** | Justification: Blinding and randomization are not mentioned. |
| **6** | **OTHER SOURCES OF BIAS** | | Were all sources of bias disclosed and their effect on results discussed? (selection bias, classification bias, time-lag bias, attrition bias, etc.) | **5** | Justification: Limitations related to missing environmental variable data are acknowledged. |
| **7** | **SAMPLE SIZE AND POWER** | | Was sample size appropriate? Was an a priori power analysis conducted? | **6** | Justification: n = 342 (171 cases, 171 controls), slightly lower than calculated. Sample size explicitly calculated using the Fleiss formula, 95% CI, 80% power. |
| **8** | **A PRIORI PLANNING OF ANALYSES** | | Was analysis plan appropriate and sufficiently described? Was selective/inappropriate reporting avoided (all test results reported)? Were tested subgroups, interactions, and sensitivity analyses described? Was statistical software identified? | **6** | Justification: Appropriate analysis: χ² with 95% CI, Hardy-Weinberg equilibrium tested. Statistical software identified (SPSS v20 and SNPstats). |
| **9** | **STATISTICAL METHODS AND CONTROL FOR CONFOUNDING** | | Were important confounders appropriately controlled? Was missing data handled appropriately (<10% missing acceptable)? Were results adjusted for multiple testing? For multi-ethnic studies, did statistical methods (e.g. PCA) control for confounding? | **5** | Justification: χ² with 95% CI. Limitation: No adjustment for multiple comparisons is mentioned. |
| **10** | **TESTING OF ASSUMPTIONS AND INFERENCES** | | Were all assumptions tested? Specifically: i) If haplotypes inferred, was method reported? ii) Were distant relatives/consanguinity tested? iii) Were reported sex and ethnicity checked? | **5** | Justification: HWE tested. Haplotype analysis was performed using appropriate software. Verification of sex, ethnicity, and consanguinity is not mentioned. |
| **11** | **APPROPRIATENESS OF INFERENCES DRAWN** | | Were conclusions supported by results and appropriate methods? | **6** | Justification: Conclusions are appropriate; the need for functional studies is acknowledged |
|  |  | | **TOTAL SCORE** | **59** | of 77 |
| **FINAL QUALITY RATING:** | | | | **□ Good Quality** | |

| **Article title:** | | Synaptotagmin XI in Parkinson's disease: new evidence from an association study in Spain and Mexico | | | |
| --- | --- | --- | --- | --- | --- |
| **First Author:** | | Sesar, A. | | | |
| **Year:** | | 2016 | | | |
| **Journal:** | | Journal of the Neurological Sciences | | | |
| **Study Design:** | | Case-Control Studies | | □ Without control groups | |
|  | | | | | |
| **Item** | **Question** | | **Considerations** | **Score (1-7)** | **Notes/Comments** |
| **1** | **RATIONALE FOR STUDY** | | Was a scientific rationale for chosen genes presented to avoid selective reporting of positive results? If GWAS design (hypothesis-free approach), was rationale for selecting this design presented? | **6** | Justification: Provides information justifying the analysis of the variant within the context of the investigated disorder (Parkinson's disease) with GWAS validation. States the research objective. Justifies conducting the study in the Mexican population. |
| **2** | **SELECTION AND DEFINITION OF OUTCOME** | | Were cases appropriately defined? Were participants appropriately sampled to avoid selection bias? Were case/outcome assessors blinded to genotype status? If applicable, was follow-up length appropriate and attrition rate acceptable? Outcome definitions: independent adjudication/lab measures (strong) vs self-report (moderate) vs no description (poor) | **6** | Justification: Two-stage design. The Discovery stage included PD cases from Galicia (n=288), diagnosed according to Gelb criteria, with Galician ancestry spanning three generations. Cases with early-onset PD, a positive family history of PD, and those positive for mutations in the LRRK2 gene were excluded. The Replication stage included sporadic PD cases (n=271), evaluated according to UK Brain Bank criteria. Participants had Mexican ancestry spanning three generations. Limitations: It is not specified whether the clinical evaluators were unaware of the genotype. |
| **3** | **SELECTION AND COMPARABILITY OF COMPARISON GROUPS** | | Were controls appropriately defined? Were controls sampled to minimize selection bias? Was detailed description of selection procedure outlined? Were assessors of control status blinded to genotype? In multi-ethnic studies, were sub-populations (ethnicity) reported? | **5** | Justification: Two-stage design. The Discovery stage included 288 controls from Galicia aged 60 or older, with no family history suggestive of neurodegenerative disorders and Galician ancestry spanning three generations. The Replication stage included 260 controls aged 40 or older from central Mexico with Mexican ancestry spanning three generations. |
| **4** | **TECHNICAL CLASSIFICATION OF EXPOSURE** | | Was DNA source and storage method appropriate? Were DNA ascertainment methods similar for comparison groups? Was genotyping platform and allele-calling algorithm appropriate? Were genotyping error and call rates appropriate (≥95%)? Were genotype call rates and SNP missingness similar between groups? Was Hardy-Weinberg equilibrium tested in controls? Did authors check for outlying heterozygosity? If genotypes imputed, were methods described? | **7** | They mention the DNA extraction method used (Wizard® Genomic DNA Purification Kit). They mention the platforms used for genotyping: Discovery Phase (SNPlex) and Replication Phase (MassArray Sequenom®). Robust methodology. Samples with a call rate <0.80, SNPs with a call rate <0.95, and those deviating from the Hardy-Weinberg (HW) equilibrium in the controls (p < 0.01) were eliminated. For imputation of additional variants, IMPUTE v2.2 and a multipopulation reference panel were used. |
| **5** | **NON-TECHNICAL CLASSIFICATION OF EXPOSURE** | | Did blinded assessor conduct genotyping? Was genotyping conducted simultaneously or in batches (same methods)? If applicable, were samples randomized prior to genotyping (not all controls/cases on same plate)? | **3** | Justification: Blinding and randomization are not mentioned. |
| **6** | **OTHER SOURCES OF BIAS** | | Were all sources of bias disclosed and their effect on results discussed? (selection bias, classification bias, time-lag bias, attrition bias, etc.) | **6** | Justification: Critical analysis. They discuss possible stratification in a Mexican sample, differences in Linkage Disequilibrium (LD) between populations. |
| **7** | **SAMPLE SIZE AND POWER** | | Was sample size appropriate? Was an a priori power analysis conducted? | **6** | Justification: Appropriate size for a two-stage design. Discovery: 268 cases, 265 controls (Galicia). Replication: 271 cases, 260 controls (Mexico). Power 80% for OR≥1.7. |
| **8** | **A PRIORI PLANNING OF ANALYSES** | | Was analysis plan appropriate and sufficiently described? Was selective/inappropriate reporting avoided (all test results reported)? Were tested subgroups, interactions, and sensitivity analyses described? Was statistical software identified? | **7** | Justification: Complete bioinformatic analysis. Statistical power (Quanto v.1.2.4 software). Association analysis by logistic regression under a log-additive model (SNPassoc, R package), statistical significance with likelihood ratio test, with correction for multiple testing (q-value R package). Linkage disequilibrium (LD) and haplotype analysis (Haploview |
| **9** | **STATISTICAL METHODS AND CONTROL FOR CONFOUNDING** | | Were important confounders appropriately controlled? Was missing data handled appropriately (<10% missing acceptable)? Were results adjusted for multiple testing? For multi-ethnic studies, did statistical methods (e.g. PCA) control for confounding? | **6** | Justification: Logistic regression, log-additive model. FDR correction. They appropriately discuss the correction in LD. |
| **10** | **TESTING OF ASSUMPTIONS AND INFERENCES** | | Were all assumptions tested? Specifically: i) If haplotypes inferred, was method reported? ii) Were distant relatives/consanguinity tested? iii) Were reported sex and ethnicity checked? | **7** | Justification: Imputation with multi-population panel, LD analysis with 1000G, haplotype analysis. In silico analysis. |
| **11** | **APPROPRIATENESS OF INFERENCES DRAWN** | | Were conclusions supported by results and appropriate methods? | **6** | Justification: Appropriate conclusions |
|  |  | | **TOTAL SCORE** | **65** | of 77 |
| **FINAL QUALITY RATING:** | | | | **□ Good Quality** | |

| **Article title:** | | Analysis of the rs13306560 functional variant in the promoter region of the MTHFR gene in sporadic Parkinsons disease | | | |
| --- | --- | --- | --- | --- | --- |
| **First Author:** | | Garcia, S. | | | |
| **Year:** | | 2017 | | | |
| **Journal:** | | Neuro Endocrinology Letters | | | |
| **Study Design:** | | Case-Control Studies | | □ Without control groups | |
|  | | | | | |
| **Item** | **Question** | | **Considerations** | **Score (1-7)** | **Notes/Comments** |
| **1** | **RATIONALE FOR STUDY** | | Was a scientific rationale for chosen genes presented to avoid selective reporting of positive results? If GWAS design (hypothesis-free approach), was rationale for selecting this design presented? | **6** | Justification: Provides information justifying the investigation of a regulatory variant in a candidate gene for susceptibility to the investigated disorder (identified by GWAS). States the objective of the research. Justifies conducting the study in the Mexican population. Limitations: Lacks analysis of the biological mechanism of the investigated variant. |
| **2** | **SELECTION AND DEFINITION OF OUTCOME** | | Were cases appropriately defined? Were participants appropriately sampled to avoid selection bias? Were case/outcome assessors blinded to genotype status? If applicable, was follow-up length appropriate and attrition rate acceptable? Outcome definitions: independent adjudication/lab measures (strong) vs self-report (moderate) vs no description (poor) | **6** | Justification: Cases of sporadic Parkinson's disease (n=113), evaluated using standardized diagnostic criteria. Selection was conducted at specialized clinics. Systematic recruitment was used. Participants were of Mexican mestizo descent. Limitations: It is not specified whether the clinical evaluators were unaware of the genotype. Criteria for defining Mexican mestizo descent are not specified. |
| **3** | **SELECTION AND COMPARABILITY OF COMPARISON GROUPS** | | Were controls appropriately defined? Were controls sampled to minimize selection bias? Was detailed description of selection procedure outlined? Were assessors of control status blinded to genotype? In multi-ethnic studies, were sub-populations (ethnicity) reported? | **5** | Justification: The controls were donors or spouses of neurologically healthy patients (n=124). They were Mexican mestizos, with an average age older than the cases. Limitations: It is not specified whether the clinical evaluators were unaware of the genotype. The sample was unpaired, and significant sex differences were observed. The criteria used to define participants as Mexican mestizos are not mentioned. |
| **4** | **TECHNICAL CLASSIFICATION OF EXPOSURE** | | Was DNA source and storage method appropriate? Were DNA ascertainment methods similar for comparison groups? Was genotyping platform and allele-calling algorithm appropriate? Were genotyping error and call rates appropriate (≥95%)? Were genotype call rates and SNP missingness similar between groups? Was Hardy-Weinberg equilibrium tested in controls? Did authors check for outlying heterozygosity? If genotypes imputed, were methods described? | **5** | Justification: Describes the method of obtaining DNA (peripheral blood samples by the DTAB/CTAB). Genotyping was performed by real-time PCR using TaqMan probes. The HW equilibrium was verified. Limitations: The genotype-calling rate and randomisation are not mentioned. Prior to screening for PARK genes, could be an introduction of selection bias. |
| **5** | **NON-TECHNICAL CLASSIFICATION OF EXPOSURE** | | Did blinded assessor conduct genotyping? Was genotyping conducted simultaneously or in batches (same methods)? If applicable, were samples randomized prior to genotyping (not all controls/cases on same plate)? | **3** | Justification: Blinding and randomization not mentioned. |
| **6** | **OTHER SOURCES OF BIAS** | | Were all sources of bias disclosed and their effect on results discussed? (selection bias, classification bias, time-lag bias, attrition bias, etc.) | **6** | Justification: They discuss limitations. For example, they acknowledge that in patients with Parkinson's disease, treatment can alter plasma homocysteine ​​levels. They also note the lack of serum MTHFR determination. They report that larger sample studies are needed to confirm their findings. |
| **7** | **SAMPLE SIZE AND POWER** | | Was sample size appropriate? Was an a priori power analysis conducted? | **4** | Justification: Small sample size due to the variant is low MAF. N=237 (113 cases, 124 controls). |
| **8** | **A PRIORI PLANNING OF ANALYSES** | | Was analysis plan appropriate and sufficiently described? Was selective/inappropriate reporting avoided (all test results reported)? Were tested subgroups, interactions, and sensitivity analyses described? Was statistical software identified? | **5** | Justification: Analysis, χ² test, logistic regression, odds ratios. Hardy-Weinberg equilibrium (HWE). Indicated as the statistical analysis software (SPSS v 18.0) |
| **9** | **STATISTICAL METHODS AND CONTROL FOR CONFOUNDING** | | Were important confounders appropriately controlled? Was missing data handled appropriately (<10% missing acceptable)? Were results adjusted for multiple testing? For multi-ethnic studies, did statistical methods (e.g. PCA) control for confounding? | **4** | Justification: Logistic regression adjusted for sex. Limitations: Age adjustment is not shown despite significant differences. |
| **10** | **TESTING OF ASSUMPTIONS AND INFERENCES** | | Were all assumptions tested? Specifically: i) If haplotypes inferred, was method reported? ii) Were distant relatives/consanguinity tested? iii) Were reported sex and ethnicity checked? | **4** | Justification: HW equilibrium proven. They do not dispute imputation, nor the verification of ancestry. |
| **11** | **APPROPRIATENESS OF INFERENCES DRAWN** | | Were conclusions supported by results and appropriate methods? | **4** | Justification: Potential bias due to overinterpretation of data, which are not significant after adjustment (p=0.058). |
|  |  | | **TOTAL SCORE** | **52** | of 77 |
| **FINAL QUALITY RATING:** | | | | **□ Good Quality** | |

| **Article title:** | | Association of polymorphisms and reduced expression levels of the NR4A2 gene with Parkinson's disease in a Mexican population | | | |
| --- | --- | --- | --- | --- | --- |
| **First Author:** | | Ruiz-Sanchez, E | | | |
| **Year:** | | 2017 | | | |
| **Journal:** | | Journal of the Neurological Sciences | | | |
| **Study Design:** | | Case-Control Studies | | □ Without control groups | |
|  | | | | | |
| **Item** | **Question** | | **Considerations** | **Score (1-7)** | **Notes/Comments** |
| **1** | **RATIONALE FOR STUDY** | | Was a scientific rationale for chosen genes presented to avoid selective reporting of positive results? If GWAS design (hypothesis-free approach), was rationale for selecting this design presented? | **7** | Justification: It provides a very comprehensive conceptual framework that justifies the analysis of the variants examined in the context of the disorder under investigation (Parkinson's disease). It states the research objective and justifies conducting the study in the Mexican population. |
| **2** | **SELECTION AND DEFINITION OF OUTCOME** | | Were cases appropriately defined? Were participants appropriately sampled to avoid selection bias? Were case/outcome assessors blinded to genotype status? If applicable, was follow-up length appropriate and attrition rate acceptable? Outcome definitions: independent adjudication/lab measures (strong) vs self-report (moderate) vs no description (poor) | **6** | Justification: Casos de enfermedad de Parkinson esporádica (n=227), evaluados por especialistas mediante criterios diagnósticos estandarizados. Selección en clínicas especializadas. Participantes de ascendencia mexicana, pertenecientes a tres generaciones, con apellidos de origen español. Limitaciones: No se especifica si los evaluadores clínicos desconocían el genotipo. |
| **3** | **SELECTION AND COMPARABILITY OF COMPARISON GROUPS** | | Were controls appropriately defined? Were controls sampled to minimize selection bias? Was detailed description of selection procedure outlined? Were assessors of control status blinded to genotype? In multi-ethnic studies, were sub-populations (ethnicity) reported? | **7** | Justification: Controls (n=227) matched (1 case for every 2 controls) by age and sex. Matching by frequency and individual. No diagnosis of neurodegenerative disease, no family history of movement disorders, and no uncontrolled severe physical illness. Participants of Mexican descent, belonging to three generations, with surnames of Spanish origin. It is not specified whether the clinical evaluators were unaware of the genotype. |
| **4** | **TECHNICAL CLASSIFICATION OF EXPOSURE** | | Was DNA source and storage method appropriate? Were DNA ascertainment methods similar for comparison groups? Was genotyping platform and allele-calling algorithm appropriate? Were genotyping error and call rates appropriate (≥95%)? Were genotype call rates and SNP missingness similar between groups? Was Hardy-Weinberg equilibrium tested in controls? Did authors check for outlying heterozygosity? If genotypes imputed, were methods described? | **7** | Justification: Indicates the methodology for DNA extraction using standardized methods. Describes the methods for selecting the analyzed genetic variants. Indicates the methodology for genotyping (HRM), with sequencing validation of 20% of random samples with 99% concordance. Additionally, NR4A2 mRNA levels were evaluated. |
| **5** | **NON-TECHNICAL CLASSIFICATION OF EXPOSURE** | | Did blinded assessor conduct genotyping? Was genotyping conducted simultaneously or in batches (same methods)? If applicable, were samples randomized prior to genotyping (not all controls/cases on same plate)? | **3** | Justification: Blinding and randomization are not mentioned. |
| **6** | **OTHER SOURCES OF BIAS** | | Were all sources of bias disclosed and their effect on results discussed? (selection bias, classification bias, time-lag bias, attrition bias, etc.) | **6** | Justification: They discuss limitations, including the lack of protein-level measurement. Number of samples analyzed for expression analysis (n=156 patients, 102 controls). No AIM assessment. |
| **7** | **SAMPLE SIZE AND POWER** | | Was sample size appropriate? Was an a priori power analysis conducted? | **6** | Justification: Indicates the calculation of statistical power calculated a posteriori: 28% for rs34884856 (insufficient) and 93% for rs35479735 (appropriate). |
| **8** | **A PRIORI PLANNING OF ANALYSES** | | Was analysis plan appropriate and sufficiently described? Was selective/inappropriate reporting avoided (all test results reported)? Were tested subgroups, interactions, and sensitivity analyses described? Was statistical software identified? | **7** | Justification: Robust statistical analysis. Bivariate and multivariate logistic regression. Multiple inheritance models. Goodness-of-fit tests. Post-hoc statistical power calculations indicate the software used (SPSS v.12.0, STATA v.11, QUANTO Haploview version 4.1). |
| **9** | **STATISTICAL METHODS AND CONTROL FOR CONFOUNDING** | | Were important confounders appropriately controlled? Was missing data handled appropriately (<10% missing acceptable)? Were results adjusted for multiple testing? For multi-ethnic studies, did statistical methods (e.g. PCA) control for confounding? | **7** | Justification: Robust statistical analysis. Tests: Hardy-Weinberg equilibrium using chi-square. Bivariate and multivariate logistic regression (constructed considering relevant biological variables and/or those with statistical significance). Multiple inheritance models were tested (dominant, recessive, heterozygous). Goodness-of-fit tests (Hosmer-Lemeshow) were used for model selection, and odds ratios (ORs) were reported. Pairwise linkage disequilibrium (LD, D') was performed. Statistical power to detect an association was performed. Correlations between gene expression levels, genotype, and inheritance models were tested using the Kruskal-Wallis or Mann-Whitney U test. |
| **10** | **TESTING OF ASSUMPTIONS AND INFERENCES** | | Were all assumptions tested? Specifically: i) If haplotypes inferred, was method reported? ii) Were distant relatives/consanguinity tested? iii) Were reported sex and ethnicity checked? | **7** | Justification: Good analysis of assumptions. |
| **11** | **APPROPRIATENESS OF INFERENCES DRAWN** | | Were conclusions supported by results and appropriate methods? | **6** | Justification: Appropriate conclusions. Acknowledging the need for functional studies. They address the discussion of genotype-phenotype discrepancy and propose epigenetic mechanisms. |
|  |  | | **TOTAL SCORE** | **69** | of 77 |
| **FINAL QUALITY RATING:** | | | | **□ Good Quality** | |

| **Article title:** | | Association of mitochondrial variants A4336G of the tRNAGln gene and 8701G/A of the MT-ATP6 gene in Mexicans Mestizos with Parkinson disease | | | |
| --- | --- | --- | --- | --- | --- |
| **First Author:** | | Garcia, S | | | |
| **Year:** | | 2019 | | | |
| **Journal:** | | Folia Neuropathologica | | | |
| **Study Design:** | | Case-Control Studies | | □ Without control groups | |
|  | | | | | |
| **Item** | **Question** | | **Considerations** | **Score (1-7)** | **Notes/Comments** |
| **1** | **RATIONALE FOR STUDY** | | Was a scientific rationale for chosen genes presented to avoid selective reporting of positive results? If GWAS design (hypothesis-free approach), was rationale for selecting this design presented? | **6** | Justification: Provides information justifying the analysis of the variant within the context of the investigated disorder (Parkinson's disease). States the research objective. Justifies conducting the study in the Mexican population. Limitations: Acknowledges inconsistencies in previous associations regarding one of the analyzed variants. |
| **2** | **SELECTION AND DEFINITION OF OUTCOME** | | Were cases appropriately defined? Were participants appropriately sampled to avoid selection bias? Were case/outcome assessors blinded to genotype status? If applicable, was follow-up length appropriate and attrition rate acceptable? Outcome definitions: independent adjudication/lab measures (strong) vs self-report (moderate) vs no description (poor) | **6** | Justification: Parkinson's disease cases (n=175), evaluated by specialists using standardized diagnostic criteria. Sequential recruitment at specialized clinics by specialists. Participants of Mexican ethnic origin. Exclusion of secondary PD cases. Limitations: Convenience sample. It is not specified whether the clinical evaluators were unaware of the genotype. |
| **3** | **SELECTION AND COMPARABILITY OF COMPARISON GROUPS** | | Were controls appropriately defined? Were controls sampled to minimize selection bias? Was detailed description of selection procedure outlined? Were assessors of control status blinded to genotype? In multi-ethnic studies, were sub-populations (ethnicity) reported? | **5** | Justification: Controls (n=194) with no individual or family history of PD or neurodegenerative diseases. With a higher average age than the cases. Participants of Mexican ethnic origin. Limitations: The sample was unpaired and showed significant sex differences. It is not specified whether the clinical evaluators were unaware of the genotype. The criteria for defining ethnicity are not specified. |
| **4** | **TECHNICAL CLASSIFICATION OF EXPOSURE** | | Was DNA source and storage method appropriate? Were DNA ascertainment methods similar for comparison groups? Was genotyping platform and allele-calling algorithm appropriate? Were genotyping error and call rates appropriate (≥95%)? Were genotype call rates and SNP missingness similar between groups? Was Hardy-Weinberg equilibrium tested in controls? Did authors check for outlying heterozygosity? If genotypes imputed, were methods described? | **6** | Justification: Describes the DNA collection method (peripheral blood samples using DTAB/CTAB). Genotyping was performed using real-time PCR with TaqMan probes. Confirmation was obtained by sequencing. Limitations: The genotyping rate and randomization are not mentioned. It is not explained why the Hardy-Weinberg equilibrium (HWE) verification of mtDNA was not performed. |
| **5** | **NON-TECHNICAL CLASSIFICATION OF EXPOSURE** | | Did blinded assessor conduct genotyping? Was genotyping conducted simultaneously or in batches (same methods)? If applicable, were samples randomized prior to genotyping (not all controls/cases on same plate)? | **4** | Justification: Blinding and randomization were not mentioned.Indicates confirmation by sequencing |
| **6** | **OTHER SOURCES OF BIAS** | | Were all sources of bias disclosed and their effect on results discussed? (selection bias, classification bias, time-lag bias, attrition bias, etc.) | **4** | Justification: Acknowledges some limitations, such as rare variants in the studied population, and mentions the need to study other genes. Discusses population heterogeneity (mixed ancestry). Limitations: Does not discuss significant differences in sex or age, and does not adjust for significant variables such as coffee consumption. |
| **7** | **SAMPLE SIZE AND POWER** | | Was sample size appropriate? Was an a priori power analysis conducted? | **4** | Justification: Does not report power calculation—unpaired convenience sample. For one of the variants, 194 controls and 175 cases are reported, and for the second, 21 controls and 154 cases are reported, without discussing these differences. |
| **8** | **A PRIORI PLANNING OF ANALYSES** | | Was analysis plan appropriate and sufficiently described? Was selective/inappropriate reporting avoided (all test results reported)? Were tested subgroups, interactions, and sensitivity analyses described? Was statistical software identified? | **3** | Justification: Basic analysis plan (Mann-Whitney U test, Student's t-test, χ², OR, and 95% CI). Limitations: Does not perform multivariate regression analysis. Does not correct for multiple comparisons. The difference in the number of samples is not explained. |
| **9** | **STATISTICAL METHODS AND CONTROL FOR CONFOUNDING** | | Were important confounders appropriately controlled? Was missing data handled appropriately (<10% missing acceptable)? Were results adjusted for multiple testing? For multi-ethnic studies, did statistical methods (e.g. PCA) control for confounding? | **3** | Justification: Basic analysis plan. Limitations: The sample has significant differences in age and sex, so adjustments for these factors were necessary. |
| **10** | **TESTING OF ASSUMPTIONS AND INFERENCES** | | Were all assumptions tested? Specifically: i) If haplotypes inferred, was method reported? ii) Were distant relatives/consanguinity tested? iii) Were reported sex and ethnicity checked? | **4** | Justification: Failure to verify heteroplasmy (this is important when analyzing mtDNA). |
| **11** | **APPROPRIATENESS OF INFERENCES DRAWN** | | Were conclusions supported by results and appropriate methods? | **6** | Justification: Appropriate conclusions. Acknowledges sample size limitations. Proposes future studies on other genes. Limitations: Does not extensively discuss age/sex differences. Does not mention the limitation of not adjusting for confounders. |
|  |  | | **TOTAL SCORE** | **51** | of 77 |
| **FINAL QUALITY RATING:** | | | | **□ Good Quality** | |

| **Article title:** | | H1/H2 MAPT haplotype and Parkinson's disease in Mexican mestizo population | | | |
| --- | --- | --- | --- | --- | --- |
| **First Author:** | | Miranda-Morales, EG | | | |
| **Year:** | | 2019 | | | |
| **Journal:** | | Neuroscience Letters | | | |
| **Study Design:** | | Case-Control Studies | | □ Without control groups | |
|  | | | | | |
| **Item** | **Question** | | **Considerations** | **Score (1-7)** | **Notes/Comments** |
| **1** | **RATIONALE FOR STUDY** | | Was a scientific rationale for chosen genes presented to avoid selective reporting of positive results? If GWAS design (hypothesis-free approach), was rationale for selecting this design presented? | **6** | Justification: Provides information justifying the analysis of the investigated haplotypes in the context of the investigated disorder (sporadic Parkinson's disease). States the research objective. Justifies conducting the study in the Mexican population. |
| **2** | **SELECTION AND DEFINITION OF OUTCOME** | | Were cases appropriately defined? Were participants appropriately sampled to avoid selection bias? Were case/outcome assessors blinded to genotype status? If applicable, was follow-up length appropriate and attrition rate acceptable? Outcome definitions: independent adjudication/lab measures (strong) vs self-report (moderate) vs no description (poor) | **6** | Justification: 108 cases of late-onset Parkinson's disease (age of onset greater than 50 years), evaluated using standardized diagnostic criteria. Recruitment was conducted at specialised clinics—limitations: a matched convenience sample. No specific information on ancestry is provided. It is not specified whether the clinical evaluators were blinded to the genotype. |
| **3** | **SELECTION AND COMPARABILITY OF COMPARISON GROUPS** | | Were controls appropriately defined? Were controls sampled to minimize selection bias? Was detailed description of selection procedure outlined? Were assessors of control status blinded to genotype? In multi-ethnic studies, were sub-populations (ethnicity) reported? | **4** | Justification: Controls (n=108) matched for age, sex, gender, and region. Limitations: Selection criteria (inclusion/exclusion) are not described. It is not specified whether the clinical evaluators were blinded to the genotype. The criteria for defining ethnicity are not specified. |
| **4** | **TECHNICAL CLASSIFICATION OF EXPOSURE** | | Was DNA source and storage method appropriate? Were DNA ascertainment methods similar for comparison groups? Was genotyping platform and allele-calling algorithm appropriate? Were genotyping error and call rates appropriate (≥95%)? Were genotype call rates and SNP missingness similar between groups? Was Hardy-Weinberg equilibrium tested in controls? Did authors check for outlying heterozygosity? If genotypes imputed, were methods described? | **6** | Justification: Describes the method for obtaining DNA (peripheral blood samples using the QIAamp DNA Blood Mini Kit®). Describes the haplotype identification technique (endpoint PCR and electrophoresis for deletion identification). Does not mention haplotype confirmation. |
| **5** | **NON-TECHNICAL CLASSIFICATION OF EXPOSURE** | | Did blinded assessor conduct genotyping? Was genotyping conducted simultaneously or in batches (same methods)? If applicable, were samples randomized prior to genotyping (not all controls/cases on same plate)? | **3** | Justification: Blinding and randomization are not mentioned. |
| **6** | **OTHER SOURCES OF BIAS** | | Were all sources of bias disclosed and their effect on results discussed? (selection bias, classification bias, time-lag bias, attrition bias, etc.) | **5** | Justification: Limitations, such as limited sampling in the southern Mexican population, are acknowledged. H1 sub-haplotypes were not determined. |
| **7** | **SAMPLE SIZE AND POWER** | | Was sample size appropriate? Was an a priori power analysis conducted? | **6** | Justification: Paired convenience sample. Power calculation not reported. |
| **8** | **A PRIORI PLANNING OF ANALYSES** | | Was analysis plan appropriate and sufficiently described? Was selective/inappropriate reporting avoided (all test results reported)? Were tested subgroups, interactions, and sensitivity analyses described? Was statistical software identified? | **5** | Justification: Compares allele frequencies with stratification by region and by familial/sporadic PD. Indicates software programs used (SNPstats and SPSS). Limitations: Does not perform multivariate regression analysis. Does not correct for multiple comparisons. |
| **9** | **STATISTICAL METHODS AND CONTROL FOR CONFOUNDING** | | Were important confounders appropriately controlled? Was missing data handled appropriately (<10% missing acceptable)? Were results adjusted for multiple testing? For multi-ethnic studies, did statistical methods (e.g. PCA) control for confounding? | **5** | Justification: Compares allele frequencies with stratification by region and by familial/sporadic PD. Limitations: Does not perform multivariate regression analysis or adjustments for multiple comparisons. Does not perform linkage disequilibrium analysis. |
| **10** | **TESTING OF ASSUMPTIONS AND INFERENCES** | | Were all assumptions tested? Specifically: i) If haplotypes inferred, was method reported? ii) Were distant relatives/consanguinity tested? iii) Were reported sex and ethnicity checked? | **4** | Justification: Acknowledges some limitations. Limitations: Does not include AIMs; only stratifies by region. Does not perform linkage disequilibrium analysis. |
| **11** | **APPROPRIATENESS OF INFERENCES DRAWN** | | Were conclusions supported by results and appropriate methods? | **6** | Justification: Appropriate conclusions. Proposes future studies. Acknowledges limitations. |
|  |  | | **TOTAL SCORE** | **56** | of 77 |
| **FINAL QUALITY RATING:** | | | | **□ Good Quality** | |

| **Article title:** | | rs3764435 Associated with Parkinson's disease in Mexican Mestizos: Case-control study reveals protective effects against disease development and cognitive impairment | | | |
| --- | --- | --- | --- | --- | --- |
| **First Author:** | | Salas-Leal, AC | | | |
| **Year:** | | 2019 | | | |
| **Journal:** | | Frontiers in Neurology | | | |
| **Study Design:** | | Case-Control Studies | | □ Without control groups | |
|  | | | | | |
| **Item** | **Question** | | **Considerations** | **Score (1-7)** | **Notes/Comments** |
| **1** | **RATIONALE FOR STUDY** | | Was a scientific rationale for chosen genes presented to avoid selective reporting of positive results? If GWAS design (hypothesis-free approach), was rationale for selecting this design presented? | **6** | Justification: Provides information justifying the analysis of the variant within the context of the investigated disorder (sporadic Parkinson's disease). States the research objective. Justifies conducting the study in the Mexican population. |
| **2** | **SELECTION AND DEFINITION OF OUTCOME** | | Were cases appropriately defined? Were participants appropriately sampled to avoid selection bias? Were case/outcome assessors blinded to genotype status? If applicable, was follow-up length appropriate and attrition rate acceptable? Outcome definitions: independent adjudication/lab measures (strong) vs self-report (moderate) vs no description (poor) | **6** | Justificación: 120 casos de enfermedad de Parkinson, evaluados por criterios diagnósticos estandarizados por especialistas. Reclutamiento en clínicas especializadas. Evaluaciones adicionales, cognitivas (por MMSE) y de depresión ( por HDRS). Limitaciones: Muestra de conveniencia no pareada. No se proporciona información específica sobre la ascendencia. No se especifica si los evaluadores clínicos desconocían el genotipo. |
| **3** | **SELECTION AND COMPARABILITY OF COMPARISON GROUPS** | | Were controls appropriately defined? Were controls sampled to minimize selection bias? Was detailed description of selection procedure outlined? Were assessors of control status blinded to genotype? In multi-ethnic studies, were sub-populations (ethnicity) reported? | **4** | Justificación: Controls (n=190) were an unmatched sample with no differences by age or sex. Additional assessments included cognitive function (MMSE) and depression (HDRS). Limitations: Selection criteria (inclusion/exclusion) are not described. It is not specified whether the clinical evaluators were blinded to the genotype. The criteria for defining ethnicity are not specified. |
| **4** | **TECHNICAL CLASSIFICATION OF EXPOSURE** | | Was DNA source and storage method appropriate? Were DNA ascertainment methods similar for comparison groups? Was genotyping platform and allele-calling algorithm appropriate? Were genotyping error and call rates appropriate (≥95%)? Were genotype call rates and SNP missingness similar between groups? Was Hardy-Weinberg equilibrium tested in controls? Did authors check for outlying heterozygosity? If genotypes imputed, were methods described? | **6** | Justification: Describes the methodology for obtaining DNA. Describes the methodology used for SNP genotyping (real-time PCR with TaqMan assay). Quality controls, such as genotyping replication, error rate, and the use of quality controls, are not mentioned. |
| **5** | **NON-TECHNICAL CLASSIFICATION OF EXPOSURE** | | Did blinded assessor conduct genotyping? Was genotyping conducted simultaneously or in batches (same methods)? If applicable, were samples randomized prior to genotyping (not all controls/cases on same plate)? | **3** | Justification: Blinding and randomization not mentioned. |
| **6** | **OTHER SOURCES OF BIAS** | | Were all sources of bias disclosed and their effect on results discussed? (selection bias, classification bias, time-lag bias, attrition bias, etc.) | **6** | Justification: Addresses limitations such as a small sample size, limited sampling in some geographic regions, the lack of inclusion of ancestry-informative markers (AIMs), and the lack of enzyme activity determinations and gene expression analysis. |
| **7** | **SAMPLE SIZE AND POWER** | | Was sample size appropriate? Was an a priori power analysis conducted? | **6** | Justification: Unpaired convenience sample with no significant differences in age or sex. Indicates the calculation of the post-hoc statistical power. |
| **8** | **A PRIORI PLANNING OF ANALYSES** | | Was analysis plan appropriate and sufficiently described? Was selective/inappropriate reporting avoided (all test results reported)? Were tested subgroups, interactions, and sensitivity analyses described? Was statistical software identified? | **6** | Justification: Appropriate analysis: χ² and Pearson tests. Hardy-Weinberg equilibrium was evaluated. Odds ratios and 95% confidence intervals are reported. Inheritance models (codominant, dominant, recessive) were analyzed. Binary logistic regression was used. Measures of central tendency and Student's t-tests were used (to compare continuous variables). The software used (Stata) is indicated. Limitations: No adjustment for multiple testing was mentioned. |
| **9** | **STATISTICAL METHODS AND CONTROL FOR CONFOUNDING** | | Were important confounders appropriately controlled? Was missing data handled appropriately (<10% missing acceptable)? Were results adjusted for multiple testing? For multi-ethnic studies, did statistical methods (e.g. PCA) control for confounding? | **5** | Justification: The analysis was performed with limited adjustments (age, sex, environmental, and lifestyle factors). Adjustment for a significant variable (Depression) and adjustment for multiple comparisons were not considered. |
| **10** | **TESTING OF ASSUMPTIONS AND INFERENCES** | | Were all assumptions tested? Specifically: i) If haplotypes inferred, was method reported? ii) Were distant relatives/consanguinity tested? iii) Were reported sex and ethnicity checked? | **5** | Justification: Some limitations are acknowledged. Limitations: Possible uncontrolled population stratification; does not include AIM. It is noted that the analysed variant, along with others, forms a haplotype that may regulate gene expression; however, this is not further explored. The work could be enriched with an in silico functional analysis. |
| **11** | **APPROPRIATENESS OF INFERENCES DRAWN** | | Were conclusions supported by results and appropriate methods? | **5** | Justification: They acknowledge the need for replication and functional studies; therefore, their conclusion may be overinterpreted in some respects. |
|  |  | | **TOTAL SCORE** | **58** | of 77 |
| **FINAL QUALITY RATING:** | | | | **□ Good Quality** | |

| **Article title:** | | Differences in MTHFR and LRRK2 variant's association with sporadic Parkinson's disease in Mexican Mestizos correlated to Native American ancestry | | | |
| --- | --- | --- | --- | --- | --- |
| **First Author:** | | Romero-Gutierrez, E | | | |
| **Year:** | | 2021 | | | |
| **Journal:** | | npj Parkinson's Disease | | | |
| **Study Design:** | | Case-Control Studies | | □ Without control groups | |
|  | | | | | |
| **Item** | **Question** | | **Considerations** | **Score (1-7)** | **Notes/Comments** |
| **1** | **RATIONALE FOR STUDY** | | Was a scientific rationale for chosen genes presented to avoid selective reporting of positive results? If GWAS design (hypothesis-free approach), was rationale for selecting this design presented? | **7** | Justification: Provides information justifying the analysis of the variants studied in the context of the investigated disorder (sporadic Parkinson's disease). States the research objective. Justifies conducting the study in the Mexican population. Theoretical framework on ancestry and population structure |
| **2** | **SELECTION AND DEFINITION OF OUTCOME** | | Were cases appropriately defined? Were participants appropriately sampled to avoid selection bias? Were case/outcome assessors blinded to genotype status? If applicable, was follow-up length appropriate and attrition rate acceptable? Outcome definitions: independent adjudication/lab measures (strong) vs self-report (moderate) vs no description (poor) | **6** | Justification: PD cases (n=118), evaluated by specialists using standardized diagnostic criteria, with no positive family history of PD. Systematic recruitment. Selection in specialized clinics. Additional assessments: cognitive (MMSE) and depression (HDRS). Participants of Mexican ancestry across three generations and ancestry markers. Limitations: It is not specified whether the clinical evaluators were unaware of the genotype—unpaired convenience sample. |
| **3** | **SELECTION AND COMPARABILITY OF COMPARISON GROUPS** | | Were controls appropriately defined? Were controls sampled to minimize selection bias? Was detailed description of selection procedure outlined? Were assessors of control status blinded to genotype? In multi-ethnic studies, were sub-populations (ethnicity) reported? | **6** | Justification: Controls (n=193) showed no differences by age or sex. No family or personal history of neurodegenerative diseases. Additional assessments: cognitive (MMSE) and depression (HDRS). Participants of Mexican ancestry across three generations and ancestry markers. Limitations: It is not specified whether the clinical evaluators were unaware of the genotype—unpaired convenience sample. |
| **4** | **TECHNICAL CLASSIFICATION OF EXPOSURE** | | Was DNA source and storage method appropriate? Were DNA ascertainment methods similar for comparison groups? Was genotyping platform and allele-calling algorithm appropriate? Were genotyping error and call rates appropriate (≥95%)? Were genotype call rates and SNP missingness similar between groups? Was Hardy-Weinberg equilibrium tested in controls? Did authors check for outlying heterozygosity? If genotypes imputed, were methods described? | **7** | Justification: Describes the method of obtaining DNA (peripheral blood samples using the QIAamp DNA extraction kit), quality verification (purity and concentration), and storage. Describes methods for SNP selection. Genotyping was performed by real-time PCR using TaqMan probes with genotyping replication in 10% of samples. Hardy-Weinberg equilibrium was verified. Includes biochemical determinations (total cholesterol, uric acid, and glucose levels). Uses a validated panel of 32 AIMs designed for Mexican individuals. |
| **5** | **NON-TECHNICAL CLASSIFICATION OF EXPOSURE** | | Did blinded assessor conduct genotyping? Was genotyping conducted simultaneously or in batches (same methods)? If applicable, were samples randomized prior to genotyping (not all controls/cases on same plate)? | **3** | Justification: Blinding and randomization not mentioned. |
| **6** | **OTHER SOURCES OF BIAS** | | Were all sources of bias disclosed and their effect on results discussed? (selection bias, classification bias, time-lag bias, attrition bias, etc.) | **6** | Justification: Limitations are acknowledged. Sample size limitations are acknowledged. Geographic recruitment bias is discussed. The absence of functional analysis is mentioned. The need for replication is discussed. |
| **7** | **SAMPLE SIZE AND POWER** | | Was sample size appropriate? Was an a priori power analysis conducted? | **5** | Justification: Unpaired convenience sample with no significant differences in age or sex. The limitation of statistical power is acknowledged. Limitations: Power calculations are not reported a priori. Stratification by ancestry reduces statistical power. |
| **8** | **A PRIORI PLANNING OF ANALYSES** | | Was analysis plan appropriate and sufficiently described? Was selective/inappropriate reporting avoided (all test results reported)? Were tested subgroups, interactions, and sensitivity analyses described? Was statistical software identified? | **7** | Justification: The analysis plan is detailed. Indicates the SNP selection criteria. The analysis considers population ancestry. Performs corrections for multiple comparisons. The software programs used are indicated (SPSS, Haploview, ADMIXTURE, STATA). |
| **9** | **STATISTICAL METHODS AND CONTROL FOR CONFOUNDING** | | Were important confounders appropriately controlled? Was missing data handled appropriately (<10% missing acceptable)? Were results adjusted for multiple testing? For multi-ethnic studies, did statistical methods (e.g. PCA) control for confounding? | **7** | Justification: Appropriate analysis: Adjustments for age, sex, and ancestry are made, corrections for multiple comparisons are applied, multiple genetic models are tested, and a principal component analysis is performed. In the meta-analysis (with fixed- and random-effects models), heterogeneity and publication bias are assessed. |
| **10** | **TESTING OF ASSUMPTIONS AND INFERENCES** | | Were all assumptions tested? Specifically: i) If haplotypes inferred, was method reported? ii) Were distant relatives/consanguinity tested? iii) Were reported sex and ethnicity checked? | **7** | Justification: The assumptions were thoroughly analyzed. Hardy-Weinberg equilibrium was verified. The study assesses and reports linkage disequilibrium (LD). It analyzes population structure using a validated panel of ancestry-informing markers and stratifies the population accordingly. The normality of the variables was verified. A sensitivity analysis was performed in a meta-analysis. |
| **11** | **APPROPRIATENESS OF INFERENCES DRAWN** | | Were conclusions supported by results and appropriate methods? | **7** | Justification: Appropriate conclusions. Indicates a need to replicate results. Acknowledges limitations. Interpretations consider the ancestry context. Proposes specific future studies. |
|  |  | | **TOTAL SCORE** | **68** | of 77 |
| **FINAL QUALITY RATING:** | | | | **□ Good Quality** | |

| **Article title:** | | α-syn and SNP rs356219 as a potential biomarker in blood for Parkinson's disease in Mexican Mestizos | | | |
| --- | --- | --- | --- | --- | --- |
| **First Author:** | | Salas-Leal, AC | | | |
| **Year:** | | 2021 | | | |
| **Journal:** | | Neuroscience Letters | | | |
| **Study Design:** | | Case-Control Studies | | □ Without control groups | |
|  | | | | | |
| **Item** | **Question** | | **Considerations** | **Score (1-7)** | **Notes/Comments** |
| **1** | **RATIONALE FOR STUDY** | | Was a scientific rationale for chosen genes presented to avoid selective reporting of positive results? If GWAS design (hypothesis-free approach), was rationale for selecting this design presented? | **6** | Justificación: Proporciona información que justifica el análisis de la variante analizada en el contexto del trastorno investigado (enfermedad de Parkinson esporádica). Indica el objetivo de la investigación. Justifica la realización del estudio en la población mexicana. |
| **2** | **SELECTION AND DEFINITION OF OUTCOME** | | Were cases appropriately defined? Were participants appropriately sampled to avoid selection bias? Were case/outcome assessors blinded to genotype status? If applicable, was follow-up length appropriate and attrition rate acceptable? Outcome definitions: independent adjudication/lab measures (strong) vs self-report (moderate) vs no description (poor) | **6** | Justificación: 88 cases of Parkinson's disease, evaluated by specialists using standardized diagnostic criteria. Recruitment took place at specialized clinics. Additional assessments included cognitive function (MMSE) and depression (HDRS)—limitations: Convenience sample. No specific information on ancestry is provided. It is not specified whether the clinical evaluators were blinded to the genotype. |
| **3** | **SELECTION AND COMPARABILITY OF COMPARISON GROUPS** | | Were controls appropriately defined? Were controls sampled to minimize selection bias? Was detailed description of selection procedure outlined? Were assessors of control status blinded to genotype? In multi-ethnic studies, were sub-populations (ethnicity) reported? | **4** | Justificación: The controls (n=88) were matched, with no differences by age or sex. Additional assessments included cognitive function (MMSE) and depression (HDRS). Limitations: Selection criteria (inclusion/exclusion) are not described. It is not specified whether the clinical evaluators were blinded to the genotype. The criteria for defining ethnicity are not specified. |
| **4** | **TECHNICAL CLASSIFICATION OF EXPOSURE** | | Was DNA source and storage method appropriate? Were DNA ascertainment methods similar for comparison groups? Was genotyping platform and allele-calling algorithm appropriate? Were genotyping error and call rates appropriate (≥95%)? Were genotype call rates and SNP missingness similar between groups? Was Hardy-Weinberg equilibrium tested in controls? Did authors check for outlying heterozygosity? If genotypes imputed, were methods described? | **6** | Justification: Describes the methodology for obtaining DNA. Describes the methodology used for SNP genotyping (real-time PCR with TaqMan assay). Additionally, evaluates gene expression and protein quantification. Limitations: Genotyping replication, error rate, and the use of quality controls are not mentioned. |
| **5** | **NON-TECHNICAL CLASSIFICATION OF EXPOSURE** | | Did blinded assessor conduct genotyping? Was genotyping conducted simultaneously or in batches (same methods)? If applicable, were samples randomized prior to genotyping (not all controls/cases on same plate)? | **3** | Justification: Blinding and randomization were not mentioned. |
| **6** | **OTHER SOURCES OF BIAS** | | Were all sources of bias disclosed and their effect on results discussed? (selection bias, classification bias, time-lag bias, attrition bias, etc.) | **6** | Justification: Limitations are addressed, such as possible medication bias. Sampling limitations for some geographic regions. Lack of analysis of ancestry to stratify the population. |
| **7** | **SAMPLE SIZE AND POWER** | | Was sample size appropriate? Was an a priori power analysis conducted? | **6** | Justification: Unpaired convenience sample with no significant differences in age or sex. Sufficient to detect moderate to large effects. Limitations: Statistical power calculation is not reported. |
| **8** | **A PRIORI PLANNING OF ANALYSES** | | Was analysis plan appropriate and sufficiently described? Was selective/inappropriate reporting avoided (all test results reported)? Were tested subgroups, interactions, and sensitivity analyses described? Was statistical software identified? | **6** | Justification: The analysis plan is detailed. Logistic regression and pre-specified ROC analysis are used. Subgroup analysis is performed. The software used (SPSS) is indicated. |
| **9** | **STATISTICAL METHODS AND CONTROL FOR CONFOUNDING** | | Were important confounders appropriately controlled? Was missing data handled appropriately (<10% missing acceptable)? Were results adjusted for multiple testing? For multi-ethnic studies, did statistical methods (e.g. PCA) control for confounding? | **6** | Justification: Appropriate analysis: Adjustments were made for age and sex, and multiple genetic models were tested. Limitations: Correction for multiple comparisons is not applied. |
| **10** | **TESTING OF ASSUMPTIONS AND INFERENCES** | | Were all assumptions tested? Specifically: i) If haplotypes inferred, was method reported? ii) Were distant relatives/consanguinity tested? iii) Were reported sex and ethnicity checked? | **6** | Justification: Hardy-Weinberg equilibrium proven. Performs normality tests. Limitations: Does not verify population stratification. |
| **11** | **APPROPRIATENESS OF INFERENCES DRAWN** | | Were conclusions supported by results and appropriate methods? | **6** | Justification: Appropriate conclusions with reasonable biological interpretation. The limitations are acknowledged. |
|  |  | | **TOTAL SCORE** | **61** | of 77 |
| **FINAL QUALITY RATING:** | | | | **□ Good Quality** | |
