# Supplementary material for: The genetic architecture of Parkinson's disease in Mexico: a systematic review
Source: Front Aging Neurosci. 2026 Feb 19;18:1709246. doi: 10.3389/fnagi.2026.1709246 (PMC12960540; doi:10.3389/fnagi.2026.1709246)
Supplement: Supplementary file 2 [file Table_2.docx]

# PRISMA 2020 Checklist — Genetic Architecture of Parkinson’s disease in Mexican populations.

This completed PRISMA 2020 checklist details compliance with each of the 27 reporting items as defined in the Preferred Reporting Items for Systematic Reviews and Meta-Analyses (PRISMA) 2020 statement. The table specifies the full item description, exact manuscript section or figure where the criterion is addressed, and a concise summary of how it has been satisfied in the revised version.

| Item No. | Full PRISMA 2020 Item | Location in Manuscript | Summary of Compliance |
| --- | --- | --- | --- |
| 1 | Title — Identify the report as a systematic review. | Title | The title states that this is a systematic review of Parkinson’s disease genetics in Mexican populations. |
| 2 | Abstract — Provide a structured summary including background, objectives, data sources, study eligibility, methods, results, limitations, and conclusions. | Abstract | The structured abstract follows the PRISMA for Abstracts format, with clear aims, data sources, search period, and summary findings. |
| 3 | Rationale — Describe the rationale for the review in the context of existing knowledge. | Section 1 | Provides epidemiological and ancestry rationale highlighting the underrepresentation of Latin American cohorts. |
| 4 | Objectives — Provide an explicit statement of the objectives or questions the review addresses. | Section 1 | Defines goals to map PD genetics in Mexican populations and identify ancestry-informed associations. |
| 5 | Eligibility criteria — Specify inclusion and exclusion criteria for the review and how studies were grouped for synthesis. | Section 2.2 | Inclusion and exclusion criteria are outlined, including ancestry, diagnostic criteria, study design, and available data. |
| 6 | Information sources — Specify all databases, registers, websites, organisations, reference lists, and other sources searched or consulted. | Section 2.1 | Searches were performed across PubMed, Scopus, Web of Science, and Google Scholar, along with backward citation tracking and expert input. |
| 7 | Search strategy — Present the full search strategies for all databases, including limits and filters used. | Section 2.1 | Boolean search strings and coverage dates (2004–2025) are explicitly described. |
| 8 | Selection process — Specify the methods used to decide whether a study met the inclusion criteria, including how many reviewers screened each record. | Section 2.1; Supplementary Figure 1 | Screening performed in duplicate; PRISMA 2020 flow diagram documents exclusions and final inclusion (n=25 studies). |
| 9 | Data collection process — Specify the methods used to collect data from reports, including how many reviewers collected data and whether authors were contacted. | Section 2.3 | Data extracted in duplicate using a harmonised form; genes standardised using HGNC and dbSNP identifiers. |
| 10 | Data items — List and define all outcomes, variables, and other data items sought. | Section 2.3 | Collected genotype frequencies, effect estimates, CIs, p-values, functional classifications, and sample demographics. |
| 11 | Study risk of bias assessment — Specify the methods used to assess risk of bias and how this information was used in synthesis. | Section 2.4; Figure 1 | Risk of bias assessed with Q-Genie; lower-quality studies retained but flagged for transparency. |
| 12 | Effect measures — Specify for each outcome the effect measure(s) used. | Sections 2.3; Tables 2–4 | Odds ratios (95% CI) used consistently across tables; p-values provided for all associations. |
| 13 | Synthesis methods — Describe the methods of handling data and combining results, including any subgroup analyses or sensitivity analyses. | Sections 2.3–2.4; Figures 2–3 | Qualitative synthesis employed due to heterogeneity; visualisation via Cytoscape and Looker Studio. |
| 14 | Reporting bias assessment — Describe methods used to assess risk of bias due to missing results or selective reporting. | Section 5.4 | Addresses small-sample effects, unreported negative results, and publication bias. |
| 15 | Certainty assessment — Describe methods used to assess certainty (or confidence) in the body of evidence. | Section 2.4; Table 3 | Certainty evaluated via Q-Genie score distribution and evidence strength grading (Strong–Moderate–Weak). |
| 16 | Study selection — Describe the results of the search and selection process, ideally using a flow diagram. | Section 3.1; Supplementary Figure 1 | PRISMA flowchart included; 155 records screened, 25 studies included. |
| 17 | Study characteristics — Cite each included study and present its characteristics. | Supplementary Table 1; Tables 1–2 | Presents study design, diagnostic criteria, loci examined, and variant frequencies. |
| 18 | Risk of bias in studies — Present assessments of risk of bias for each included study. | Figure 1; Section 2.4 | Q-Genie heatmap summarises methodological quality across 11 domains. |
| 19 | Results of individual studies — For all outcomes, present summary data for each study. | Tables 1–2 | Individual-level associations reported for each gene with OR, CI, and p-value. |
| 20 | Results of syntheses — Present results of all statistical syntheses; explore heterogeneity and sensitivity if applicable. | Section 3.4; Figures 2–3 | Integrative synthesis of pathways and networks using WikiPathways and GO:BP data. |
| 21 | Reporting biases — Present assessments of risk of bias due to missing results. | Section 5.4 | Acknowledges potential omission of small studies and non-significant findings. |
| 22 | Certainty of evidence — Present assessments of certainty or confidence in the body of evidence. | Tables 2–3 | Evidence categorised as Strong, Moderate, or Weak based on reproducibility and cross-validation. |
| 23 | Discussion — Provide a general interpretation of results, limitations of the evidence, and implications for future research. | Section 5 | The discussion integrates ancestry-specific findings, global comparisons, and methodological limitations. |
| 24 | Registration and protocol — Indicate whether a review protocol exists, where it can be accessed, and the registration information. | Supplementary Material | Not preregistered; reason and transparency statement provided. |
| 25 | Support — Describe sources of financial or non-financial support for the review. | Acknowledgements | No funding influenced study design, data collection, or interpretation. |
| 26 | Competing interests — Declare any competing interests of review authors. | Declarations | No competing interests declared. |
| 27 | Availability of data, code, and other materials — Report which materials are publicly available and where they can be found. | Supplementary Material | All Q-Genie scores, extracted tables, and analytic scripts are available as supplementary information. |
