# Supplementary material for: The genetic architecture of Parkinson's disease in Mexico: a systematic review
Source: Front Aging Neurosci. 2026 Feb 19;18:1709246. doi: 10.3389/fnagi.2026.1709246 (PMC12960540; doi:10.3389/fnagi.2026.1709246)
Supplement: Supplementary file 3 [file Table_3.docx]

***Complete Database-Specific Search Strategies***

A comprehensive literature search was undertaken to identify all peer-reviewed genetic association and transcriptomic studies involving Parkinson’s disease (PD) cases recruited in Mexico. Searches were conducted across PubMed/MEDLINE, Scopus, Web of Science Core Collection, and Google Scholar, covering the period from January 1 2004, to February 28 2025. This time frame reflects the period during which SNP-based genotyping and molecular assays became routinely accessible in Mexican research settings. In accordance with PRISMA 2020 Item 7, the full database-specific search strategies, including controlled vocabulary, Boolean logic, field tags, and free-text synonyms, are documented below to ensure complete reproducibility.

*PubMed/MEDLINE*

The PubMed search combined MeSH terms with free-text terms to maximise sensitivity. Terms for Parkinson’s disease (“Parkinson Disease,” “Parkinson’s disease,” “Parkinson disease,” “PD”) were paired with genetic descriptors (“genetic variant,” “mutation,” “single nucleotide polymorphism,” “SNP,” “polymorphism,” “copy number variation,” “CNV,” “gene expression,” “transcriptomic”). These were combined with geographical identifiers (“Mexico,” “Mexican,” “mestizo”) using Boolean operators. Automatic Term Mapping remained enabled to capture indexed synonyms.

*Scopus*

The Scopus search was conducted within the TITLE-ABS-KEY fields to ensure that studies explicitly referring to PD genetics were captured. Parkinson’s disease terms were combined with genetic and molecular search terms (including variants, mutations, SNPs, CNVs, and transcriptomic profiling) and recruitment-related terms (“Mexico,” “Mexican,” “mestizo”). No document-type or subject-area filters were applied to avoid excluding biomedical studies indexed outside clinically focused categories.

*Web of Science core collection*

The Web of Science search used the Topic (TS) field, which indexes titles, abstracts, author keywords, and KeyWords Plus. Parkinson’s disease–related terminology was combined with genetic and transcriptomic descriptors and Mexico-related recruitment identifiers using Boolean operators. Both the Science Citation Index Expanded and the Emerging Sources Citation Index were included to ensure comprehensive coverage.

*Google scholar*

Because Google Scholar does not support Boolean operators or field-restricted searches, a reproducible free-text string consisting of key terms related to Parkinson’s disease, genetics, Mexico, and variant analysis was used. Following best-practice recommendations for reproducibility, the first 200 results sorted by relevance were screened. This approach balances sensitivity with methodological standardisation across searches.

*Additional search methods*

Backward citation tracking was conducted for all included studies to identify earlier relevant publications, and forward citation tracking was performed using Web of Science and Google Scholar to identify later studies citing included articles. Grey literature, dissertations, and non-peer-reviewed documents were intentionally excluded to maintain methodological consistency and ensure that the synthesis reflected peer-reviewed scientific evidence.

*Search execution and documentation*

All searches were executed on a single calendar day to avoid discrepancies introduced by incremental database indexing updates. Search logs, export files, and deduplication records were manually maintained and verified. These procedures ensured a transparent, reproducible foundation for subsequent screening and selection.
